# Integrated Methylome and Transcriptome Analysis Widen the Knowledge of Cytoplasmic Male Sterility in Cotton (*Gossypium barbadense* L.)

**DOI:** 10.3389/fpls.2022.770098

**Published:** 2022-04-27

**Authors:** Jingyi You, Min Li, Hongwei Li, Yulin Bai, Xuan Zhu, Xiangjun Kong, Xiaoyan Chen, Ruiyang Zhou

**Affiliations:** ^1^Key Laboratory of Plant Genetics and Breeding, College of Agriculture, Guangxi University, Nanning, China; ^2^Xinjiang Yida Textile Co., Ltd, Urumqi, China; ^3^Dali Bai Autonomous Prefecture Agricultural Science Extension Institute, Dali, China; ^4^School of Life Science and Technology, Henan Institute of Science and Technology, Xinxiang, China

**Keywords:** cotton, DNA methylation, cytoplasmic male sterility, methylated gene, transcriptome

## Abstract

DNA methylation is defined as a conserved epigenetic modification mechanism that plays a key role in maintaining normal gene expression without altering the DNA sequence. Several studies have reported that altered methylation patterns were associated with male sterility in some plants such as rice and wheat, but global methylation profiles and their possible roles in cytoplasmic male sterility (CMS), especially in cotton near-isogenic lines, remain unclear. In this study, bisulfite sequencing technology and RNA-Seq were used to investigate CMS line 07-113A and its near-isogenic line 07-113B. Using integrated methylome and transcriptome analyses, we found that the number of hypermethylated genes in the differentially methylated regions, whether in the promoter region or in the gene region, was more in 07-113A than the number in 07-113B. The data indicated that 07-113A was more susceptible to methylation. In order to further analyze the regulatory network of male sterility, transcriptome sequencing and DNA methylation group data were used to compare the characteristics of near-isogenic lines 07-113A and 07-113B in cotton during the abortion stage. Combined methylation and transcriptome analysis showed that differentially expressed methylated genes were mainly concentrated in vital metabolic pathways including the starch and sucrose metabolism pathways and galactose metabolism. And there was a negative correlation between gene methylation and gene expression. In addition, five key genes that may be associated with CMS in cotton were identified. These data will support further understanding of the effect of DNA methylation on gene expression and their potential roles in cotton CMS.

## Introduction

Cotton is cultivated throughout the globe for fiber and oil seed production (Campbell et al., 2010). However, low yield is still one of the key factors hindering its further development. Heterosis as an effective method that is being widely applied to increase yield and improve cotton quality. Cytoplasmic male sterility (CMS) is a common maternally inherited phenomenon in plants, which greatly facilitates the production of hybrid seeds and utilization of heterosis on a large scale (Bahadur et al., 2015), including cotton (Yang et al., 2014, 2018), kenaf (Liao et al., 2016; Zhou et al., 2017), Pummelo (Zheng et al., 2012), rape (*Brassica napus*; Yan et al., 2013), and chili pepper (*Capsicum annuum* L.; Liu et al., 2013b). Previous studies have revealed that CMS is a consequence of interactions between mitochondrial genes and nuclear genes (Chen and Liu, 2014).

The successful construction of three cotton lines, including a male-sterile line (A), its maintainer line (B), and restoring line (R), provides a functional breeding tool for harnessing heterosis utilization in plants. Research regarding CMS molecular mechanisms in plants has been a hot and difficult topic at home and abroad for some years. However, the molecular mechanism controlling CMS in cotton remains unclear. Many studies indicate that CMS-associated genes are derived from the mitochondrial genome, and the restorer of fertility (Rf) genes that control fertility restoration in F_1_ hybrids are located in the nuclear genome (Budar et al., 2003; Horn et al., 2014; Hu et al., 2014). More than 50 mitochondrial genes have been identified as CMS-relevant in various plants (Dewey and Levings, 1987; Song and Hedgcoth, 1994; Handa et al., 1995), such as WA352 in wild-abortive CMS rice (Wang et al., 2018) and rf2 in T-cytoplasm maize (Cui et al., 1996). The near-isogenic lines are similar at the nuclear gene level and therefore are cytoplasmic near-isogenic lines. These lines can effectively eliminate interference by extraneous genetic information irrelevant to CMS. Furthermore, growing evidence has supported roles for programmed cell death and reactive oxygen species in the CMS pathway (Balk, 2001; Wan et al., 2007; Ji et al., 2013; Yang et al., 2014). Line 07–113 is a male-sterile plant discovered in the detached ceiba (perennial island cotton) of successive generations of self-cross progeny introduced by our research group from the Cotton Research Institute of the Chinese Academy of Agricultural Sciences. Line 07–113 was used as the female parent saturated backcross with its sister line 07-113B. After multiple backcrosses, the CMS line 07-113A was produced in 2017, and its sister line for backcrossing is the maintainer line 07-113B. Although some mitochondrial genomes of cotton have been reported so far (Liu et al., 2013a; Li et al., 2018), there has been relatively little progress into the study of CMS-related genes in cotton.

In general studies, DNA methylation mainly refers to methylation of the fifth carbon atom on cytosine in a CpG dinucleotide. Its product is called 5-methylcytosine (5-mc), which is the main form of DNA methylation in eukaryotes such as plants and animals. As a preserved epigenetic modification mechanism, DNA methylation is stable and heritable and can regulate gene expression without altering the DNA sequence. However, it may change in response to developmental and environmental cues (Grafi, 2011). In plants, DNA methylation has been identified in all different sequence contexts, including CG, CHG, and CHH (H represents either A, T, or G; Chan et al., 2005). Clear evidence suggests that DNA methylation affects gene expression and cell function through a series of biological processes (BPs), including transposon silencing, DNA repair, genomic imprinting, and gene transcription (Handa et al., 1995; Campbell et al., 2010; Grafi, 2011). Emerging evidence demonstrates that dynamic changes in DNA methylation may mediate transcriptional variation during male reproductive development (Slotkin et al., 2009). Furthermore, some studies indicate that CMS is associated with DNA methylation in plants, including rice (Xu et al., 2013), wheat (Ba et al., 2014), maize (Chen et al., 2016), cotton (Ma et al., 2018a; Zhang et al., 2019b), cabbage (Han et al., 2017), and tomato (Vahid et al., 2015). However, DNA methylation associated with CMS in cotton has received little attention so far.

The transcriptome is the inevitable link between genomic genetic information and the proteome of biological functions; the transcriptional level is by far the most studied and the most important mode of regulation in organisms. Transcriptome sequencing (RNA-Seq) can provide the expression information of thousands of genes in a single experiment and has greatly contributed to the development of functional genomics. With the development of high-throughput sequencing and bioinformatic technologies, CMS-related genes have been identified through the construction of transcriptional regulatory networks, which are related to the tricarboxylic acid (TCA) cycle, oxidative phosphorylation, respiratory electron transport chain, toxic proteins, and carbohydrate metabolism (Wu et al., 2013; Liao et al., 2016; Mei et al., 2016; Kong et al., 2017a; Chen et al., 2018; Tang et al., 2018; Li et al., 2019, 2021; Ning et al., 2019; Zheng et al., 2019; Zhang et al., 2021). Therefore, comparative transcriptome analysis has been widely used to study the molecular mechanisms of CMS in plants, including the red flax CMS line P3A (Chen et al., 2014), the soybean CMS line NJCMS1A (Li et al., 2015a), the Welsh onion CMS line 64-2 (Liu et al., 2016), and the cabbage CMS line PM (Wang et al., 2016).

In this study, we combined bisulfite sequencing (BS-Seq) and RNA-Seq technologies to investigate the potential relationship between DNA methylation and pollen abortion in CMS line 07-113A and its maintainer line 07-113B. The validated hypothesis was that abnormal DNA methylation affects gene expression and is involved in cotton CMS pollen abortion. These data will provide a basis for further understanding of the molecular mechanisms of DNA methylation in cotton CMS.

## Materials and Methods

### Plant Materials and Treatment

Cotton CMS line 07-113A was bred from a mutant of 07-113 by saturation backcrossing. After several years of backcrossing, 07-113A and 07-113B had a similar nuclear genetic background and formed a pair of near-isogenic lines. The plant materials were cultivated at the agricultural farm of Guangxi University, Nanning, Guangxi, China. Our previous cytological observations showed that pollen abortion occurred roughly at the tetrad stage (4 mm–5 mm in diameter). Floral buds at the tetrad stage were collected and frozen in liquid nitrogen and stored at −80°C in a freezer for further determination.

### DNA and RNA Extraction, Quantification, and Purification

Total genomic DNA was isolated from the floral buds of cotton with DNeasy Plant Mini Kit (Qiagen, CA, United States). Total RNA from the floral buds (tetrad stage) of 07-113A and 07-113B was extracted using a Quick RNA Isolation Kit (Huayueyang, Beijing, China) following the manufacturer’s protocol. A NanoDrop 2000 spectrophotometer and an Agilent 2100 Bioanalyzer were used to check the DNA and RNA concentration and integrity. After filtering, we obtained the clean data. RNA samples that had a 260/280 ratio above 2.0 were selected for use in subsequent processes.

### DNA Methylation Library Construction and Data Analysis

For normal whole-genome bisulfite sequencing (WGBS) library construction, Genomic DNA was broken into fragments with an average size of 250 bp using a Bioruptor (Diagenode, Belgium), and then, the end of DNA fragments was repaired, and A base was added at the 3′ end to connect the methylation junction. Bisulfite was treated using an EZDNA Methylation-Goldkit (ZYMO). Fractions of different insertion sizes were extracted from the same channel of 2% TAE agarose gel. The qualified library was obtained for sequencing after PCR amplification. Clean data were mapped to the reference genome by BSMAP, after making sure the quantity of clean data were sufficient after filtering the raw data. These uniquely mapped data were then used to obtain methylation information of cytosine through the whole genome, after which duplication reads were merged and the mapping results were removed according to each library. Methylation level was determined by dividing the number of reads covering each mC by the total reads (more than four reads effectively) covering that cytosine, which was also equal the mC/C ratio at each reference cytosine (Lister et al., 2009; Xiang et al., 2010). The different methylated region (DMR) from different samples was calculated by tDMR (Li et al., 2010), which was developed by BGI (Beijing Genomics Institute) as mentioned already. Putative DMRs were identified by comparison of the sample1 and sample2 methylomes using windows that contained at least five CpG (CHG or CHH) sites with a 2-fold change in Methylation level and Fisher test value of *p* ≤ 0.05. Otherwise, the two DMRs were viewed as independent. After iteratively merging interdependent DMRs, the final dataset of DMRs was made up of those that were independent from each other. The gene element annotation of methylated region or DMR from different samples was carried out as well. A comparison of windows containing at least five CGs (CHG or CHH) at the same location in the genomes of the two samples was made to obtain putative differentially methylated regions (DMRs) with a 2-fold change. Methylation level and Fisher’s test value of *p* ≤ 0.05. We used CIRCOS to compare the difference in methylation levels of DMR between samples to calculate the difference in methylation levels between the two samples and the difference in methylation levels at a site between the two samples can be calculated using the following equation:


$$\mathrm{Degree of difference}=\log_{2}\left(Rm_{1}/Rm_{2}\right)$$

*Rm*_1_ and *Rm*_2_ represent the methylation levels of mC of samples 1 and 2, respectively. If the value of *Rm*_1_ or *Rm*_2_ is 0 then use 0.001 instead.

Subsequently, the genes located in DMRs, called differential methylated genes (DMGs), were characterized.

### cDNA Library Construction and Data Analysis

Total RNA was processed by mRNA enrichment or rRNA division:magnetic beads with oligo-dT were used to enrich mRNA with poly-A tails; RNase H was used to selectively digest the DNA/RNA hybridization strand and then digest the DNA probe with DNase I and subsequently purify it to obtain the required RNA. The obtained RNA was segmented using interrupting buffer, the random N6 primers were reverse-transcribed; and then, the two strands of cDNA were synthesized to form double-stranded DNA. The end of the synthesized double-stranded DNA was smoothed and phosphorylated at the 5′ end. The 3′ end formed a sticky end with a protruding “A,” and then a bubble-like connector with a protruding “T” at the 3′ end was connected. The ligation products were amplified by PCR with specific primers. The PCR product was thermally denatured into single strands; then, a bridge primer was used to loop them into a circular DNA library for sequencing.

The filter software SOAPnuke, independently developed by BGI (Shenzhen, China), was used for statistics, and the filter software Trimmomatic was used for filtering. Reads with low quality joint contamination and unknown base N content >5% were filtered to ensure the reliability of the results. We then used HISAT to compare clean reads to the cotton reference genome,^1^ and Bowtie2 (Langmead and Salzberg, 2012) to compare clean reads to the reference gene sequence. We then used RSEM (Dewey and Li, 2011) to calculate the expression levels of genes and transcripts. Finally, we added new transcripts with protein-coding potential to the reference gene sequence to form a complete reference sequence and then calculated the gene expression level.

### DEG Identification and Gene Functional Annotation Analysis

Differentially expressed genes (DEGs) were detected using the Deseq2 method (fold change ≥2.00 and adjusted *p* value ≤0.05) based on the principle of negative binomial distribution and DEG detection using the previously described protocol (Anders and Huber, 2010; Love et al., 2014). According to the results of differential gene detection, the union of differential genes was analyzed by hierarchical clustering using the PHEATMAP function in R software. According to Gene ontology (GO) and Kyoto Encyclopedia of Genes and Genomes (KEGG) annotation results and official classification, functional classification of the differential genes was performed. Meanwhile, phyper function in R software was used for enrichment analysis to calculate a *p* value, and then, FDR correction was performed on the *p* value; usually a *Q*-value ≤0.05 was considered as significant enrichment.

### Validation of DMGs Through Quantitative Real-Time PCR

A PrimeScript RT Reagent Kit (Takara, Dalian, China) was used to reverse RNA into cDNA to verify the relative expression of the DEGs by using quantitative reverse transcription polymerase reaction (qRT-PCR). The 18 s gene of cotton served as the endogenous control. All the primers were designed by Premier 5.0 and synthesized by BGI (Shenzhen, China). Gene expression levels were determined in triple biological repeats as previously described (Kong et al., 2017b).

### GO and KEGG Enrichment Analysis of DMEGs

Differentially expressed genes associated with DMRs (DMEGs) were screened out by GO analysis and KEGG enrichment analysis, using the GO database,^2^ and the KEGG database for enrichment analysis.^3^ GO terms or KEGG pathways with a corrected *p* value ≤0.05 were considered significantly enriched.

## Results

### Phenotypic Characterization of 07-113A and 07-113B

**Figure 1:**
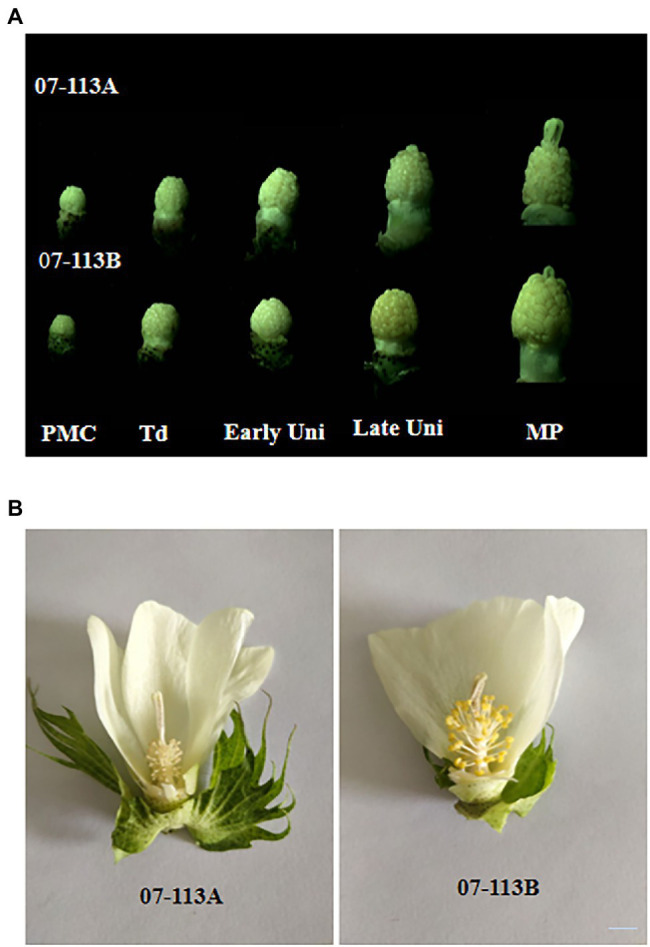
Comparison of the morphological characteristics among 07-113A and 07-113B. **(A)** Morphological characteristics of anther development of 07-113A and 07-113B in cotton. **(B)** The flower morphological characteristics of 07-113A and 07-113B in cotton. PMC, pollen mother cell; Td, Ttrad; Early Uni, early uninucleate; Late Uin, late uninucleate; and MP, mature pollen. Bar = 0.5 cm.

Floral organs play an important role in plant reproduction. A stamen abnormality, in which pollen is absent or the anthers do not undergo dehiscence usually results in CMS. In the process of microspore development, the floral organ phenotypes of CMS line 07-113A and its maintainer line 07-113B were basically the same. However, there were obvious differences in the anthers. The anthers of 07-113B were normally full and powdery, and pollen grains were released after dehiscence while the anthers of 07-113A were shriveled (Figure 1).

### Analysis of Genome-Wide DNA Methylation Data of 07-113A and 07-113B

**Figure 2:**
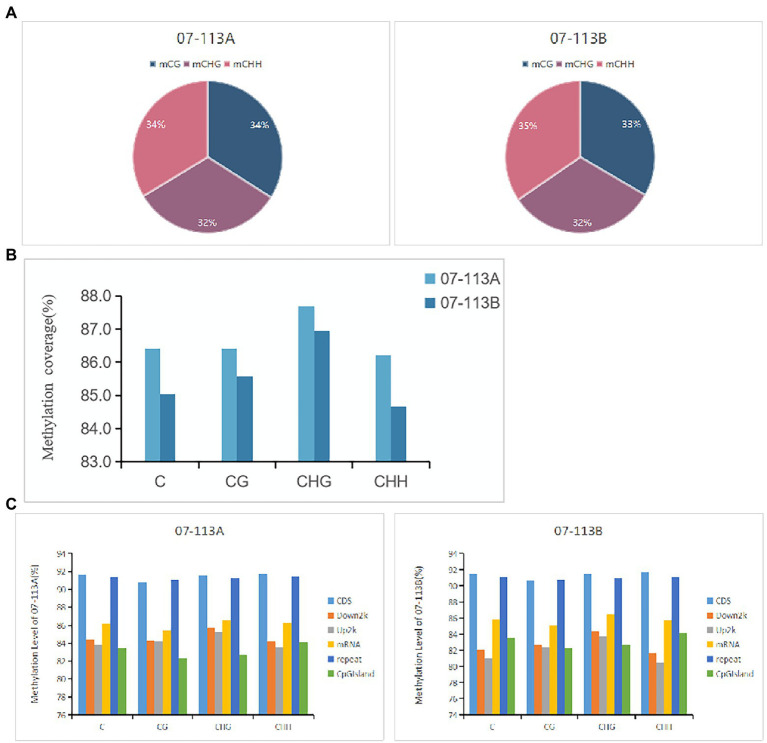
Comparative analysis of DNA methylation patterns between 07-113A and 07-113B. **(A)** Distribution of methylated cytosines in the three contexts of 07-113A and 07-113. **(B)** Methylation coverage in each sequence context across the whole cotton genome. **(C)** The coverage of methylation level in various genomic regions.

To explore the potential role of DNA methylation in cotton pollen abortion, the floral buds (tetrad stage) of the CMS line 07-113A and its maintainer line 07-113B were collected for constructing genomic DNA libraries. We sequenced two samples for *Gossypium_barbadense* species using WGBS technology, generating on average 84.660 Gb clean bases after filtering low quality reads, N reads, and adaptor sequences. Table 1 summarizes sequencing data for each sample. According to the sequence context of cytosine, cytosine in the DNA sequence can be classified into three types: CG, CHG, and CHH (H = A, G, or T). Next, these mapped data were used to retrieve the methylation level (ML) of each cytosine site in the CG, CHG, and CHH contexts. The percentages of mCG, mCHG, and mCHH in the whole genome of 07-113B and 07-113A were calculated in the corresponding cytosine environment. In 07-113B, the number of methylated cytosine sites in the three contexts was 34,500,648 mCG sites (33.3.0% of all mC), 33,334,348 mCHG cites (32.20% of all mC), and 35,672,033 mCHH sites (34.5% of all mC), respectively. Similarly, there were 34,496,729 mCG sites (33.9% of all mC), 33,038,776 mCHG sites (32.5%of all mC), and 34,112,831 mCHH sites (33.6% of all mC) in 07-113A (Figure 2A). Meanwhile, we counted the coverage of cytosine sites in the whole genome in the samples and found that 07-113A had 86.42, 87.69%, and 86.22 coverage on mCG, mCHG, and mCHH, respectively, and its genomic mC coverage was 86.41%; the corresponding values in 07-113B were 85.58, 86.95, and 84.67%, respectively, and its genomic mC coverage was 85.04%. Their genomic mC coverage was 85.04% (Figure 2B). In order to further compare the two samples, we also analyzed the whole-genome methylation level within the scope of various genomic elements, including CDS, Down2k, Up2k, mRNA, repeat sequences, and CpG island. Obviously, both 07-113A and 07-113B have relatively high methylation levels in the CDS region, repeat region, and CpG-island region, indicating that these are epigenetic regulation regions that may be related to the regulation of gene expression (Figure 2C).

**Table 1:** Sequence reads generated by whole-genome BS-Seq in 07-113A and 07-113B.

Sample ID	Clean reads number	Clean data size (bp)	Mapped reads	Mapping rate (%)	Bisulfite conversion rate (%)
07-113B	885,735,204	88,573,520,400	708,087,333	79.94	99.25
07-113A	807,460,564	80,746,056,400	628,880,686	77.88	99.52

### Correlation Between DNA Methylation Patterns and Differential Gene Expression

To understand the potential role of DNA methylation in the development of cotton anthers, we first performed GO analysis of DMGs. We found 448 genes that were both hypermethylated and highly expressed, and these genes were classified into 1,605 functional categories, including 469 biological processes (BPs), 645 cellular components (CCs), and 491 molecular functions (MFs; Supplementary Figure S1). Here, 441 hypomethylated DEGs (hypo-DEGs) were annotated to 1,923 functional categories, including 480 BPs, 581 CCs, and 485 MFs (Supplementary Figure S2).

**Figure 3:**
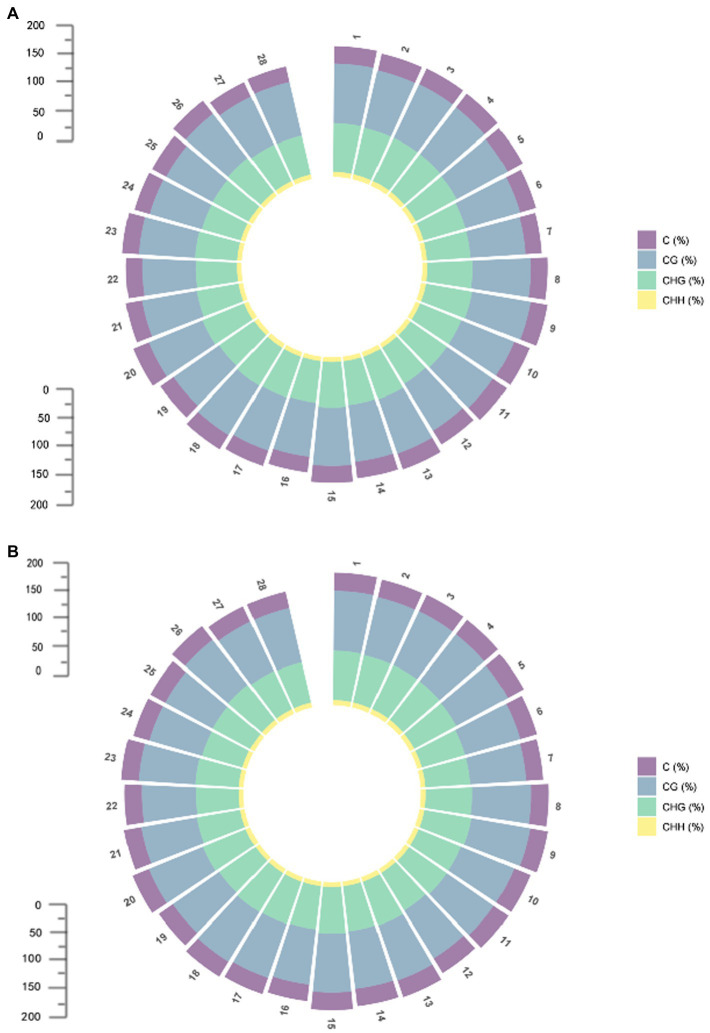
Methylation distribution of each chromosome; **(A)** Methylation distribution of each chromosome in 07-113A. **(B)** Methylation distribution of each chromosome in 07-113B.

Differentially methylated regions (DMRs) are certain DNA segments in different samples where the genome exhibits different methylation patterns. DMRs are associated with genetic imprinting and exhibit a methylation status in individuals that is consistent with that of the parent or dam. DMRs play an important role in epigenetic variation by modifying biological processes through regulation of gene expression. Abnormally increased methylation levels can trigger abnormalities in a variety of biological processes. In the comparison between 07-113A and 07-113B, a total of 20,774 DMRs were identified, including 11,405 differential regions in CG mode, 9,328 differential regions in CHG mode, and 41 differential regions in CHH mode (Supplementary Table S1). It can be seen that DMRs are mainly concentrated in CG and CHG conditions. The situation on each chromosome is consistent with this result (Figure 3). We divided DMRs into two categories according to the sequence conditions: (1) in CG mode and (2) in CHG mode. Functional annotation of these two types of DMRs using GO and KEGG databases, respectively, showed that the number of total DMRs, on the other hand, was the least in biological processes and the most in functions, which indicates that there are multiple genes involved in different biological processes, but DMRs are more involved in molecular functions (Supplementary Figures S3, S4).

**Figure 4:**
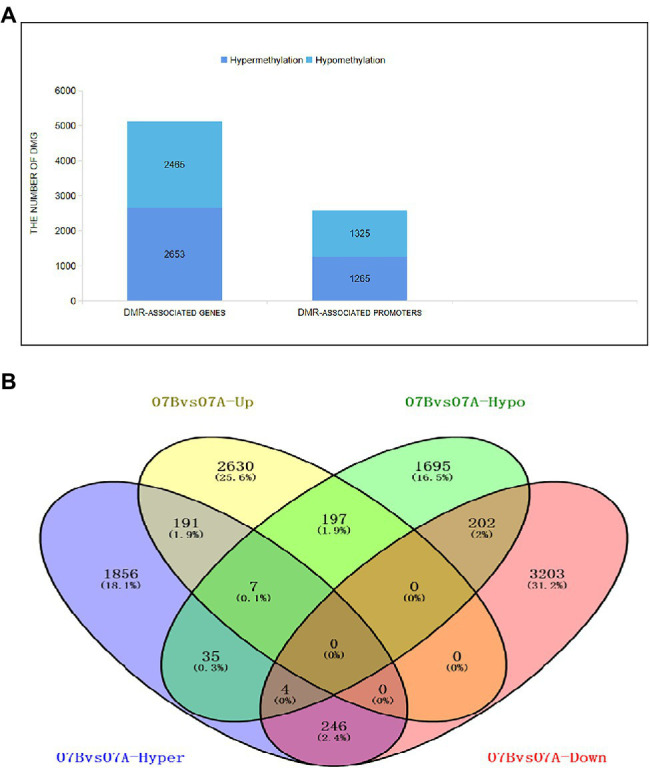
Distribution of differentially methylated genes. **(A)** Distribution of differentially methylated genes in DMR-associated genes/promoter groups between 07-113A and 07-113B. **(B)** Venn diagram showing overlaps between the differential methylated genes (DMGs) and differentially expressed genes (DEGs). 07A, 07-113A; 07B, 07-113B.

In this study, we also classified DMGs into two subgroups: DMR-associated genes and DMR-associated promoters. The results showed that there were 5,118 DMRs associated with genes and 2,586 DMRs associated with promoters, and the number of hypermethylated DMGs was higher than that of hypomethylated DMGs (Figure 4A; Supplementary Table S2). Data showed that 07-113A was more prone to hypermethylation than 07-113B. To further explore the possible link between DNA methylation and gene expression, we examined the overlap between DMGs and DEGs. In total, 3,025 upregulated genes and 3,655 downregulated genes were found, of which 2,653 were upregulated in the hypermethylated genes and 2,465 were downregulated in the hypomethylated genes (Figure 4B).

### GO and KEGG Enrichment Analysis of DMEGs

**Figure 5:**
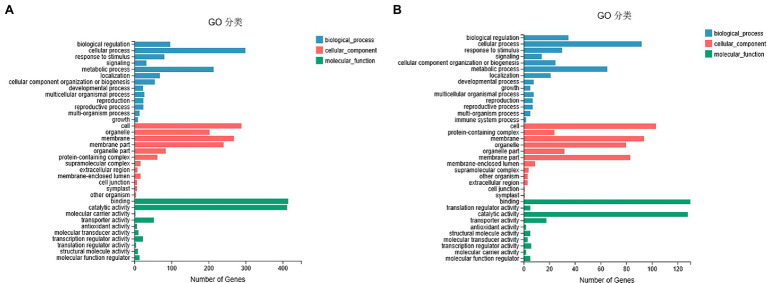
Gene ontology classification of DEGs in differentially methylated region (DMR)-associated genes **(A)**/promoters **(B)** groups between 07-113A and 07-113B.

**Figure 6:**
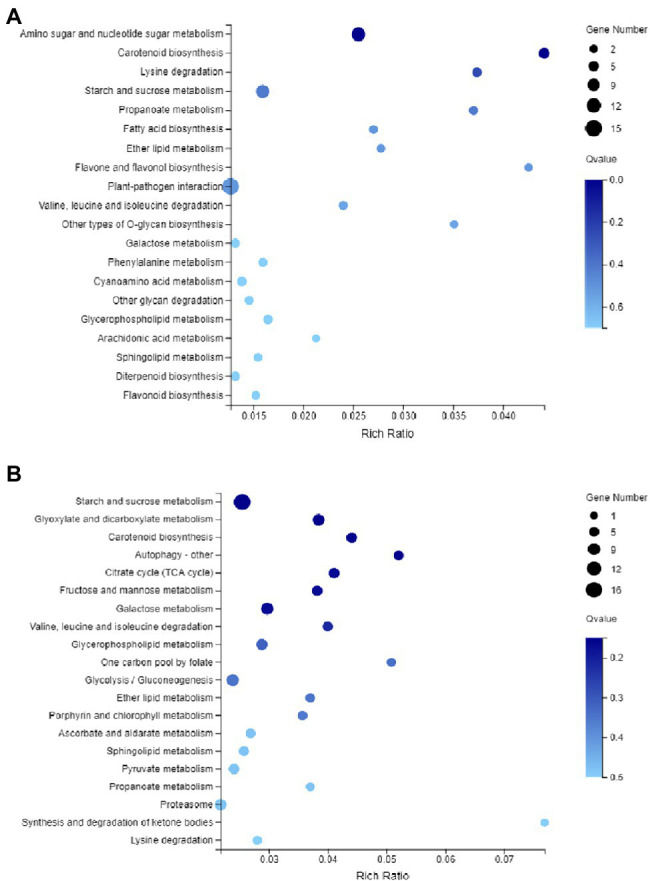
Kyoto Encyclopedia of Genes and Genomes (KEGG) pathway enrichment analysis of DMEG. **(A)** KEGG of genes in the differentially methylated region (DMR)-associated promoters. **(B)** KEGG of genes in the DMR-associated genes, and the color of the circle represents the *Q*-value (corrected *p* value).

To understand the potential role of DNA methylation changes in cotton anther development, we first performed GO analysis of DMEGs. We found that there were 847 DEGs in DMR-associated genes (Supplementary Table S3), and these genes were annotated into a total of 35 functional categories, namely, 13 BPs, 12 CCs, and 10 MFs (Figure 5A). Among these biological activities, DMGs are mainly involved in cellular processes, metabolic processes, membranes, membrane fractions, catalytic activity, and binding. Similarly, in the DMR-associated promoters group, 310 DMEGs were annotated to 36 functional categories, namely, 14 BP, 12 CC, and 10 MF (Figure 5B). Surprisingly, we found that both subgroups were involved in similar biological processes. Similarly, KEGG enrichment analysis was performed in the hyper-DEGs of 07-113B and 07-113A, and the top two enriched pathways in the DMR-associated promoters group were the “plant-pathogen interaction” and “starch and sucrose metabolism” pathways (Figure 6A; Supplementary Table S4). In the DMR-associated genes subgroup, the “starch and sucrose metabolism” pathway was the most enriched pathway, followed by the “galactose metabolism” pathway and the “glycolysis and glycolytic synthesis” pathway (Figure 6B; Supplementary Table S5), with a corrected value of *p* ≤ 0.05.

Previous studies reported that changes in mitochondrial genome structure and sequence in higher plants are closely related to plant CMS (Hanson and Bentolila, 2004) and that mitochondria are the main sites of eukaryotic energy metabolism, whose main function is to perform the TCA cycle and oxidative phosphorylation for the synthesis of ATP to provide direct energy for life activities. Therefore, we then focused on the analysis of DMEGs involved in the oxidative phosphorylation signaling pathway and TCA pathway identified in two comparative assemblies of 07-113A and 07-113B. As a result, six genes were found to be enriched in 07-113A and 07-113B, respectively (Supplementary Tables S6, S7). Transcriptome sequencing data showed that a total of four genes were significantly expressed in 07-113B, including GOBAR_AA36705, GOBAR_AA26633, GOBAR_AA39007, and GOBAR_AA30876, of which GOBAR_AA30876 is a v-type proton ATPase subunit A, mainly responsible for ATP hydrolysis, driving cationic transport through the membrane, thus promoting the absorption of nutrients such as amino acids. GOBAR_AA36705 encodes aconitate hydratase. Aconitase exists in the inner membrane of mitochondria and easily binds with lipophilic NO, leading to the inactivation of various enzymes in mitochondria, causing inhibition of mitochondrial respiration and cell death.

### Validation of DMEGs by qRT-PCR

**Figure 7:**
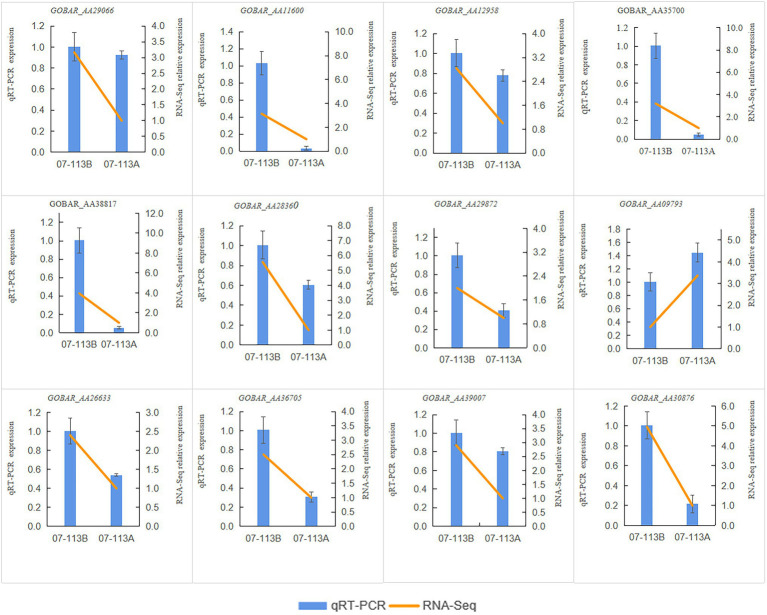
RT-qPCR verification results of 12 genes. The relative expressions levels of the 12 genes were calculated using the 2^-△△Ct^ method.

In order to investigate the effect of methylation on male sterility in cotton, genes involved in “starch and sucrose metabolism,” “oxidative phosphorylation,” and “tricarboxylic acid cycle” in 07-113B and 07-113A were subsequently selected for further analysis. Notably, 12 genes showed the largest fold change (>2-fold; Supplementary Tables S6–S8). Interestingly, 11 of these genes, namely, *GOBAR_AA11600*, *GOBAR_AA29066*, *GOBAR_AA35700*, *GOBAR_AA38817*, *GOBAR_AA29872*, *GOBAR_AA28360*, *GOBAR_AA12958*, *GOBAR_AA36705*, *GOBAR_AA26633*, *GOBAR_AA39007*, and *GOBAR_AA30876* were downregulated in CMS line 07-113A while only one gene was upregulated, namely, *GOBAR_AA09793*. To investigate the effect of DNA methylation on gene expression, we examined the transcript levels of these 12 genes in 07-113B and 07-113A using qRT-PCR. Overall, the qRT-PCR expression trends of these 12 genes were consistent with the results of RNA-Seq (Figure 7). This indicates the reliability of the transcriptome data. And among these 12 genes, except the genes *GOBAR_AA30876* and *GOBAR_AA0979*3, the methylation levels were consistent with the trend of gene expression levels, the remaining 10 genes showed opposite methylation level trends. This indicated a negative correlation between methylation and gene expression. Among them, we identified five genes most likely to be associated with CMS, namely, *GOBAR_AA11600*, a gene belonging to the hexokinase family; *GOBAR_AA29872*, a gene homologous to the Arabidopsis hexokinase superfamily protein KORRIGAN 2 (KOR2); *GOBAR_AA28360*, a gene involved in pollen outer wall formation; *GOBAR_AA36705*, a gene belonging to the aconitase/IPM isomerase family; and a polypeptidase gene encoding V-type proton ATPase subunit a3, namely, *GOBAR_AA30876* (Table 2).

**Table 2:** Five genes most likely to be associated with CMS.

Gene ID	Pathway name	Level
*GOBAR_AA11600*	ko01110//Biosynthesis of secondary metabolites;ko00051//Fructose and mannose metabolism;	Carbohydrate metabolism; Global and overview maps
*GOBAR_AA28360*	ko00500//Starch and sucrose metabolism	Carbohydrate metabolism
*GOBAR_AA29872*	ko01100//Metabolic pathways;ko00500//Starch and sucrose metabolism	Carbohydrate metabolism; Global and overview maps
*GOBAR_AA30876*	ko01100//Metabolic pathways; and ko04145//Phagosome;ko00190//Oxidative phosphorylation	Energy metabolism; Global and overview maps; Transport and catabolism
*GOBAR_AA36705*	ko01200//Carbon metabolism;ko00630//Glyoxylate and dicarboxylate metabolism;ko01110//Biosynthesis of secondary metabolites;ko00020//Citrate cycle (TCA cycle); and ko01230//Biosynthesis of amino acids;	Global and overview maps; Carbohydrate metabolism

## Discussion

### Relationship Between DNA Methylation and Cytoplasmic Male Sterility in Plants

Epigenetics are a phenomenon in which the DNA sequence remains unchanged, but gene expression is altered. DNA methylation is one of the most deeply studied and important epigenetic modifications; many studies have reported its very close association with genome stability, gene transcriptional regulation, and disease development. Several genome-wide DNA methylome studies have shown that epigenetics plays an important role in gene regulation during seed germination in *Arabidopsis*, maize, and soybean (Hsieh et al., 2009; Song, 2013; Li et al., 2015b). These results suggest that gene expression may be influenced by epigenetic modifications. Whole-genome sulfite sequencing can determine methylation patterns at single-base resolution (Harris et al., 2010). Recently, WGBS has been applied to decrypt increasing numbers of plant methyl bodies from *Arabidopsis* to rice and cotton (Cokus et al., 2008; Lister et al., 2008; Zhang et al., 2015; Feng et al., 2016; Song et al., 2017). In this study, genome-wide DNA methylation patterns were described in the CMS line 07-113A and its near-isogenic line 07-113B using WGBS to investigate the potential relationships between DNA methylation and cotton pollen abortion.

Previous studies have shown that aberrant DNA methylation genes impair the development of pollen and flowers and are associated with CMS in plants (Li et al., 2017; Han et al., 2018). Studies have shown that epigenetic regulatory mechanisms play an important role in regulating the expression of relevant flowering genes (Khan et al., 2014). As early as 1993, Burn et al. treated *Arabidopsis* with the DNA methylation inhibitor 5-A07-113Ac (a cytosine analogue), which reduced genomic methylation levels and led to a reduction in FLC expression, resulting in earlier flowering in *Arabidopsis* (Burn et al., 1993). Previous studies have shown that methylation occurs in promoter and intron regions, while DNA methylation in the mulberry genome occurs mainly in intergenic and exonic regions (Li et al., 2020); in addition, many intergenic regions also tend to be highly methylated, while most promoter regions have low methylation levels. In our study, the number of hypermethylated genes occurring in the methylation region was more in 07-113A than in 07-113B, both in the gene and promoter regions, which indicated that 07-113A was more susceptible to hypermethylation than 07-113 B.

These results suggest that the DNA methylation pattern of cotton is similar to that of other plants and that there may be some degree of conservation in the DNA methylation pattern of different plants. It is generally believed that DNA methylation, a major transcriptional silencing pathway, is negatively correlated with gene expression levels in plants (Polansky et al., 2008). However, some studies have also shown that DNA methylation is positively correlated with gene expression. Transcriptome analysis of mulberry at different developmental stages has shown that some genes have higher DNA methylation levels along with higher expression (Li et al., 2020). In our study, a total of 847 DMEGs were identified by associated analysis of methylomics and transcriptomics. Among them, 198 genes were upregulated and 250 genes were downregulated in the hyper-DEGs. In the hypo-DEGs, 204 genes were upregulated and 206 genes were downregulated (Supplementary Tables S9, S10). This result suggests that the occurrence of methylation suppresses gene expression. KEGG analysis of this DMEG showed that the hyper-DEGs of 07-113B and 07-113A were significantly enriched in the “starch and sucrose metabolic” pathway (Figure 6A and Supplementary Table S11), followed by the “galactose metabolic” pathway.

### Some Possible Genes Involved in the Development of Male Sterility

In the process of microspore development, many previous studies have shown that insufficient energy supply plays a key role in causing pollen abortion. This energy is provided by the mitochondria. Mitochondria are the main center of energy metabolism in eukaryotes, and their main functions are the tricarboxylic acid cycle and oxidative phosphorylation, synthesizing ATP to provide direct energy for life activities. Previous studies have shown that decreased sugar and biosynthetic fluxes in starch and altered redox are likely to result in altered gene expression, ultimately leading to pollen sterility (Datta et al., 2002). Therefore, we focused on developments related to energy metabolism and biological development. Seven genes were significantly downregulated and one gene was significantly upregulated in the enrichment pathway of “starch and sucrose metabolism” (Supplementary Table S8). This is consistent with our qRT-PCR results. By analyzing the KEGG pathway of genes with different levels of DNA methylation, we found that these genes were involved in a variety of metabolic pathways, which may have a complex regulatory effect on the development of anthers. Combined studies related to male sterility and bioinformatics analysis of these DMEGs, three genes associated with CMS were selected. *GOBAR_AA11600* was found to be homologous to the Arabidopsis HXK1 (hexokinase-1) gene, which belongs to the hexokinase family and may be involved in the phosphorylation of glucose during its export from the mitochondria to the cell membrane. As a sugar sensor, it can regulate the inhibition or activation of sugar-dependent genes, mediating the effects of sugar on plant growth and development and possibly regulating the execution of programmed cell death in plant cells, which is consistent with kong’s study (Kong et al., 2020), and we therefore speculated that the sterility of 07-113A may be related to aberrant PCD during pollen development. Of which *GOBAR_AA29872* is homologous to KORRIGAN 2 (KOR2), a superfamily protein of Arabidopsis hexahairpin glycosidases with hydrolase activity that hydrolyzes o-glycosyl compounds, and is involved in carbohydrate metabolism. *GOBAR_AA28360* is homologous to β-1,3-glucanase of Arabidopsis, an enzyme involved in pollen outer wall formation and possibly in pollen development around the microspore tetrads. Callus wall degradation during pollen development. Callus plays multiple roles in male gametophyte development, and it is hypothesized that *GOBAR_AA28360* expression is repressed in 07-113A, interfering with callus synthesis during male gametophyte development and leading to CMS. In addition, we also focused on the TCA cycle and the oxidative phosphorylation pathway. Four genes (*GOBAR_AA36705*, *GOBAR_AA26633*, *GOBAR_AA39007*, and *GOBAR_AA30876*) were significantly enriched and all of them were downregulated (Figure 7). Of which, *GOBAR_AA36705* belongs to the aconitase/IPM isomerase family, encoding an aconitase and a member of this family (ACO1—At35830) were shown to specifically bind the 5’ UTR of CSD2 *in vitro*. This protein is thought to be accumulated in the mitochondria and cytoplasm. This enzyme catalyzes the conversion of citric acid to isocitric acid *via* a cis-aconitate intermediate and may be involved in major metabolic pathways such as the TCA cycle. It affects CSD2 (At2g28190—a superoxide dismutase) transcript levels and may play a role in the oxidative stress response. The oxidative phosphorylation process occurs in the inner mitochondrial membrane and is closely linked to energy metabolism and the electron transport chain. We found that *GOBAR_AA30876* encodes V-type proton ATPase subunit a3, a multimeric enzyme that catalyzes proton transport through the cell membrane and is involved in vesicular nutrient storage (e.g., nitrate accumulation and storage) and tolerance to certain toxic ions (e.g., zinc ion sequestration in vesicles), which leads to the inference that 07-113A anther development. It is speculated that abortion may have occurred during anther development due to blockage of the electron transport chain in 07-113A.

### Possible Regulatory Mechanisms of DNA Methylation in Cotton CMS

Recent studies have shown that the expression of genes involved in energy metabolism and redox in cotton anthers is significantly regulated by DNA methylation. For example, DNA methylation has a significant effect on the expression of cotton anthers under high-temperature stress (Ma et al., 2018b; Zhang et al., 2019b). Our KEGG analysis revealed that super-DEGs, in 07-113B versus 07-113A, were significantly enriched in the “starch and sucrose metabolism” pathway (Figure 6A). Furthermore, all these hypermethylated genes were significantly upregulated in 07-113B (Figure 7). These results suggest that DNA hypermethylation may play a key role in anther development.

Based on the results of previous studies and our findings, we hypothesized that DNA methylation in 07-113A leads to the disturbance of energy metabolism and disrupts the homeostasis of mitochondria. It suppresses the expression of CMS-related genes, which eventually leads to microspore abortion. However, since gene expression is regulated at multiple levels and by multiple factors, the mechanisms of DNA methylation-mediated gene expression regulation are very complex. It remains to be further analyzed whether these differences in gene expression levels are caused by changes in the level of DNA methylation, or whether they are co-regulated by DNA methylation and other factors.

## Conclusion

A total of 847 DMEGs were identified between the CMS line 07-113A and its near-isogenetic maintainer line 07-113B by comprehensive methyl and transcriptome analysis. Studies have shown that 07-113A is more highly methylated than 07-113B. In DMEGs, hyper-DEGs were significantly enriched in the “starch and sucrose metabolism” pathway. We speculated that transcriptional inhibition was caused by hypermethylation of 07-113A, which resulted in the downregulation of gene expression. The change of reduced sugar flux in starch and its biosynthesis is likely to lead to altered gene expression and ultimately lead to pollen sterility. Five key DMGs that may be related to cotton CMS were identified, which are involved in carbohydrate and energy metabolism, male gametophyte development, and programmed cell death. Our results contribute to a better understanding of the possible role of DNA methylation in cotton CMS and will accelerate the study of the molecular mechanisms of CMS in cotton.

## Data Availability Statement

The datasets presented in this study can be found in online repositories. The names of the repository/repositories and accession number(s) can be found at: NCBI SRA BioProject, accession numbers: PRJNA786230 and PRJNA785387.

## Author Contributions

RZ and JY conceived, designed, and supervised the study. JY performed all experiments and drafted the manuscript. ML revised the manuscript. HL and XK participated in qRT-PCR. ML, YB, XZ, and XC participated in sample collection and assistance during the experiment. All authors contributed to the article and approved the submitted version.

## Funding

This work was supported by the National Natural Science Foundation of China (32060465), the Weng Hongwu Original Research Fund of Peking University of China (WHW201809), and Project funded by China Postdoctoral Science Foundation (2021MD703812).

## Conflict of Interest

YB is employed by Xinjiang Yida Textile Co., Ltd.

The remaining authors declare that the research was conducted in the absence of any commercial or financial relationships that could be construed as a potential conflict of interest.

## Publisher’s Note

All claims expressed in this article are solely those of the authors and do not necessarily represent those of their affiliated organizations, or those of the publisher, the editors and the reviewers. Any product that may be evaluated in this article, or claim that may be made by its manufacturer, is not guaranteed or endorsed by the publisher.

## Footnotes

^1^ https://www.ncbi.nlm.nih.gov/assembly/GCA_002928715.1/

^2^ http://www.geneontology.org/

^3^ http://www.kegg.jp/
