# Supplementary material for: Integrated Methylome and Transcriptome Analysis Widen the Knowledge of Cytoplasmic Male Sterility in Cotton (Gossypium barbadense L.)
Source: Front Plant Sci. 2022 Apr 27;13:770098. doi: 10.3389/fpls.2022.770098 (PMC9093596; doi:10.3389/fpls.2022.770098)
Supplement: Supplementary file 3 [file Table_3.DOCX]

**Supplementary Material**

**Supplementary table S3** 847 DEGs in DMR-associated genes（ZA:07-113A,ZB:07-113B）

| Gene ID | ZB FPKM | ZA FPKM | log2(ZA/ZB) | Qvalue(ZB-vs-ZA) | Pvalue(ZB-vs-ZA) |
| --- | --- | --- | --- | --- | --- |
| GOBAR_AA37964 | 0 | 1.843 | 5.518815837 | 9.75E-11 | 2.12E-12 |
| GOBAR_AA08371 | 0 | 4.14 | 4.805450237 | 1.09E-07 | 3.50E-09 |
| GOBAR_AA25984 | 0 | 2.063 | 4.460317876 | 2.40E-06 | 1.00E-07 |
| GOBAR_AA00180 | 0.01 | 1.25 | 4.38381639 | 8.93E-07 | 3.39E-08 |
| GOBAR_AA22117 | 27.553 | 600.196 | 4.175368442 | 2.21E-69 | 3.82E-72 |
| GOBAR_AA17575 | 0.006 | 0.716 | 3.994869881 | 2.15E-05 | 1.08E-06 |
| GOBAR_AA09464 | 0 | 2.736 | 3.73375814 | 0.000317967 | 2.29E-05 |
| GOBAR_AA14608 | 0.106 | 2.7 | 3.69900811 | 9.02E-07 | 3.43E-08 |
| GOBAR_AA27494 | 0 | 3.573 | 3.679854211 | 0.000305884 | 2.19E-05 |
| GOBAR_AA35206 | 0 | 0.383 | 3.424040732 | 0.00122571 | 0.000108766 |
| GOBAR_AA09793 | 0.033 | 0.94 | 3.375338312 | 0.00019616 | 1.31E-05 |
| GOBAR_AA36533 | 0.02 | 0.603 | 3.373825931 | 0.000263622 | 1.85E-05 |
| GOBAR_AA20233 | 0 | 0.286 | 3.339540242 | 0.001722284 | 0.000161857 |
| GOBAR_AA28336 | 0.263 | 4.613 | 3.287628685 | 8.93E-06 | 4.14E-07 |
| GOBAR_AA25907 | 0.05 | 0.893 | 3.215116666 | 0.000112808 | 6.94E-06 |
| GOBAR_AA20954 | 0.19 | 2.676 | 3.194249745 | 1.99E-06 | 8.11E-08 |
| GOBAR_AA03184 | 0.07 | 1.17 | 3.133520154 | 4.41E-05 | 2.43E-06 |
| GOBAR_AA05849 | 0.036 | 0.716 | 3.090720943 | 0.000498044 | 3.83E-05 |
| GOBAR_AA11474 | 0.073 | 1.1 | 3.040900084 | 0.000196 | 1.31E-05 |
| GOBAR_AA09839 | 1.126 | 12.093 | 3.00208738 | 3.19E-10 | 7.32E-12 |
| GOBAR_AA10017 | 0.083 | 1.076 | 2.98190794 | 9.72E-05 | 5.86E-06 |
| GOBAR_AA35205 | 0 | 0.273 | 2.971158793 | 0.007872635 | 0.000966617 |
| GOBAR_AA01435 | 0.22 | 2.856 | 2.93800453 | 0.000186105 | 1.23E-05 |
| GOBAR_AA40226 | 0.1 | 1.94 | 2.919925274 | 0.002687072 | 0.000269413 |
| GOBAR_AA36907 | 0.086 | 1.093 | 2.912345808 | 6.65E-05 | 3.84E-06 |
| GOBAR_AA37858 | 0 | 0.27 | 2.866387622 | 0.011754221 | 0.001579837 |
| GOBAR_AA07576 | 0.21 | 2.256 | 2.820328241 | 0.000122716 | 7.61E-06 |
| GOBAR_AA16537 | 0.84 | 8.773 | 2.75618209 | 7.43E-05 | 4.35E-06 |
| GOBAR_AA27160 | 0.606 | 5.883 | 2.72252255 | 1.25E-05 | 6.03E-07 |
| GOBAR_AA32399 | 8.916 | 71.006 | 2.677766014 | 7.43E-12 | 1.44E-13 |
| GOBAR_AA27788 | 8.06 | 111.336 | 2.660060659 | 0.007982065 | 0.000983693 |
| GOBAR_AA00012 | 0 | 0.253 | 2.64767932 | 0.023340005 | 0.003700664 |
| GOBAR_AA01007 | 0.726 | 6 | 2.620608179 | 0.000800047 | 6.64E-05 |
| GOBAR_AA27076 | 1.776 | 14.203 | 2.599386671 | 3.91E-05 | 2.12E-06 |
| GOBAR_AA40136 | 0 | 0.166 | 2.437416733 | 0.041953174 | 0.007710048 |
| GOBAR_AA29483 | 0.78 | 5.533 | 2.432850691 | 0.000408426 | 3.06E-05 |
| GOBAR_AA33915 | 0.01 | 0.31 | 2.414022708 | 0.040514655 | 0.007374093 |
| GOBAR_AA02965 | 0.893 | 6.3 | 2.404181509 | 2.42E-06 | 1.01E-07 |
| GOBAR_AA18610 | 0.123 | 0.836 | 2.330400501 | 0.002277106 | 0.000222587 |
| GOBAR_AA01568 | 1.096 | 6.656 | 2.285463016 | 3.14E-07 | 1.09E-08 |
| GOBAR_AA16690 | 0.67 | 4.24 | 2.280318182 | 1.09E-07 | 3.51E-09 |
| GOBAR_AA08909 | 0.103 | 0.746 | 2.244248955 | 0.003433729 | 0.000358657 |
| GOBAR_AA36954 | 2.09 | 10.793 | 2.20620869 | 1.01E-05 | 4.75E-07 |
| GOBAR_AA06692 | 0.06 | 0.463 | 2.202333991 | 0.016264756 | 0.002359444 |
| GOBAR_AA18329 | 0.616 | 3.57 | 2.173071347 | 4.30E-05 | 2.36E-06 |
| GOBAR_AA37329 | 24.76 | 137.346 | 2.144200082 | 8.50E-11 | 1.84E-12 |
| GOBAR_AA33473 | 0.186 | 1.116 | 2.136059172 | 0.000555365 | 4.34E-05 |
| GOBAR_AA20804 | 0.17 | 1.19 | 2.127665999 | 0.036381965 | 0.006433762 |
| GOBAR_AA40391 | 0.093 | 0.573 | 2.111490706 | 0.003644204 | 0.000384432 |
| GOBAR_AA31127 | 1.38 | 9.513 | 2.101147659 | 0.017644741 | 0.002608106 |
| GOBAR_AA04804 | 0.73 | 5.016 | 2.096503585 | 0.018276895 | 0.002730527 |
| GOBAR_AA38190 | 0.306 | 2.446 | 2.084873243 | 0.04610672 | 0.00868355 |
| GOBAR_AA27999 | 0.536 | 2.893 | 2.08041683 | 0.00050251 | 3.88E-05 |
| GOBAR_AA29776 | 1.206 | 8.286 | 2.0670993 | 8.01E-08 | 2.52E-09 |
| GOBAR_AA15039 | 0.973 | 5.573 | 2.037727788 | 0.002679054 | 0.000268455 |
| GOBAR_AA10808 | 3.066 | 15.25 | 2.016865215 | 0.000139185 | 8.78E-06 |
| GOBAR_AA39182 | 0.266 | 1.536 | 1.978291533 | 0.018391859 | 0.002751952 |
| GOBAR_AA40302 | 0.136 | 0.826 | 1.961259179 | 0.026330961 | 0.004292025 |
| GOBAR_AA10688 | 18.263 | 86.396 | 1.953963038 | 8.56E-09 | 2.38E-10 |
| GOBAR_AA37399 | 0.09 | 0.516 | 1.948217093 | 0.022823079 | 0.003596288 |
| GOBAR_AA17752 | 0.113 | 0.64 | 1.926903663 | 0.027064001 | 0.004453728 |
| GOBAR_AA09417 | 0.643 | 2.786 | 1.92514762 | 0.000498829 | 3.84E-05 |
| GOBAR_AA16012 | 1.513 | 7.473 | 1.893800119 | 0.010506433 | 0.001377226 |
| GOBAR_AA28454 | 0.13 | 0.766 | 1.879653004 | 0.044972819 | 0.008414135 |
| GOBAR_AA29037 | 7.843 | 36.603 | 1.866873118 | 4.81E-05 | 2.68E-06 |
| GOBAR_AA16458 | 1.476 | 6.153 | 1.797784714 | 7.04E-08 | 2.20E-09 |
| GOBAR_AA33977 | 5.43 | 24.33 | 1.793059593 | 0.006166262 | 0.000718706 |
| GOBAR_AA16558 | 0.526 | 2.383 | 1.784815162 | 0.005023183 | 0.000562404 |
| GOBAR_AA38726 | 1.206 | 5.296 | 1.781444194 | 0.006511092 | 0.000763975 |
| GOBAR_AA24735 | 1.56 | 6.68 | 1.753375375 | 0.000251038 | 1.74E-05 |
| GOBAR_AA35935 | 0.13 | 0.613 | 1.747044196 | 0.049675211 | 0.009569427 |
| GOBAR_AA22364 | 2.026 | 8.716 | 1.71335257 | 0.010597467 | 0.00139069 |
| GOBAR_AA20558 | 4.743 | 18.56 | 1.712868344 | 2.88E-10 | 6.55E-12 |
| GOBAR_AA14676 | 13.153 | 51.003 | 1.703081208 | 2.89E-07 | 9.97E-09 |
| GOBAR_AA33790 | 0.08 | 0.326 | 1.690830593 | 0.033152917 | 0.00574399 |
| GOBAR_AA23951 | 0.533 | 2.206 | 1.68702561 | 0.00255994 | 0.00025504 |
| GOBAR_AA18614 | 0.606 | 2.386 | 1.678408447 | 0.000563625 | 4.42E-05 |
| GOBAR_AA21232 | 2.146 | 8.646 | 1.67687231 | 0.000839863 | 7.02E-05 |
| GOBAR_AA08006 | 1.893 | 7.446 | 1.674148528 | 0.000453384 | 3.44E-05 |
| GOBAR_AA06049 | 39.163 | 147.856 | 1.673275923 | 1.71E-22 | 1.41E-24 |
| GOBAR_AA01728 | 2.57 | 9.986 | 1.657639866 | 1.14E-06 | 4.43E-08 |
| GOBAR_AA23346 | 0.466 | 1.86 | 1.646613051 | 0.006686887 | 0.000790459 |
| GOBAR_AA16977 | 2.416 | 9.353 | 1.632574809 | 0.001885819 | 0.000178891 |
| GOBAR_AA37753 | 0.73 | 2.916 | 1.627465301 | 0.013473404 | 0.001874344 |
| GOBAR_AA33954 | 6.833 | 24.633 | 1.605225638 | 8.07E-05 | 4.76E-06 |
| GOBAR_AA22924 | 0.313 | 1.263 | 1.604590737 | 0.019558713 | 0.00297344 |
| GOBAR_AA35657 | 5.153 | 19.086 | 1.604168525 | 2.05E-10 | 4.59E-12 |
| GOBAR_AA32263 | 1.026 | 4.146 | 1.601970584 | 0.046078016 | 0.008676506 |
| GOBAR_AA11537 | 1.49 | 5.456 | 1.594841783 | 0.001532735 | 0.000141191 |
| GOBAR_AA21224 | 2.506 | 8.803 | 1.542072285 | 1.16E-07 | 3.78E-09 |
| GOBAR_AA02684 | 0.71 | 2.62 | 1.538220709 | 0.03269129 | 0.005637569 |
| GOBAR_AA01307 | 0.436 | 1.576 | 1.511961612 | 0.001306638 | 0.000117042 |
| GOBAR_AA24237 | 0.616 | 2.3 | 1.504367768 | 0.023576722 | 0.003745603 |
| GOBAR_AA37171 | 12.786 | 43.686 | 1.496556505 | 1.45E-05 | 7.10E-07 |
| GOBAR_AA38102 | 1.076 | 3.503 | 1.457089318 | 0.001497152 | 0.000137293 |
| GOBAR_AA14640 | 7.37 | 22.883 | 1.372670253 | 2.07E-10 | 4.65E-12 |
| GOBAR_AA11323 | 2.58 | 8.276 | 1.364981697 | 0.010085479 | 0.001313889 |
| GOBAR_AA36162 | 5.256 | 15.986 | 1.362313527 | 7.33E-09 | 2.02E-10 |
| GOBAR_AA35126 | 0.433 | 1.353 | 1.360127134 | 0.009898065 | 0.001283469 |
| GOBAR_AA19948 | 11.7 | 26.07 | 1.350797871 | 0.006020018 | 0.000697313 |
| GOBAR_AA34881 | 9.25 | 27.926 | 1.346199841 | 2.44E-06 | 1.02E-07 |
| GOBAR_AA35264 | 3.893 | 12.026 | 1.338397048 | 0.003728446 | 0.000395306 |
| GOBAR_AA01934 | 2.42 | 6.326 | 1.333813036 | 0.045761785 | 0.00860008 |
| GOBAR_AA30676 | 4.166 | 12.43 | 1.320548674 | 2.74E-07 | 9.41E-09 |
| GOBAR_AA38637 | 0.796 | 2.38 | 1.311756919 | 0.01263484 | 0.00172776 |
| GOBAR_AA00449 | 1.3 | 3.893 | 1.295190693 | 0.018234185 | 0.002720986 |
| GOBAR_AA40129 | 4.03 | 11.74 | 1.290842731 | 0.000125553 | 7.82E-06 |
| GOBAR_AA29569 | 30.31 | 87.273 | 1.287083856 | 1.12E-05 | 5.35E-07 |
| GOBAR_AA40378 | 0.29 | 0.88 | 1.286416996 | 0.023321905 | 0.003696447 |
| GOBAR_AA06516 | 39.673 | 116.11 | 1.283830866 | 3.22E-07 | 1.12E-08 |
| GOBAR_AA39861 | 14.403 | 41.31 | 1.281596396 | 9.79E-05 | 5.91E-06 |
| GOBAR_AA34025 | 2.856 | 8.23 | 1.279572451 | 0.000912815 | 7.75E-05 |
| GOBAR_AA37962 | 4.243 | 12.373 | 1.276116624 | 1.44E-05 | 7.06E-07 |
| GOBAR_AA29215 | 1.933 | 5.75 | 1.273665406 | 0.017991569 | 0.002675427 |
| GOBAR_AA15597 | 1.486 | 4.46 | 1.270309401 | 0.010261724 | 0.001341888 |
| GOBAR_AA38442 | 0.813 | 2.326 | 1.26566052 | 0.001111127 | 9.73E-05 |
| GOBAR_AA34516 | 10.266 | 30.253 | 1.258397794 | 0.002140959 | 0.00020699 |
| GOBAR_AA06355 | 1.883 | 5.613 | 1.255366831 | 0.030858533 | 0.005251985 |
| GOBAR_AA34246 | 2.51 | 7.196 | 1.252337828 | 0.000103022 | 6.27E-06 |
| GOBAR_AA22149 | 16.156 | 46.203 | 1.248345272 | 0.000519825 | 4.02E-05 |
| GOBAR_AA33754 | 1.136 | 3.253 | 1.243091605 | 0.01015353 | 0.001324514 |
| GOBAR_AA29865 | 10.346 | 29.456 | 1.241539908 | 0.000852096 | 7.16E-05 |
| GOBAR_AA14260 | 0.65 | 1.946 | 1.24084143 | 0.04836607 | 0.009233408 |
| GOBAR_AA18473 | 3.543 | 9.313 | 1.237718773 | 0.005526244 | 0.000633413 |
| GOBAR_AA23527 | 7.833 | 22.053 | 1.229450429 | 0.017867516 | 0.002651818 |
| GOBAR_AA31391 | 25.096 | 51.836 | 1.227511855 | 1.26E-05 | 6.09E-07 |
| GOBAR_AA23939 | 41.273 | 113.606 | 1.209756778 | 1.57E-10 | 3.47E-12 |
| GOBAR_AA30306 | 6.29 | 17.19 | 1.208872877 | 1.21E-10 | 2.65E-12 |
| GOBAR_AA03061 | 1.896 | 5.273 | 1.192763182 | 0.000600866 | 4.75E-05 |
| GOBAR_AA36277 | 1.653 | 4.8 | 1.191137268 | 0.033131295 | 0.00573833 |
| GOBAR_AA18336 | 6.36 | 17.38 | 1.189942396 | 5.90E-06 | 2.63E-07 |
| GOBAR_AA06047 | 1.976 | 5.426 | 1.189337105 | 0.000561233 | 4.40E-05 |
| GOBAR_AA39544 | 1.633 | 4.62 | 1.173910136 | 0.044437303 | 0.008288271 |
| GOBAR_AA20123 | 0.78 | 2.1 | 1.162140326 | 0.023607991 | 0.003753239 |
| GOBAR_AA34598 | 1.053 | 2.863 | 1.161981405 | 0.014734339 | 0.002094873 |
| GOBAR_AA30941 | 0.79 | 2.16 | 1.155485578 | 0.020226334 | 0.003100059 |
| GOBAR_AA30274 | 3.936 | 10.303 | 1.151496869 | 0.00108178 | 9.42E-05 |
| GOBAR_AA15515 | 2.226 | 5.896 | 1.149896199 | 0.001903955 | 0.000180831 |
| GOBAR_AA23209 | 17.63 | 46.73 | 1.148532257 | 0.001038409 | 8.97E-05 |
| GOBAR_AA16459 | 1.123 | 2.956 | 1.143172207 | 0.018358804 | 0.002744428 |
| GOBAR_AA12762 | 2.7 | 7.173 | 1.14311987 | 0.000964676 | 8.26E-05 |
| GOBAR_AA12619 | 3.15 | 8.343 | 1.142140579 | 0.035872925 | 0.006324156 |
| GOBAR_AA26422 | 9.086 | 23.553 | 1.130903794 | 0.003115226 | 0.00031954 |
| GOBAR_AA06761 | 1.283 | 3.36 | 1.127695587 | 0.006628468 | 0.000781385 |
| GOBAR_AA13934 | 0.673 | 1.933 | 1.1230564 | 0.036053783 | 0.006360104 |
| GOBAR_AA34419 | 10.076 | 26.186 | 1.120594421 | 8.43E-05 | 5.00E-06 |
| GOBAR_AA06524 | 1.046 | 2.7 | 1.116503737 | 0.004035351 | 0.000434466 |
| GOBAR_AA06740 | 19.833 | 51.64 | 1.111814606 | 0.000117652 | 7.27E-06 |
| GOBAR_AA16498 | 16.73 | 44.393 | 1.100898718 | 0.005067526 | 0.000568979 |
| GOBAR_AA02709 | 5.833 | 14.666 | 1.096725302 | 0.000406455 | 3.04E-05 |
| GOBAR_AA14559 | 1.776 | 4.33 | 1.095669419 | 1.44E-05 | 7.02E-07 |
| GOBAR_AA39720 | 26.386 | 66.653 | 1.095597824 | 0.000615651 | 4.89E-05 |
| GOBAR_AA27644 | 1.563 | 4.396 | 1.09091245 | 0.000525164 | 4.07E-05 |
| GOBAR_AA00229 | 46.81 | 122.376 | 1.090698295 | 0.000166605 | 1.08E-05 |
| GOBAR_AA20313 | 8.51 | 20.943 | 1.086742032 | 0.009378178 | 0.001200886 |
| GOBAR_AA03907 | 7.116 | 16.726 | 1.086740736 | 0.000481897 | 3.70E-05 |
| GOBAR_AA26037 | 6.52 | 16.56 | 1.083032138 | 0.006162649 | 0.000717928 |
| GOBAR_AA31929 | 6.393 | 16.726 | 1.082879171 | 0.001038409 | 8.97E-05 |
| GOBAR_AA02806 | 6.393 | 15.94 | 1.082321259 | 1.33E-05 | 6.46E-07 |
| GOBAR_AA23969 | 4.936 | 12.8 | 1.082137121 | 4.96E-05 | 2.77E-06 |
| GOBAR_AA16698 | 5.16 | 15.216 | 1.070714026 | 0.000126386 | 7.87E-06 |
| GOBAR_AA19743 | 5.066 | 12.656 | 1.070053568 | 3.76E-05 | 2.03E-06 |
| GOBAR_AA15108 | 22.43 | 55.2 | 1.064385652 | 0.00443404 | 0.000485427 |
| GOBAR_AA03562 | 5.7 | 14.376 | 1.061718662 | 0.041318672 | 0.007560986 |
| GOBAR_AA28753 | 75.483 | 182.333 | 1.056085339 | 1.43E-05 | 6.95E-07 |
| GOBAR_AA07195 | 40.726 | 99.423 | 1.052516296 | 5.75E-08 | 1.77E-09 |
| GOBAR_AA14051 | 30.726 | 73.406 | 1.051686238 | 0.00010357 | 6.31E-06 |
| GOBAR_AA34517 | 1.246 | 3.096 | 1.047735328 | 0.018861617 | 0.002834228 |
| GOBAR_AA02699 | 5.21 | 12.686 | 1.043365648 | 1.68E-05 | 8.30E-07 |
| GOBAR_AA11392 | 3.68 | 10.66 | 1.041546464 | 0.00200407 | 0.000191556 |
| GOBAR_AA02628 | 88.42 | 213.94 | 1.040426783 | 5.78E-08 | 1.78E-09 |
| GOBAR_AA08878 | 24.496 | 59.44 | 1.036298675 | 1.59E-05 | 7.83E-07 |
| GOBAR_AA16999 | 22.94 | 55.84 | 1.035744391 | 0.02419853 | 0.003867704 |
| GOBAR_AA12986 | 6.91 | 17.296 | 1.033405805 | 0.006899289 | 0.000823275 |
| GOBAR_AA26546 | 2.993 | 7.416 | 1.032526048 | 0.018919107 | 0.002845052 |
| GOBAR_AA08022 | 5.403 | 10.856 | 1.030480363 | 0.000127256 | 7.94E-06 |
| GOBAR_AA07335 | 420.376 | 956.006 | 1.024678426 | 0.000769395 | 6.33E-05 |
| GOBAR_AA25540 | 9.736 | 23.346 | 1.022804013 | 0.037613761 | 0.00672982 |
| GOBAR_AA10704 | 31.35 | 72.026 | 1.022128018 | 0.045610149 | 0.008564996 |
| GOBAR_AA11709 | 12.596 | 34.076 | 1.016116255 | 0.010044362 | 0.001306791 |
| GOBAR_AA20393 | 2.776 | 6.636 | 1.012559553 | 0.009041158 | 0.001149112 |
| GOBAR_AA27930 | 10.893 | 25.426 | 1.010358491 | 3.14E-06 | 1.34E-07 |
| GOBAR_AA08214 | 4.23 | 10.103 | 1.009268082 | 6.54E-05 | 3.77E-06 |
| GOBAR_AA33669 | 42.073 | 100.543 | 1.005255739 | 0.000330058 | 2.39E-05 |
| GOBAR_AA21999 | 8.703 | 20.443 | 0.990551547 | 0.000215172 | 1.46E-05 |
| GOBAR_AA34154 | 9.973 | 23.963 | 0.985602916 | 0.003836803 | 0.000409404 |
| GOBAR_AA13859 | 39.06 | 90.856 | 0.98014425 | 1.52E-05 | 7.47E-07 |
| GOBAR_AA30755 | 3.416 | 8.156 | 0.97890934 | 0.013473404 | 0.001873994 |
| GOBAR_AA20757 | 4.093 | 9.703 | 0.972999271 | 0.000702421 | 5.70E-05 |
| GOBAR_AA29570 | 24.653 | 55.376 | 0.972804552 | 0.036385606 | 0.006436507 |
| GOBAR_AA34378 | 4.053 | 9.18 | 0.96914879 | 0.000231728 | 1.59E-05 |
| GOBAR_AA19518 | 3.153 | 7.253 | 0.968935599 | 0.011221558 | 0.001493982 |
| GOBAR_AA11168 | 15.85 | 36.406 | 0.96820498 | 0.008868039 | 0.001118655 |
| GOBAR_AA03855 | 6.27 | 14.19 | 0.966835075 | 0.000873103 | 7.37E-05 |
| GOBAR_AA24272 | 18.286 | 41.733 | 0.952309871 | 1.02E-06 | 3.91E-08 |
| GOBAR_AA25791 | 6.256 | 14.206 | 0.950606864 | 1.04E-05 | 4.90E-07 |
| GOBAR_AA26364 | 25.06 | 45.8 | 0.950269589 | 0.005092897 | 0.000573298 |
| GOBAR_AA08940 | 12.82 | 29.006 | 0.946478273 | 0.003728446 | 0.000395365 |
| GOBAR_AA32479 | 24.876 | 58.44 | 0.942006239 | 0.022667982 | 0.003567266 |
| GOBAR_AA08368 | 3.976 | 9.043 | 0.940143653 | 0.000204272 | 1.37E-05 |
| GOBAR_AA26825 | 5.05 | 11.31 | 0.936236904 | 0.003577458 | 0.000376047 |
| GOBAR_AA02697 | 34.76 | 78.013 | 0.933767656 | 7.33E-06 | 3.33E-07 |
| GOBAR_AA26118 | 2.166 | 4.913 | 0.930797551 | 0.048828562 | 0.009360049 |
| GOBAR_AA17482 | 3.233 | 7.763 | 0.930120521 | 0.041553234 | 0.007609911 |
| GOBAR_AA00789 | 4.506 | 10.276 | 0.928199316 | 0.009600344 | 0.001236741 |
| GOBAR_AA34852 | 6.41 | 14.546 | 0.927903141 | 0.013640709 | 0.001902741 |
| GOBAR_AA38890 | 17.65 | 39.23 | 0.925872338 | 0.000627142 | 4.99E-05 |
| GOBAR_AA09673 | 4.063 | 9.056 | 0.921886523 | 0.004415435 | 0.000483136 |
| GOBAR_AA01087 | 9.876 | 21.32 | 0.920947029 | 9.92E-06 | 4.67E-07 |
| GOBAR_AA31609 | 58.506 | 123.313 | 0.912692795 | 6.56E-06 | 2.95E-07 |
| GOBAR_AA13674 | 11.476 | 25.486 | 0.91174142 | 0.000259208 | 1.81E-05 |
| GOBAR_AA22472 | 1.536 | 3.506 | 0.907758218 | 0.017521715 | 0.0025843 |
| GOBAR_AA27157 | 4.42 | 9.7 | 0.906879292 | 0.005543279 | 0.000635526 |
| GOBAR_AA02267 | 7.33 | 16.013 | 0.903436724 | 0.000365662 | 2.69E-05 |
| GOBAR_AA03213 | 17.34 | 39.593 | 0.895088864 | 0.000690179 | 5.58E-05 |
| GOBAR_AA04919 | 10.863 | 23.763 | 0.892330215 | 0.001136611 | 9.99E-05 |
| GOBAR_AA08402 | 16.153 | 34.743 | 0.891393137 | 0.000850586 | 7.14E-05 |
| GOBAR_AA28344 | 5.936 | 13.273 | 0.886493132 | 0.000496501 | 3.82E-05 |
| GOBAR_AA17188 | 5.546 | 12.033 | 0.882617892 | 0.001136611 | 9.99E-05 |
| GOBAR_AA39746 | 9.016 | 17.02 | 0.880255447 | 3.40E-05 | 1.81E-06 |
| GOBAR_AA12266 | 29.823 | 64.46 | 0.88007132 | 0.007316605 | 0.000881949 |
| GOBAR_AA30234 | 12.766 | 27.983 | 0.879731952 | 0.000156552 | 1.00E-05 |
| GOBAR_AA11984 | 16.13 | 34.636 | 0.877675737 | 0.000461217 | 3.51E-05 |
| GOBAR_AA01088 | 11.793 | 25.753 | 0.873596424 | 0.006059332 | 0.000703617 |
| GOBAR_AA29256 | 43.743 | 94.693 | 0.872676834 | 1.98E-06 | 8.08E-08 |
| GOBAR_AA08434 | 9.963 | 21.373 | 0.869331371 | 0.002197753 | 0.000213179 |
| GOBAR_AA07021 | 1.563 | 3.25 | 0.868553147 | 0.008574762 | 0.001073982 |
| GOBAR_AA33351 | 29.74 | 64.98 | 0.859141553 | 0.001061738 | 9.23E-05 |
| GOBAR_AA00048 | 6.103 | 13.15 | 0.85831318 | 0.006072855 | 0.000705714 |
| GOBAR_AA35519 | 12.59 | 26.746 | 0.857756344 | 0.00021499 | 1.45E-05 |
| GOBAR_AA32900 | 3.356 | 7.17 | 0.854224955 | 0.043032142 | 0.007961549 |
| GOBAR_AA20036 | 10.606 | 23.04 | 0.853792298 | 0.022276635 | 0.003491523 |
| GOBAR_AA30436 | 27.496 | 58.493 | 0.853231298 | 0.000213685 | 1.44E-05 |
| GOBAR_AA12693 | 2.69 | 5.773 | 0.849451615 | 0.026427954 | 0.004313942 |
| GOBAR_AA34708 | 7.403 | 14.533 | 0.84725195 | 0.003767142 | 0.000400447 |
| GOBAR_AA40200 | 6.526 | 13.833 | 0.844598592 | 0.005172729 | 0.000584825 |
| GOBAR_AA21104 | 1.08 | 2.273 | 0.844154461 | 0.009006822 | 0.001142926 |
| GOBAR_AA03024 | 14.016 | 30.34 | 0.843957851 | 0.000365662 | 2.69E-05 |
| GOBAR_AA01872 | 5.376 | 11.723 | 0.843477911 | 0.003693704 | 0.000390451 |
| GOBAR_AA21483 | 9.97 | 21.02 | 0.839050751 | 0.003427113 | 0.000357868 |
| GOBAR_AA12378 | 2.013 | 4.6 | 0.838842184 | 0.011076288 | 0.001468882 |
| GOBAR_AA24855 | 29.933 | 64.33 | 0.833891826 | 0.000592796 | 4.67E-05 |
| GOBAR_AA38653 | 25.83 | 54.35 | 0.831680413 | 0.007205693 | 0.000866049 |
| GOBAR_AA21872 | 4.043 | 8.803 | 0.824783443 | 0.000260961 | 1.83E-05 |
| GOBAR_AA19835 | 13.06 | 27.246 | 0.824165145 | 0.013318996 | 0.001843353 |
| GOBAR_AA15330 | 6.23 | 12.25 | 0.823342034 | 0.021928513 | 0.003417629 |
| GOBAR_AA27069 | 12.323 | 26.003 | 0.82187629 | 0.010843624 | 0.001432826 |
| GOBAR_AA03237 | 3.07 | 6.66 | 0.81375357 | 0.034184104 | 0.005947337 |
| GOBAR_AA11275 | 32.663 | 67.783 | 0.811487537 | 0.008827009 | 0.001112715 |
| GOBAR_AA02507 | 5.253 | 11.206 | 0.809513417 | 0.001939678 | 0.000184729 |
| GOBAR_AA39857 | 56.283 | 118.03 | 0.806005625 | 0.002115908 | 0.000204201 |
| GOBAR_AA27905 | 4.216 | 8.61 | 0.805298959 | 0.000504474 | 3.89E-05 |
| GOBAR_AA16836 | 20.77 | 44.46 | 0.804488645 | 0.000146013 | 9.28E-06 |
| GOBAR_AA10595 | 5.16 | 10.623 | 0.802850401 | 0.007374267 | 0.000890817 |
| GOBAR_AA27552 | 44.013 | 88.296 | 0.797667279 | 0.00015901 | 1.02E-05 |
| GOBAR_AA17379 | 15.986 | 32.126 | 0.797161377 | 0.001351747 | 0.000121551 |
| GOBAR_AA22517 | 14.04 | 27.746 | 0.794144244 | 0.007295948 | 0.000879248 |
| GOBAR_AA26374 | 10.403 | 21.53 | 0.792318799 | 0.003725449 | 0.000394831 |
| GOBAR_AA40034 | 4.683 | 9.856 | 0.791171879 | 0.000154229 | 9.86E-06 |
| GOBAR_AA26755 | 20.59 | 42.11 | 0.789823104 | 0.006433466 | 0.000753194 |
| GOBAR_AA19571 | 4.533 | 9.97 | 0.789721998 | 0.002346599 | 0.000230329 |
| GOBAR_AA21940 | 5.263 | 11.03 | 0.787164127 | 0.010332867 | 0.001352087 |
| GOBAR_AA06042 | 8.79 | 17.493 | 0.78431926 | 0.005329038 | 0.000606808 |
| GOBAR_AA03008 | 3.466 | 7.256 | 0.780422146 | 0.00163811 | 0.000152527 |
| GOBAR_AA17865 | 6.013 | 11.906 | 0.774963628 | 0.000175698 | 1.15E-05 |
| GOBAR_AA09931 | 8.333 | 16.933 | 0.774316653 | 0.011424061 | 0.001525605 |
| GOBAR_AA12585 | 109.53 | 218.246 | 0.773443876 | 5.38E-06 | 2.37E-07 |
| GOBAR_AA33383 | 8.43 | 16.866 | 0.771842667 | 0.036072675 | 0.006366223 |
| GOBAR_AA31619 | 8.583 | 17.303 | 0.768645932 | 0.047237962 | 0.008949819 |
| GOBAR_AA20174 | 130.803 | 254.743 | 0.763201728 | 0.000232259 | 1.59E-05 |
| GOBAR_AA05660 | 4.053 | 8.233 | 0.760655301 | 0.016965023 | 0.002486511 |
| GOBAR_AA11823 | 4.986 | 9.946 | 0.760406678 | 0.008917641 | 0.001127231 |
| GOBAR_AA38062 | 7.803 | 15.95 | 0.756222438 | 0.008627375 | 0.001082814 |
| GOBAR_AA27774 | 10.106 | 20.033 | 0.755029685 | 0.009758531 | 0.001260584 |
| GOBAR_AA14719 | 6 | 12.13 | 0.752501897 | 0.039770768 | 0.007213399 |
| GOBAR_AA35079 | 24.806 | 49.503 | 0.749254327 | 0.002220771 | 0.000215925 |
| GOBAR_AA04632 | 12.006 | 24.826 | 0.746836706 | 0.002178751 | 0.00021121 |
| GOBAR_AA22042 | 22.52 | 44.606 | 0.744018044 | 0.001199976 | 0.000106258 |
| GOBAR_AA21046 | 8.59 | 17.05 | 0.742361662 | 0.039365225 | 0.007117102 |
| GOBAR_AA17519 | 3.75 | 7.393 | 0.741085407 | 0.03064604 | 0.005207853 |
| GOBAR_AA03230 | 39.886 | 77.936 | 0.740488954 | 0.000899581 | 7.62E-05 |
| GOBAR_AA01076 | 8.183 | 15.93 | 0.739289543 | 0.019289424 | 0.0029247 |
| GOBAR_AA23871 | 5.806 | 11.953 | 0.737491131 | 0.028246015 | 0.004699646 |
| GOBAR_AA11063 | 16.653 | 32.57 | 0.734682349 | 0.00645277 | 0.000756014 |
| GOBAR_AA38914 | 21.966 | 42.83 | 0.734004568 | 0.003031189 | 0.000309782 |
| GOBAR_AA01930 | 7.013 | 14.686 | 0.728517509 | 0.045837735 | 0.008622298 |
| GOBAR_AA21656 | 9.823 | 19.166 | 0.727984611 | 0.00010827 | 6.62E-06 |
| GOBAR_AA26827 | 10.32 | 20.226 | 0.727587104 | 0.047714703 | 0.009066204 |
| GOBAR_AA34512 | 26.056 | 50.846 | 0.726707261 | 0.004073683 | 0.000439387 |
| GOBAR_AA12376 | 4.066 | 8.05 | 0.72612436 | 0.043866206 | 0.008158945 |
| GOBAR_AA05687 | 6.4 | 12.08 | 0.721820659 | 0.032276906 | 0.005547462 |
| GOBAR_AA40176 | 8.076 | 15.593 | 0.720465812 | 0.006402642 | 0.000749031 |
| GOBAR_AA23414 | 12.516 | 24.196 | 0.717700009 | 0.000204001 | 1.37E-05 |
| GOBAR_AA38980 | 9.756 | 18.773 | 0.71452186 | 0.000258341 | 1.81E-05 |
| GOBAR_AA24602 | 42.843 | 82.496 | 0.713016497 | 9.05E-06 | 4.21E-07 |
| GOBAR_AA00671 | 23.953 | 45.706 | 0.710708524 | 2.37E-05 | 1.21E-06 |
| GOBAR_AA09696 | 3.613 | 6.936 | 0.705910152 | 0.014162184 | 0.00199389 |
| GOBAR_AA02217 | 3.456 | 6.623 | 0.700004822 | 0.013755032 | 0.001921866 |
| GOBAR_AA37724 | 42.976 | 81.25 | 0.69862848 | 3.69E-06 | 1.59E-07 |
| GOBAR_AA38284 | 132.476 | 250.45 | 0.696024857 | 0.022401229 | 0.00351558 |
| GOBAR_AA10732 | 9.523 | 18.07 | 0.694671321 | 0.002716204 | 0.000273093 |
| GOBAR_AA28518 | 28.693 | 55.43 | 0.693870829 | 0.025536432 | 0.004130145 |
| GOBAR_AA19030 | 146.053 | 275.516 | 0.687931516 | 2.99E-05 | 1.57E-06 |
| GOBAR_AA10155 | 31.773 | 60.32 | 0.686363605 | 0.037267099 | 0.006643036 |
| GOBAR_AA32819 | 4.763 | 9.046 | 0.685436668 | 0.022241955 | 0.003484802 |
| GOBAR_AA26663 | 34.566 | 65.346 | 0.682270263 | 0.000102467 | 6.22E-06 |
| GOBAR_AA24575 | 28.29 | 53.076 | 0.681119926 | 0.002333362 | 0.000228962 |
| GOBAR_AA17333 | 6.376 | 11.963 | 0.675330295 | 0.037120499 | 0.006608326 |
| GOBAR_AA25940 | 9.636 | 17.633 | 0.674501926 | 0.002320276 | 0.000227477 |
| GOBAR_AA01058 | 10.233 | 19.143 | 0.67363679 | 0.01418113 | 0.001997787 |
| GOBAR_AA14757 | 16.79 | 31.35 | 0.671501753 | 0.000397751 | 2.97E-05 |
| GOBAR_AA26013 | 26.863 | 50.426 | 0.670619363 | 8.39E-05 | 4.97E-06 |
| GOBAR_AA34291 | 9.093 | 17.04 | 0.668946221 | 9.66E-05 | 5.82E-06 |
| GOBAR_AA03160 | 10.036 | 18.78 | 0.666847585 | 0.028168357 | 0.004684284 |
| GOBAR_AA24818 | 42.373 | 76.59 | 0.664078589 | 0.001932444 | 0.000183928 |
| GOBAR_AA27180 | 7.043 | 12.93 | 0.657439277 | 0.008233237 | 0.001021217 |
| GOBAR_AA27553 | 12.07 | 22.246 | 0.656204021 | 0.008423109 | 0.001050121 |
| GOBAR_AA10715 | 15.43 | 28.173 | 0.655896649 | 0.013368704 | 0.0018536 |
| GOBAR_AA19321 | 20.853 | 39.146 | 0.655151541 | 0.003792614 | 0.000403593 |
| GOBAR_AA06338 | 8.346 | 15.553 | 0.650005247 | 0.03456617 | 0.006027787 |
| GOBAR_AA37634 | 20.59 | 37.963 | 0.647855531 | 0.002225898 | 0.000216874 |
| GOBAR_AA38946 | 11.853 | 21.54 | 0.645109911 | 0.005594261 | 0.000642341 |
| GOBAR_AA24812 | 13.036 | 25.256 | 0.643880463 | 0.040569987 | 0.007387654 |
| GOBAR_AA24721 | 7.333 | 13.61 | 0.642512971 | 0.034966893 | 0.006116858 |
| GOBAR_AA12495 | 27.78 | 52.413 | 0.642263247 | 0.000184267 | 1.22E-05 |
| GOBAR_AA24313 | 12.196 | 23.436 | 0.642243045 | 0.043952004 | 0.008179981 |
| GOBAR_AA08721 | 9.74 | 17.856 | 0.642216437 | 0.007982065 | 0.000984068 |
| GOBAR_AA00889 | 169.916 | 314.13 | 0.638785211 | 0.001045022 | 9.05E-05 |
| GOBAR_AA12876 | 6.853 | 12.806 | 0.638513544 | 0.036184307 | 0.006391491 |
| GOBAR_AA22774 | 19.186 | 35.26 | 0.636911306 | 0.021061902 | 0.003253864 |
| GOBAR_AA02371 | 13.3 | 24.423 | 0.635132561 | 0.027572646 | 0.004559733 |
| GOBAR_AA14851 | 23.636 | 46.873 | 0.633980992 | 0.013350095 | 0.001849477 |
| GOBAR_AA25671 | 7.883 | 15.156 | 0.627004511 | 0.004568569 | 0.000502003 |
| GOBAR_AA27334 | 17.363 | 31.64 | 0.625172057 | 0.019819104 | 0.003018751 |
| GOBAR_AA12307 | 7.796 | 14.196 | 0.62406126 | 0.003970667 | 0.000426179 |
| GOBAR_AA30446 | 2.146 | 3.866 | 0.622283293 | 0.036643926 | 0.006493847 |
| GOBAR_AA35814 | 8.106 | 14.563 | 0.619818166 | 0.03173981 | 0.005434064 |
| GOBAR_AA32299 | 8.42 | 14.456 | 0.617770866 | 0.021214977 | 0.003282227 |
| GOBAR_AA07060 | 7.473 | 13.436 | 0.615457293 | 0.004601585 | 0.0005066 |
| GOBAR_AA22724 | 7.33 | 13.136 | 0.610564735 | 0.043362093 | 0.008033868 |
| GOBAR_AA34623 | 13.206 | 24.2 | 0.605619311 | 0.02399796 | 0.003827168 |
| GOBAR_AA38944 | 20.99 | 37.153 | 0.603116672 | 0.042463915 | 0.00783802 |
| GOBAR_AA19677 | 13.11 | 23.313 | 0.60040699 | 0.002993011 | 0.000304988 |
| GOBAR_AA09268 | 41.61 | 74.143 | 0.595315076 | 0.00766812 | 0.000933624 |
| GOBAR_AA05227 | 43.99 | 77.49 | 0.586630811 | 0.003301747 | 0.000342297 |
| GOBAR_AA05678 | 12.316 | 21.303 | 0.585290734 | 0.026608341 | 0.004355685 |
| GOBAR_AA07787 | 18.546 | 32.45 | 0.580607851 | 0.002896932 | 0.000293969 |
| GOBAR_AA15608 | 2.53 | 5.516 | 0.580505992 | 0.029559452 | 0.004964287 |
| GOBAR_AA37376 | 5.956 | 10.493 | 0.58000637 | 0.012556771 | 0.001714908 |
| GOBAR_AA24035 | 85.416 | 149.026 | 0.57562425 | 0.002547105 | 0.00025332 |
| GOBAR_AA02449 | 6.42 | 12.68 | 0.574141672 | 0.033337943 | 0.005780862 |
| GOBAR_AA14727 | 143.376 | 249.806 | 0.573629025 | 0.000397855 | 2.98E-05 |
| GOBAR_AA20027 | 2.716 | 4.936 | 0.57185579 | 0.019565738 | 0.002975073 |
| GOBAR_AA24725 | 113.503 | 196.52 | 0.570510464 | 0.017623497 | 0.002603766 |
| GOBAR_AA08606 | 26.036 | 45.756 | 0.568201546 | 0.025861359 | 0.004198297 |
| GOBAR_AA09707 | 4.473 | 8.036 | 0.563128072 | 0.014109857 | 0.00198484 |
| GOBAR_AA17327 | 9.9 | 16.59 | 0.559959532 | 0.030217462 | 0.005107091 |
| GOBAR_AA39164 | 11.026 | 18.37 | 0.540783049 | 0.007771612 | 0.000949816 |
| GOBAR_AA08124 | 15.6 | 25.936 | 0.540387301 | 0.023249491 | 0.003681707 |
| GOBAR_AA16020 | 5.903 | 10.143 | 0.539395661 | 0.047288681 | 0.008962161 |
| GOBAR_AA14378 | 12.69 | 21.69 | 0.535722696 | 0.009730286 | 0.001255811 |
| GOBAR_AA39412 | 71.486 | 120.01 | 0.532397275 | 0.018820157 | 0.002824192 |
| GOBAR_AA27923 | 15.013 | 25.373 | 0.530941711 | 0.023072699 | 0.003648284 |
| GOBAR_AA03669 | 35.69 | 59.49 | 0.528847511 | 0.014073537 | 0.001976806 |
| GOBAR_AA34080 | 66.583 | 112.756 | 0.528389256 | 0.005380038 | 0.000613858 |
| GOBAR_AA02810 | 25.05 | 41.64 | 0.528136995 | 0.031790393 | 0.005449152 |
| GOBAR_AA10234 | 10.556 | 17.736 | 0.518030688 | 0.030795591 | 0.005237387 |
| GOBAR_AA23224 | 34.38 | 57.583 | 0.514393753 | 0.047648015 | 0.009049531 |
| GOBAR_AA00457 | 35.123 | 58.546 | 0.511893466 | 0.041057185 | 0.007496533 |
| GOBAR_AA11238 | 7.056 | 11.736 | 0.51084691 | 0.036681472 | 0.006503679 |
| GOBAR_AA06004 | 290.216 | 479.676 | 0.498942938 | 0.000357963 | 2.62E-05 |
| GOBAR_AA17876 | 45.99 | 75.7 | 0.498352171 | 0.039476074 | 0.007143985 |
| GOBAR_AA36458 | 71.233 | 110.48 | 0.497079263 | 0.009474542 | 0.001216735 |
| GOBAR_AA22234 | 118.076 | 194.96 | 0.495774294 | 0.025580595 | 0.004139417 |
| GOBAR_AA23195 | 71.906 | 116.956 | 0.493890624 | 0.026970391 | 0.004430691 |
| GOBAR_AA12203 | 22.383 | 36.673 | 0.474935974 | 0.034199797 | 0.005951055 |
| GOBAR_AA29866 | 28.783 | 47.036 | 0.472696544 | 0.04247388 | 0.007842313 |
| GOBAR_AA34363 | 16.646 | 26.696 | 0.472105875 | 0.035852469 | 0.006319413 |
| GOBAR_AA33041 | 41.56 | 67.743 | 0.47157208 | 0.023829619 | 0.00379688 |
| GOBAR_AA14980 | 14.703 | 24.753 | 0.469455514 | 0.047898309 | 0.009105369 |
| GOBAR_AA29557 | 15.956 | 25.83 | 0.469296748 | 0.040981757 | 0.007480393 |
| GOBAR_AA05611 | 17.57 | 28.31 | 0.467874956 | 0.032203755 | 0.005533959 |
| GOBAR_AA12880 | 7.786 | 12.64 | 0.465996034 | 0.032652215 | 0.005628944 |
| GOBAR_AA32000 | 20.006 | 32.21 | 0.461002934 | 0.03563247 | 0.006268285 |
| GOBAR_AA32398 | 65.61 | 105.22 | 0.460642396 | 0.042449651 | 0.007831709 |
| GOBAR_AA36702 | 29.88 | 47.81 | 0.453378591 | 0.014486613 | 0.002055468 |
| GOBAR_AA26227 | 16.296 | 25.746 | 0.444849036 | 0.026392515 | 0.004307395 |
| GOBAR_AA08111 | 48.74 | 77.59 | 0.442278567 | 0.02708288 | 0.0044584 |
| GOBAR_AA11484 | 7.193 | 11.036 | 0.42440909 | 0.037119488 | 0.006604929 |
| GOBAR_AA32717 | 40.89 | 64.366 | 0.422753978 | 0.026534753 | 0.004337507 |
| GOBAR_AA35141 | 30.623 | 47.966 | 0.416231654 | 0.041063168 | 0.007501184 |
| GOBAR_AA00785 | 11.376 | 19.586 | 0.415349548 | 0.048367389 | 0.009235057 |
| GOBAR_AA32857 | 82.126 | 119.863 | 0.394455326 | 0.045587107 | 0.008559352 |
| GOBAR_AA34625 | 41.866 | 64.273 | 0.378302548 | 0.028973903 | 0.004841677 |
| GOBAR_AA22441 | 29.18 | 43.44 | 0.349224343 | 0.049654954 | 0.009562656 |
| GOBAR_AA38884 | 17.923 | 16.58 | -0.371007626 | 0.038696378 | 0.006971585 |
| GOBAR_AA08033 | 61.006 | 55.403 | -0.3735477 | 0.044757721 | 0.008362256 |
| GOBAR_AA32183 | 54.556 | 48.906 | -0.379356073 | 0.039304903 | 0.007098248 |
| GOBAR_AA03780 | 21.013 | 18.776 | -0.393411935 | 0.04262923 | 0.007875922 |
| GOBAR_AA32494 | 22.97 | 20.373 | -0.396255329 | 0.031001323 | 0.005282556 |
| GOBAR_AA31679 | 18.193 | 16.19 | -0.397675376 | 0.049655514 | 0.009564198 |
| GOBAR_AA10194 | 57.933 | 68.61 | -0.400710854 | 0.02399796 | 0.003826883 |
| GOBAR_AA08453 | 17.3 | 14.73 | -0.424180054 | 0.030621876 | 0.00519667 |
| GOBAR_AA12023 | 43.286 | 37.683 | -0.424309491 | 0.033884601 | 0.005887399 |
| GOBAR_AA31839 | 45.166 | 38.643 | -0.443692173 | 0.04079083 | 0.007431404 |
| GOBAR_AA27946 | 22.943 | 19.643 | -0.444311565 | 0.034404342 | 0.005991617 |
| GOBAR_AA19097 | 17.626 | 15.176 | -0.44692266 | 0.008354419 | 0.001037937 |
| GOBAR_AA25437 | 58.21 | 50.27 | -0.447136241 | 0.035724041 | 0.006292649 |
| GOBAR_AA22536 | 16.013 | 13.75 | -0.44867783 | 0.005775468 | 0.000665983 |
| GOBAR_AA38383 | 36.61 | 31.313 | -0.452321104 | 0.019685459 | 0.002995789 |
| GOBAR_AA05607 | 20 | 17.196 | -0.457831221 | 0.005345016 | 0.000609244 |
| GOBAR_AA07935 | 32.743 | 30.783 | -0.459912167 | 0.025915901 | 0.004210894 |
| GOBAR_AA24775 | 34.063 | 28.703 | -0.462523404 | 0.024105994 | 0.003849968 |
| GOBAR_AA16182 | 7.573 | 6.383 | -0.475725317 | 0.011977036 | 0.001617396 |
| GOBAR_AA26084 | 15.636 | 14.01 | -0.482193531 | 0.03428073 | 0.005967119 |
| GOBAR_AA19299 | 21.616 | 18.053 | -0.489242141 | 0.01527733 | 0.002190167 |
| GOBAR_AA07184 | 40.053 | 33.223 | -0.491853414 | 0.023236956 | 0.003676942 |
| GOBAR_AA40141 | 50.423 | 43.026 | -0.492828137 | 0.034706739 | 0.006058315 |
| GOBAR_AA05202 | 56.033 | 46.17 | -0.495710526 | 0.01062299 | 0.00139496 |
| GOBAR_AA05879 | 28.03 | 23.276 | -0.496435839 | 0.012863152 | 0.001767155 |
| GOBAR_AA02776 | 38.46 | 31.7 | -0.498358018 | 0.007828193 | 0.000958766 |
| GOBAR_AA27050 | 11.573 | 9.816 | -0.499153162 | 0.006106315 | 0.000710454 |
| GOBAR_AA19953 | 38.926 | 32.62 | -0.501270076 | 0.015129371 | 0.002163711 |
| GOBAR_AA01569 | 16.77 | 13.863 | -0.501956813 | 0.017168164 | 0.002523228 |
| GOBAR_AA05739 | 10.6 | 8.613 | -0.511381297 | 0.014889676 | 0.00212298 |
| GOBAR_AA07825 | 34.926 | 31.696 | -0.511767254 | 0.00458718 | 0.000504639 |
| GOBAR_AA32260 | 28.033 | 22.983 | -0.514585776 | 0.022663573 | 0.003565917 |
| GOBAR_AA14245 | 32.036 | 26.366 | -0.51552388 | 0.049314105 | 0.009482769 |
| GOBAR_AA33110 | 64.606 | 52.876 | -0.520592818 | 0.024946064 | 0.004005753 |
| GOBAR_AA24311 | 9.816 | 7.95 | -0.522629506 | 0.041159467 | 0.007521153 |
| GOBAR_AA09729 | 11.446 | 9.286 | -0.522902 | 0.01347316 | 0.001873532 |
| GOBAR_AA40284 | 90.66 | 73.21 | -0.528755461 | 0.006586028 | 0.000775431 |
| GOBAR_AA23795 | 33.263 | 26.373 | -0.528814268 | 0.044898066 | 0.008397556 |
| GOBAR_AA39045 | 7.59 | 6.123 | -0.531530749 | 0.04221143 | 0.007773123 |
| GOBAR_AA07123 | 13.446 | 10.9 | -0.531841675 | 0.008902721 | 0.001124113 |
| GOBAR_AA05020 | 11.183 | 9.06 | -0.544836202 | 0.025636531 | 0.004152172 |
| GOBAR_AA15974 | 39.966 | 33.143 | -0.552601261 | 0.003729314 | 0.000395564 |
| GOBAR_AA06077 | 182.96 | 143.743 | -0.552923636 | 0.008958325 | 0.001134775 |
| GOBAR_AA08422 | 146.026 | 131.653 | -0.556407657 | 0.00069556 | 5.63E-05 |
| GOBAR_AA11738 | 23.763 | 18.983 | -0.560245851 | 0.000367133 | 2.71E-05 |
| GOBAR_AA35697 | 13.583 | 10.77 | -0.564303056 | 0.012437199 | 0.001694986 |
| GOBAR_AA07933 | 40.383 | 31.883 | -0.564467447 | 0.008162147 | 0.001010513 |
| GOBAR_AA18226 | 21.146 | 16.516 | -0.564891848 | 0.045366052 | 0.00849819 |
| GOBAR_AA21100 | 25.346 | 20.066 | -0.566413366 | 0.009256575 | 0.001182373 |
| GOBAR_AA15209 | 13.683 | 10.76 | -0.571571024 | 0.036135341 | 0.006380754 |
| GOBAR_AA08958 | 15.44 | 12.143 | -0.57186832 | 0.021312547 | 0.003301632 |
| GOBAR_AA27486 | 55.826 | 43.016 | -0.577489293 | 0.023249386 | 0.003680252 |
| GOBAR_AA09790 | 31.286 | 25.236 | -0.577857727 | 0.009163458 | 0.001168891 |
| GOBAR_AA29448 | 25.996 | 20.203 | -0.58002963 | 0.039365225 | 0.007114609 |
| GOBAR_AA32162 | 11.886 | 9.28 | -0.581485511 | 0.007767379 | 0.000948626 |
| GOBAR_AA00450 | 72.78 | 56.383 | -0.584025511 | 0.016395737 | 0.002382234 |
| GOBAR_AA20529 | 20.303 | 16.24 | -0.584781337 | 0.017644741 | 0.002608561 |
| GOBAR_AA07866 | 101.686 | 78.443 | -0.594220053 | 0.007639141 | 0.000929434 |
| GOBAR_AA19884 | 8.393 | 6.463 | -0.598366131 | 0.022369879 | 0.003508076 |
| GOBAR_AA05749 | 16.563 | 12.496 | -0.605672869 | 0.005791047 | 0.000667947 |
| GOBAR_AA17635 | 15.323 | 11.783 | -0.608504984 | 0.002046731 | 0.000196638 |
| GOBAR_AA08993 | 3.09 | 2.346 | -0.611115271 | 0.037364396 | 0.006669014 |
| GOBAR_AA05082 | 35 | 26.666 | -0.611792871 | 0.000710527 | 5.77E-05 |
| GOBAR_AA39010 | 33.66 | 25.35 | -0.614349765 | 0.01738247 | 0.002562256 |
| GOBAR_AA11269 | 85.346 | 64.796 | -0.616250643 | 0.021415145 | 0.003323093 |
| GOBAR_AA28238 | 6.503 | 5.333 | -0.619347387 | 0.025095869 | 0.00403645 |
| GOBAR_AA28774 | 7.706 | 5.543 | -0.622413421 | 0.046886843 | 0.008871106 |
| GOBAR_AA37267 | 13.253 | 9.92 | -0.62411226 | 0.017244695 | 0.002536966 |
| GOBAR_AA31804 | 11.58 | 8.7 | -0.626417022 | 0.003489903 | 0.00036513 |
| GOBAR_AA06265 | 41.423 | 31.476 | -0.629974376 | 0.001561597 | 0.000144481 |
| GOBAR_AA00604 | 36.553 | 27.476 | -0.631478194 | 0.015474139 | 0.002225086 |
| GOBAR_AA19525 | 29.26 | 22.003 | -0.632927654 | 0.036942303 | 0.006561663 |
| GOBAR_AA19778 | 9.556 | 7.283 | -0.63475442 | 0.021047554 | 0.003250853 |
| GOBAR_AA36768 | 13.206 | 9.87 | -0.635519312 | 0.008023448 | 0.000990097 |
| GOBAR_AA37054 | 32.573 | 24.056 | -0.636026804 | 0.042239538 | 0.007785159 |
| GOBAR_AA36948 | 28.216 | 21.006 | -0.636570521 | 0.03749489 | 0.006704219 |
| GOBAR_AA39375 | 13.343 | 9.87 | -0.636912156 | 0.026637709 | 0.004362801 |
| GOBAR_AA34342 | 68.103 | 52.96 | -0.637046525 | 0.02560524 | 0.004144885 |
| GOBAR_AA13445 | 49.616 | 37.18 | -0.649575033 | 0.003229815 | 0.0003326 |
| GOBAR_AA10987 | 11.263 | 7.35 | -0.65204744 | 0.007071313 | 0.000846866 |
| GOBAR_AA21189 | 7.456 | 5.106 | -0.652317251 | 0.048629716 | 0.009299408 |
| GOBAR_AA29232 | 39.776 | 29.316 | -0.656080966 | 0.001680418 | 0.000157319 |
| GOBAR_AA30228 | 19.333 | 14.27 | -0.657438571 | 0.004758274 | 0.000527176 |
| GOBAR_AA00034 | 13.046 | 9.563 | -0.658125667 | 0.000137935 | 8.69E-06 |
| GOBAR_AA01991 | 30.633 | 22.716 | -0.660815462 | 0.005137592 | 0.000579945 |
| GOBAR_AA39438 | 42.343 | 29.003 | -0.665478097 | 0.013310719 | 0.001841715 |
| GOBAR_AA33386 | 23.13 | 17.49 | -0.667442327 | 3.61E-05 | 1.94E-06 |
| GOBAR_AA25555 | 8.76 | 6.396 | -0.673422288 | 0.004369407 | 0.000477468 |
| GOBAR_AA35944 | 7.9 | 5.79 | -0.674839312 | 0.009149478 | 0.001166579 |
| GOBAR_AA30173 | 71.42 | 52.43 | -0.678471904 | 4.84E-07 | 1.74E-08 |
| GOBAR_AA25580 | 7.666 | 5.126 | -0.678804252 | 0.014334168 | 0.002029593 |
| GOBAR_AA16963 | 12.196 | 8.883 | -0.682669517 | 0.002128403 | 0.00020553 |
| GOBAR_AA30196 | 53.563 | 36.933 | -0.682808749 | 0.019598142 | 0.002981132 |
| GOBAR_AA20311 | 21.136 | 15.96 | -0.683700424 | 0.007937549 | 0.000976746 |
| GOBAR_AA34168 | 14.813 | 10.613 | -0.694587656 | 0.002781482 | 0.000280511 |
| GOBAR_AA10859 | 14.233 | 10.483 | -0.698325269 | 0.00248168 | 0.000245738 |
| GOBAR_AA09918 | 19.966 | 14.793 | -0.699045222 | 0.032495128 | 0.005594355 |
| GOBAR_AA23754 | 3.603 | 2.576 | -0.700954089 | 0.003257824 | 0.000336324 |
| GOBAR_AA07983 | 13.293 | 9.016 | -0.706083074 | 0.013903366 | 0.001947813 |
| GOBAR_AA35094 | 43.87 | 32.41 | -0.706418511 | 0.015475231 | 0.00222569 |
| GOBAR_AA07102 | 58.703 | 42.056 | -0.706695502 | 0.000254906 | 1.78E-05 |
| GOBAR_AA31115 | 45.05 | 31.936 | -0.70826292 | 0.004882853 | 0.00054373 |
| GOBAR_AA09691 | 22.893 | 16.636 | -0.71031798 | 0.000244228 | 1.69E-05 |
| GOBAR_AA27828 | 8.446 | 6.01 | -0.714630432 | 0.001259234 | 0.000112012 |
| GOBAR_AA14379 | 44.34 | 31.503 | -0.71734302 | 4.69E-05 | 2.60E-06 |
| GOBAR_AA19089 | 158.623 | 112.46 | -0.717864166 | 3.80E-05 | 2.05E-06 |
| GOBAR_AA30868 | 29.383 | 20.456 | -0.719568583 | 0.001026343 | 8.86E-05 |
| GOBAR_AA20826 | 11.146 | 7.816 | -0.726842268 | 0.002216785 | 0.000215346 |
| GOBAR_AA24869 | 2.846 | 2.073 | -0.730466793 | 0.012285978 | 0.001669053 |
| GOBAR_AA37591 | 13.083 | 9.023 | -0.733967084 | 0.007365719 | 0.00088885 |
| GOBAR_AA12983 | 7.33 | 5.23 | -0.736598221 | 0.012192941 | 0.001652892 |
| GOBAR_AA25641 | 19.086 | 13.43 | -0.738106752 | 3.98E-05 | 2.17E-06 |
| GOBAR_AA34792 | 6.843 | 4.676 | -0.742104946 | 0.0073933 | 0.000893598 |
| GOBAR_AA32390 | 844.586 | 581.856 | -0.743969132 | 0.006857251 | 0.000816278 |
| GOBAR_AA19189 | 20.12 | 15.543 | -0.763686439 | 1.64E-05 | 8.10E-07 |
| GOBAR_AA03672 | 30.813 | 21.16 | -0.766049878 | 0.00084874 | 7.12E-05 |
| GOBAR_AA12008 | 590.606 | 405.15 | -0.766646039 | 0.03251201 | 0.005599139 |
| GOBAR_AA27142 |  | 44.723 | -0.770549527 | 0.000498829 | 3.84E-05 |
| GOBAR_AA23667 | 8.21 | 5.583 | -0.771084593 | 0.003414444 | 0.000356249 |
| GOBAR_AA24691 | 15.66 | 10.976 | -0.77337239 | 0.020086678 | 0.003072271 |
| GOBAR_AA27327 | 16.49 | 11.27 | -0.774517421 | 0.001168647 | 0.000103097 |
| GOBAR_AA03797 | 108.936 | 72.57 | -0.776045298 | 0.004734014 | 0.000523327 |
| GOBAR_AA31731 | 10.35 | 6.98 | -0.785770575 | 0.004293867 | 0.000467229 |
| GOBAR_AA38360 | 7.16 | 4.843 | -0.78585859 | 8.00E-05 | 4.71E-06 |
| GOBAR_AA22784 | 25.446 | 17.033 | -0.788414898 | 0.002019123 | 0.000193344 |
| GOBAR_AA11548 | 35.95 | 24.3 | -0.789625912 | 0.000395346 | 2.95E-05 |
| GOBAR_AA13954 | 8.306 | 5.536 | -0.791980972 | 0.02505891 | 0.004027493 |
| GOBAR_AA20279 | 8.34 | 5.56 | -0.801349708 | 0.012927357 | 0.001778216 |
| GOBAR_AA17117 | 17.553 | 11.84 | -0.801496842 | 0.000791676 | 6.55E-05 |
| GOBAR_AA02747 | 18.21 | 11.4 | -0.803039445 | 0.000408426 | 3.06E-05 |
| GOBAR_AA39282 | 12.846 | 7.523 | -0.804107465 | 0.027154258 | 0.004473781 |
| GOBAR_AA34250 | 55.273 | 36.126 | -0.808596095 | 6.56E-07 | 2.40E-08 |
| GOBAR_AA34173 | 34.116 | 21.006 | -0.81045071 | 0.006103217 | 0.000709765 |
| GOBAR_AA05688 | 4.093 | 2.433 | -0.815196955 | 0.039691151 | 0.007193226 |
| GOBAR_AA31796 | 5.7 | 3.78 | -0.816392552 | 0.003762329 | 0.000399718 |
| GOBAR_AA39552 | 22.423 | 14.796 | -0.817152429 | 0.000322143 | 2.33E-05 |
| GOBAR_AA38154 | 12.39 | 8.126 | -0.821896764 | 0.000175281 | 1.14E-05 |
| GOBAR_AA10179 | 5.42 | 3.666 | -0.822692693 | 0.017163676 | 0.002521594 |
| GOBAR_AA40383 | 5.56 | 3.61 | -0.823565925 | 0.012760337 | 0.001749531 |
| GOBAR_AA23805 | 297.216 | 191.283 | -0.831506623 | 0.02654415 | 0.004340577 |
| GOBAR_AA19813 | 16.253 | 10.493 | -0.835523778 | 0.002423077 | 0.000238885 |
| GOBAR_AA38168 | 18.806 | 12.296 | -0.836221905 | 0.000267586 | 1.88E-05 |
| GOBAR_AA11010 | 15.216 | 9.92 | -0.844859226 | 0.002492723 | 0.000247191 |
| GOBAR_AA09738 | 26.993 | 17.536 | -0.845652272 | 5.99E-05 | 3.41E-06 |
| GOBAR_AA26433 | 68.726 | 43.693 | -0.846428715 | 6.97E-07 | 2.57E-08 |
| GOBAR_AA23338 | 155.063 | 100.746 | -0.846666508 | 0.000735865 | 6.00E-05 |
| GOBAR_AA16811 | 41.436 | 27.093 | -0.850940052 | 0.000986455 | 8.47E-05 |
| GOBAR_AA29749 | 8.183 | 5.253 | -0.851485011 | 0.001289722 | 0.000115341 |
| GOBAR_AA08350 | 12.153 | 7.766 | -0.854028659 | 0.00053399 | 4.15E-05 |
| GOBAR_AA01019 | 11.65 | 7.44 | -0.854822901 | 0.048885745 | 0.009377805 |
| GOBAR_AA13847 | 7.916 | 5.046 | -0.856700637 | 0.003700523 | 0.000391525 |
| GOBAR_AA03366 | 27.846 | 17.546 | -0.860052151 | 0.006530706 | 0.000766654 |
| GOBAR_AA10643 | 3.11 | 1.986 | -0.860128684 | 0.009791776 | 0.001267141 |
| GOBAR_AA28338 | 13.24 | 7.956 | -0.866846728 | 0.000417149 | 3.14E-05 |
| GOBAR_AA39102 | 10.403 | 6.456 | -0.869138261 | 0.024320317 | 0.003891011 |
| GOBAR_AA24000 | 18.7 | 11.646 | -0.869900766 | 0.024408704 | 0.003906775 |
| GOBAR_AA32355 | 14.733 | 9.383 | -0.873328291 | 0.04541663 | 0.008514224 |
| GOBAR_AA29465 | 7.256 | 4.583 | -0.874781844 | 0.006810706 | 0.000808524 |
| GOBAR_AA00055 | 18.563 | 11.806 | -0.874794418 | 0.000164697 | 1.06E-05 |
| GOBAR_AA38083 | 10.276 | 6.52 | -0.8749366 | 0.016355048 | 0.00237585 |
| GOBAR_AA22501 | 6.166 | 3.683 | -0.874961666 | 0.018448799 | 0.002762071 |
| GOBAR_AA02514 | 13.686 | 8.65 | -0.875486602 | 0.000522955 | 4.05E-05 |
| GOBAR_AA22131 | 19.763 | 12.083 | -0.881159373 | 0.000168353 | 1.09E-05 |
| GOBAR_AA16342 | 12.1 | 7.63 | -0.886669054 | 0.000396825 | 2.96E-05 |
| GOBAR_AA39076 | 9.576 | 6.016 | -0.89005946 | 0.023727326 | 0.003779895 |
| GOBAR_AA22497 | 195.786 | 123.05 | -0.89206064 | 6.39E-06 | 2.87E-07 |
| GOBAR_AA23115 | 7.3 | 4.523 | -0.894198695 | 1.54E-06 | 6.10E-08 |
| GOBAR_AA38830 | 18.34 | 11.483 | -0.904050791 | 0.001803722 | 0.000170247 |
| GOBAR_AA20655 | 310.123 | 191.606 | -0.904967462 | 1.16E-06 | 4.49E-08 |
| GOBAR_AA00737 | 15.07 | 9.416 | -0.905207581 | 0.000218772 | 1.49E-05 |
| GOBAR_AA05468 | 99.65 | 75.42 | -0.906547447 | 3.09E-05 | 1.63E-06 |
| GOBAR_AA01134 | 99.216 | 58.786 | -0.907304131 | 0.009474542 | 0.001216783 |
| GOBAR_AA21096 | 8.92 | 5.06 | -0.909248611 | 0.00085309 | 7.17E-05 |
| GOBAR_AA02218 | 69.77 | 38.96 | -0.909630978 | 0.004849079 | 0.000539129 |
| GOBAR_AA27426 | 7.85 | 4.776 | -0.909788198 | 1.81E-05 | 9.02E-07 |
| GOBAR_AA01433 | 22.086 | 13.556 | -0.912736655 | 3.61E-05 | 1.94E-06 |
| GOBAR_AA02663 | 10.213 | 6.313 | -0.917096397 | 0.018674538 | 0.002800722 |
| GOBAR_AA13750 | 9.473 | 5.78 | -0.919536103 | 0.002541684 | 0.000252634 |
| GOBAR_AA03934 | 12.026 | 7.26 | -0.926964419 | 0.01578146 | 0.002278324 |
| GOBAR_AA14420 | 2.386 | 1.44 | -0.928755372 | 0.029979818 | 0.005056534 |
| GOBAR_AA35089 | 275 | 170.3 | -0.937260709 | 0.009005217 | 0.001142293 |
| GOBAR_AA11108 | 42.526 | 25.3 | -0.942715812 | 0.001846577 | 0.000174795 |
| GOBAR_AA15741 | 68.893 | 41.14 | -0.943068653 | 1.30E-05 | 6.25E-07 |
| GOBAR_AA23099 | 10.39 | 6.48 | -0.943420819 | 0.005111091 | 0.000575494 |
| GOBAR_AA36706 | 5.38 | 3.14 | -0.949061015 | 0.013918768 | 0.001950372 |
| GOBAR_AA33982 | 4.886 | 2.696 | -0.950297911 | 0.034304578 | 0.005972261 |
| GOBAR_AA35832 | 58.73 | 35.293 | -0.958934852 | 4.73E-07 | 1.70E-08 |
| GOBAR_AA38613 | 40.78 | 24.03 | -0.970046298 | 1.31E-06 | 5.16E-08 |
| GOBAR_AA14312 | 20.566 | 11.853 | -0.971243995 | 1.84E-06 | 7.47E-08 |
| GOBAR_AA30334 | 47.76 | 29.316 | -0.971669057 | 0.002221645 | 0.000216074 |
| GOBAR_AA28985 | 13.036 | 7.636 | -0.972845516 | 0.014859204 | 0.002117078 |
| GOBAR_AA06526 | 71.386 | 41.566 | -0.981433534 | 3.97E-05 | 2.15E-06 |
| GOBAR_AA39004 | 42.806 | 25.21 | -0.984436476 | 1.44E-07 | 4.74E-09 |
| GOBAR_AA34478 | 16.323 | 9.256 | -0.986219653 | 0.024417127 | 0.003908828 |
| GOBAR_AA28168 | 41.673 | 24.506 | -0.988927941 | 0.004784526 | 0.000530432 |
| GOBAR_AA14979 | 55.6 | 32.236 | -0.989593227 | 1.76E-10 | 3.91E-12 |
| GOBAR_AA03757 | 3.763 | 2.156 | -0.997874834 | 0.028072178 | 0.004662614 |
| GOBAR_AA00352 | 37.373 | 22.956 | -0.998864094 | 4.60E-07 | 1.64E-08 |
| GOBAR_AA00467 | 11.643 | 6.58 | -1.002536216 | 0.035544791 | 0.006251834 |
| GOBAR_AA18771 | 10.07 | 5.743 | -1.015020637 | 0.003189512 | 0.000327914 |
| GOBAR_AA08410 | 2.19 | 1.25 | -1.017426375 | 0.012232703 | 0.001660049 |
| GOBAR_AA36268 | 37.216 | 21.56 | -1.019699448 | 6.52E-05 | 3.75E-06 |
| GOBAR_AA35339 | 6.66 | 3.65 | -1.02190166 | 0.000145076 | 9.22E-06 |
| GOBAR_AA28294 | 3.716 | 2.203 | -1.023550852 | 0.026881613 | 0.004410513 |
| GOBAR_AA40147 | 7.973 | 4.556 | -1.030145196 | 1.79E-05 | 8.95E-07 |
| GOBAR_AA30061 | 5.953 | 3.273 | -1.037028029 | 0.023688559 | 0.003769614 |
| GOBAR_AA11838 | 8.843 | 5.173 | -1.041862207 | 6.94E-05 | 4.04E-06 |
| GOBAR_AA11581 | 19.693 | 11.043 | -1.042647852 | 7.12E-06 | 3.23E-07 |
| GOBAR_AA16118 | 6.92 | 4.213 | -1.050470555 | 0.000134501 | 8.44E-06 |
| GOBAR_AA15131 | 5.316 | 2.853 | -1.051177014 | 0.008558065 | 0.001071396 |
| GOBAR_AA03260 | 127.503 | 70.89 | -1.051504968 | 2.02E-07 | 6.80E-09 |
| GOBAR_AA36233 | 30.576 | 16.836 | -1.052975907 | 0.000192949 | 1.28E-05 |
| GOBAR_AA28430 | 5.566 | 2.993 | -1.055336573 | 0.049719802 | 0.009583995 |
| GOBAR_AA40283 | 31.766 | 17.533 | -1.056609388 | 4.04E-05 | 2.20E-06 |
| GOBAR_AA33921 | 7.933 | 4.41 | -1.059161629 | 4.53E-05 | 2.51E-06 |
| GOBAR_AA09283 | 4.03 | 2.2 | -1.063191444 | 0.014170151 | 0.00199565 |
| GOBAR_AA28913 | 6.67 | 3.48 | -1.079714757 | 0.032368329 | 0.00556411 |
| GOBAR_AA33567 | 13.723 | 7.443 | -1.112171199 | 7.84E-08 | 2.47E-09 |
| GOBAR_AA00282 | 8.58 | 4.47 | -1.118705339 | 0.00507623 | 0.000570249 |
| GOBAR_AA30381 | 5.02 | 2.636 | -1.12011561 | 0.022701527 | 0.003573856 |
| GOBAR_AA33387 | 5.77 | 2.913 | -1.148153261 | 0.005374452 | 0.000613065 |
| GOBAR_AA38014 | 271.076 | 147.903 | -1.153225656 | 1.21E-06 | 4.73E-08 |
| GOBAR_AA07448 | 26.47 | 13.636 | -1.157217891 | 3.04E-10 | 6.95E-12 |
| GOBAR_AA26291 | 11.916 | 6.02 | -1.162086901 | 2.24E-05 | 1.13E-06 |
| GOBAR_AA35052 | 2.98 | 1.496 | -1.163853718 | 0.026889215 | 0.004414867 |
| GOBAR_AA39841 | 12.033 | 5.666 | -1.170180627 | 0.00160078 | 0.000148558 |
| GOBAR_AA09331 | 78.063 | 41.366 | -1.173925029 | 0.000382265 | 2.84E-05 |
| GOBAR_AA24944 | 83.75 | 44.083 | -1.180083933 | 0.000767986 | 6.31E-05 |
| GOBAR_AA05694 | 87.283 | 44.163 | -1.182316652 | 1.89E-06 | 7.67E-08 |
| GOBAR_AA24402 | 19.586 | 9.946 | -1.188037841 | 1.90E-12 | 3.40E-14 |
| GOBAR_AA28714 | 10.753 | 5.793 | -1.195553119 | 0.013798438 | 0.001929127 |
| GOBAR_AA38583 | 140.246 | 71.75 | -1.196034958 | 9.62E-06 | 4.50E-07 |
| GOBAR_AA40168 | 33.486 | 16.833 | -1.196096056 | 0.000694332 | 5.62E-05 |
| GOBAR_AA25667 | 7.7 | 4.723 | -1.203208353 | 0.000842938 | 7.06E-05 |
| GOBAR_AA35715 | 42.806 | 20.743 | -1.207034216 | 0.026535151 | 0.004338339 |
| GOBAR_AA39922 | 6.453 | 3.206 | -1.209364829 | 0.000382945 | 2.85E-05 |
| GOBAR_AA12578 | 70.016 | 36.3 | -1.209525262 | 4.27E-12 | 8.01E-14 |
| GOBAR_AA37678 | 18.063 | 8.933 | -1.212305313 | 0.00023123 | 1.58E-05 |
| GOBAR_AA13316 | 28.27 | 15.846 | -1.217295753 | 3.59E-11 | 7.48E-13 |
| GOBAR_AA03662 | 3.36 | 1.98 | -1.218030939 | 0.000848404 | 7.11E-05 |
| GOBAR_AA29494 | 34.83 | 17.38 | -1.225480309 | 1.08E-06 | 4.15E-08 |
| GOBAR_AA33208 | 9.11 | 4.496 | -1.226950775 | 5.12E-09 | 1.38E-10 |
| GOBAR_AA38917 | 9.033 | 4.336 | -1.22797756 | 1.67E-06 | 6.68E-08 |
| GOBAR_AA39918 | 3.763 | 1.846 | -1.230490023 | 0.001575269 | 0.000145792 |
| GOBAR_AA20995 | 37.926 | 18.19 | -1.233239435 | 0.013156513 | 0.001817719 |
| GOBAR_AA29218 | 93.163 | 44.993 | -1.236076332 | 8.06E-05 | 4.75E-06 |
| GOBAR_AA22000 | 11.443 | 5.53 | -1.238398172 | 5.32E-05 | 2.98E-06 |
| GOBAR_AA30170 | 89.93 | 42.126 | -1.249787888 | 4.41E-10 | 1.04E-11 |
| GOBAR_AA05834 | 8.446 | 3.966 | -1.281284465 | 0.00119629 | 0.000105845 |
| GOBAR_AA00953 | 3.163 | 1.47 | -1.282605708 | 0.010104051 | 0.001316892 |
| GOBAR_AA20760 | 3.71 | 1.663 | -1.284561514 | 0.02468735 | 0.003960644 |
| GOBAR_AA20308 | 10.39 | 4.903 | -1.291254253 | 0.001365022 | 0.000122942 |
| GOBAR_AA17527 | 3.616 | 1.696 | -1.293901924 | 0.041235293 | 0.007544538 |
| GOBAR_AA12977 | 5.493 | 2.39 | -1.297759015 | 0.031313973 | 0.005344625 |
| GOBAR_AA24959 | 24.096 | 10.136 | -1.298821647 | 0.000214083 | 1.44E-05 |
| GOBAR_AA35355 | 96.713 | 44.82 | -1.301496692 | 1.42E-06 | 5.64E-08 |
| GOBAR_AA23779 | 8.2 | 3.756 | -1.307500384 | 0.00021437 | 1.45E-05 |
| GOBAR_AA14759 | 4.93 | 2.256 | -1.332404442 | 0.000682453 | 5.50E-05 |
| GOBAR_AA32144 | 11.626 | 5.34 | -1.33857814 | 0.001810001 | 0.000171019 |
| GOBAR_AA02249 | 8.126 | 3.616 | -1.343614748 | 6.59E-06 | 2.96E-07 |
| GOBAR_AA16776 | 73.593 | 32.236 | -1.345932365 | 0.000940584 | 8.01E-05 |
| GOBAR_AA22143 | 75.206 | 34.126 | -1.350020915 | 0.000111514 | 6.85E-06 |
| GOBAR_AA35762 | 3.56 | 1.483 | -1.355362854 | 0.03514157 | 0.006155535 |
| GOBAR_AA22216 | 1.853 | 0.786 | -1.358652307 | 0.027277202 | 0.004497969 |
| GOBAR_AA39092 | 4.413 | 1.96 | -1.361274388 | 9.99E-06 | 4.71E-07 |
| GOBAR_AA31898 | 114.24 | 51.573 | -1.362270363 | 5.56E-18 | 6.28E-20 |
| GOBAR_AA24010 | 64.043 | 29.716 | -1.369388471 | 2.49E-17 | 2.94E-19 |
| GOBAR_AA26877 | 28.203 | 13.303 | -1.370922106 | 3.33E-09 | 8.74E-11 |
| GOBAR_AA06281 | 5.653 | 2.456 | -1.371368019 | 0.009763574 | 0.001262081 |
| GOBAR_AA22247 | 1.446 | 0.616 | -1.379960769 | 0.048922638 | 0.009386295 |
| GOBAR_AA08934 | 1.433 | 0.59 | -1.383924915 | 0.043923387 | 0.008171933 |
| GOBAR_AA13919 | 3.233 | 1.3 | -1.401869258 | 0.026269004 | 0.004280408 |
| GOBAR_AA17952 | 47.926 | 20.693 | -1.404463692 | 1.54E-05 | 7.56E-07 |
| GOBAR_AA31016 | 188.64 | 75.71 | -1.406442896 | 0.018256578 | 0.002726437 |
| GOBAR_AA03547 | 37.016 | 16.14 | -1.409226072 | 9.24E-14 | 1.45E-15 |
| GOBAR_AA12921 | 5.76 | 2.46 | -1.414533037 | 5.91E-05 | 3.36E-06 |
| GOBAR_AA39845 | 4.943 | 2.013 | -1.415757859 | 0.041953174 | 0.007703145 |
| GOBAR_AA26509 | 27.273 | 11.736 | -1.420754455 | 2.36E-12 | 4.29E-14 |
| GOBAR_AA08598 | 144.443 | 61.153 | -1.420954584 | 0.009939835 | 0.001289402 |
| GOBAR_AA29440 | 61.793 | 26.83 | -1.421502695 | 9.85E-17 | 1.21E-18 |
| GOBAR_AA05331 | 0.823 | 0.336 | -1.426679714 | 0.042043568 | 0.007734928 |
| GOBAR_AA01056 | 21.446 | 9.01 | -1.443486017 | 1.04E-06 | 3.98E-08 |
| GOBAR_AA21395 | 7.213 | 3.016 | -1.444738784 | 0.00058501 | 4.61E-05 |
| GOBAR_AA28493 | 5.346 | 2.22 | -1.447754156 | 2.65E-05 | 1.37E-06 |
| GOBAR_AA19185 | 108.116 | 46.673 | -1.451789776 | 3.01E-10 | 6.88E-12 |
| GOBAR_AA15682 | 25.83 | 10.773 | -1.452820291 | 0.00036795 | 2.71E-05 |
| GOBAR_AA27175 | 9.773 | 4.133 | -1.457939275 | 1.29E-08 | 3.65E-10 |
| GOBAR_AA01555 | 30.7 | 12.48 | -1.465254946 | 9.18E-13 | 1.61E-14 |
| GOBAR_AA08603 | 2.873 | 1.113 | -1.475145456 | 0.025552317 | 0.004134104 |
| GOBAR_AA24401 | 14.953 | 6.183 | -1.478584553 | 1.69E-05 | 8.38E-07 |
| GOBAR_AA27164 | 3.956 | 1.55 | -1.520135418 | 0.00524034 | 0.000594289 |
| GOBAR_AA34732 | 33.81 | 15.066 | -1.547217874 | 1.99E-09 | 5.12E-11 |
| GOBAR_AA36626 | 20.526 | 8.016 | -1.547930839 | 2.17E-10 | 4.89E-12 |
| GOBAR_AA21188 | 10.503 | 3.873 | -1.552514085 | 0.012142096 | 0.00164438 |
| GOBAR_AA26636 | 65.003 | 25.736 | -1.562144295 | 1.00E-14 | 1.45E-16 |
| GOBAR_AA12786 | 466.706 | 179.013 | -1.570768611 | 7.22E-06 | 3.28E-07 |
| GOBAR_AA20380 | 13.193 | 4.853 | -1.593111729 | 0.000254716 | 1.77E-05 |
| GOBAR_AA30639 | 1.106 | 0.366 | -1.5935153 | 0.034462148 | 0.00600467 |
| GOBAR_AA22689 | 5.33 | 2.136 | -1.59890927 | 0.000254318 | 1.77E-05 |
| GOBAR_AA23502 | 2.53 | 0.903 | -1.599704615 | 0.006807551 | 0.000807806 |
| GOBAR_AA20016 | 65.753 | 24.65 | -1.606112255 | 2.44E-11 | 4.93E-13 |
| GOBAR_AA04696 | 1.286 | 0.44 | -1.607034908 | 0.019043529 | 0.002873665 |
| GOBAR_AA24413 | 9.45 | 3.65 | -1.62523452 | 2.79E-13 | 4.61E-15 |
| GOBAR_AA27427 | 8.266 | 2.93 | -1.630824758 | 2.87E-06 | 1.21E-07 |
| GOBAR_AA37796 | 165.433 | 58.566 | -1.645319591 | 3.85E-13 | 6.53E-15 |
| GOBAR_AA09278 | 0.84 | 0.276 | -1.656769601 | 0.015524761 | 0.002235134 |
| GOBAR_AA14037 | 9.75 | 4.293 | -1.675143996 | 6.03E-16 | 7.84E-18 |
| GOBAR_AA14393 | 21.353 | 7.643 | -1.683009824 | 7.09E-13 | 1.22E-14 |
| GOBAR_AA06873 | 4.406 | 1.493 | -1.687631039 | 0.002042058 | 0.000196116 |
| GOBAR_AA33377 | 4.383 | 1.703 | -1.701547552 | 8.25E-09 | 2.28E-10 |
| GOBAR_AA04421 | 157.296 | 53.43 | -1.764827458 | 5.06E-20 | 4.93E-22 |
| GOBAR_AA08019 | 3.183 | 1.086 | -1.765427221 | 0.000181765 | 1.20E-05 |
| GOBAR_AA12784 | 260.616 | 87.536 | -1.770224712 | 2.86E-11 | 5.83E-13 |
| GOBAR_AA35155 | 2.193 | 0.653 | -1.775315218 | 0.01107709 | 0.001469569 |
| GOBAR_AA30841 | 7.12 | 2.31 | -1.784219584 | 8.69E-08 | 2.75E-09 |
| GOBAR_AA21664 | 4.29 | 1.376 | -1.791118969 | 0.005122277 | 0.000577641 |
| GOBAR_AA23674 | 100.376 | 30.743 | -1.800929396 | 0.000130208 | 8.14E-06 |
| GOBAR_AA39373 | 121.793 | 39.483 | -1.811055793 | 9.77E-12 | 1.90E-13 |
| GOBAR_AA08176 | 72.81 | 23.693 | -1.819958286 | 8.92E-13 | 1.56E-14 |
| GOBAR_AA04298 | 189.073 | 61.096 | -1.835270935 | 1.66E-22 | 1.34E-24 |
| GOBAR_AA19970 | 39.163 | 12.636 | -1.836256783 | 7.88E-26 | 5.26E-28 |
| GOBAR_AA24230 | 2.806 | 0.813 | -1.846538632 | 0.001669391 | 0.000155949 |
| GOBAR_AA31881 | 7.253 | 1.873 | -1.895899329 | 0.011049287 | 0.001464025 |
| GOBAR_AA12553 | 0.326 | 0.086 | -1.90587421 | 0.010744302 | 0.001414925 |
| GOBAR_AA39940 | 3.736 | 1.103 | -1.924663759 | 4.48E-08 | 1.35E-09 |
| GOBAR_AA06036 | 6.943 | 1.953 | -1.942371847 | 0.000409778 | 3.07E-05 |
| GOBAR_AA03171 | 428.13 | 126.35 | -1.95233553 | 2.96E-25 | 2.05E-27 |
| GOBAR_AA11014 | 0.74 | 0.193 | -1.962418114 | 0.002403293 | 0.000236587 |
| GOBAR_AA38025 | 1.62 | 0.4 | -1.965862208 | 0.00662764 | 0.000781096 |
| GOBAR_AA20705 | 4.086 | 1.16 | -1.96728627 | 0.000211087 | 1.42E-05 |
| GOBAR_AA11752 | 109.886 | 32.603 | -1.975616677 | 1.63E-12 | 2.91E-14 |
| GOBAR_AA30597 | 9.59 | 2.576 | -1.986265214 | 1.57E-06 | 6.25E-08 |
| GOBAR_AA37155 | 93.183 | 26.653 | -1.98840379 | 3.92E-10 | 9.13E-12 |
| GOBAR_AA05154 | 2.923 | 0.836 | -1.994714224 | 0.000161151 | 1.04E-05 |
| GOBAR_AA03589 | 20.466 | 5.576 | -1.99763991 | 1.48E-24 | 1.08E-26 |
| GOBAR_AA29872 | 2.256 | 0.593 | -2.009776774 | 0.000582093 | 4.58E-05 |
| GOBAR_AA08801 | 0.616 | 0.136 | -2.021591429 | 0.021493877 | 0.003339656 |
| GOBAR_AA31390 | 10.883 | 3.156 | -2.026402878 | 3.82E-06 | 1.65E-07 |
| GOBAR_AA26978 | 24.976 | 6.926 | -2.029721067 | 5.07E-14 | 7.81E-16 |
| GOBAR_AA08880 | 2 | 0.516 | -2.03362808 | 0.000195799 | 1.30E-05 |
| GOBAR_AA21203 | 0.67 | 0.166 | -2.042932439 | 0.007769153 | 0.000949067 |
| GOBAR_AA32186 | 0.973 | 0.213 | -2.054003909 | 0.011823011 | 0.001590791 |
| GOBAR_AA12233 | 14.143 | 3.736 | -2.059700091 | 8.27E-12 | 1.60E-13 |
| GOBAR_AA26005 | 5.903 | 1.49 | -2.134198678 | 0.000240724 | 1.66E-05 |
| GOBAR_AA38713 | 21.656 | 5.39 | -2.149193167 | 5.06E-18 | 5.66E-20 |
| GOBAR_AA05372 | 2.083 | 0.343 | -2.155110203 | 0.028421093 | 0.004734717 |
| GOBAR_AA33975 | 1.873 | 0.39 | -2.166247759 | 0.003563403 | 0.000374364 |
| GOBAR_AA34785 | 4.17 | 0.943 | -2.172115414 | 0.00030982 | 2.22E-05 |
| GOBAR_AA35193 | 2.27 | 0.736 | -2.175277735 | 3.18E-06 | 1.36E-07 |
| GOBAR_AA31103 | 119.336 | 28.536 | -2.207064399 | 1.42E-09 | 3.59E-11 |
| GOBAR_AA25446 | 2.313 | 0.536 | -2.210917767 | 8.98E-06 | 4.17E-07 |
| GOBAR_AA07152 | 23.886 | 5.396 | -2.226733642 | 2.53E-08 | 7.39E-10 |
| GOBAR_AA25392 | 181.18 | 43.25 | -2.236421619 | 1.13E-10 | 2.47E-12 |
| GOBAR_AA40450 | 1.563 | 0.363 | -2.247956838 | 4.62E-07 | 1.66E-08 |
| GOBAR_AA35548 | 0.2 | 0.023 | -2.249270897 | 0.049827407 | 0.009614578 |
| GOBAR_AA24900 | 1.583 | 0.266 | -2.26496174 | 0.010034681 | 0.001304372 |
| GOBAR_AA35770 | 7.283 | 1.586 | -2.271533649 | 0.000229721 | 1.57E-05 |
| GOBAR_AA33408 | 5.803 | 1.346 | -2.286658913 | 2.15E-10 | 4.83E-12 |
| GOBAR_AA02489 | 25.37 | 5.86 | -2.287775084 | 3.60E-14 | 5.44E-16 |
| GOBAR_AA09751 | 1.576 | 0.33 | -2.309295489 | 2.97E-05 | 1.56E-06 |
| GOBAR_AA08879 | 3.146 | 0.586 | -2.31169981 | 0.006239681 | 0.000728164 |
| GOBAR_AA29350 | 4.696 | 0.9 | -2.316367085 | 0.000612541 | 4.86E-05 |
| GOBAR_AA31136 | 2.036 | 0.433 | -2.355283214 | 9.56E-07 | 3.65E-08 |
| GOBAR_AA04207 | 1.076 | 0.086 | -2.372675286 | 0.037871133 | 0.006786808 |
| GOBAR_AA14053 | 0.356 | 0 | -2.385757225 | 0.0482719 | 0.009211247 |
| GOBAR_AA38655 | 0.45 | 0.033 | -2.391689418 | 0.037267099 | 0.006642691 |
| GOBAR_AA23957 | 0.673 | 0.113 | -2.392137348 | 0.0016567 | 0.000154429 |
| GOBAR_AA08892 | 1.356 | 0.163 | -2.405465353 | 0.018197406 | 0.002714446 |
| GOBAR_AA09730 | 9.063 | 1.643 | -2.454132743 | 9.22E-07 | 3.52E-08 |
| GOBAR_AA26633 | 16.116 | 2.056 | -2.467174042 | 0.008850582 | 0.001116197 |
| GOBAR_AA17353 | 123.99 | 24.766 | -2.487331524 | 2.55E-14 | 3.81E-16 |
| GOBAR_AA36042 | 26.68 | 6.103 | -2.491839581 | 4.71E-45 | 1.59E-47 |
| GOBAR_AA22524 | 12.253 | 2.353 | -2.492895947 | 3.08E-08 | 9.11E-10 |
| GOBAR_AA01907 | 5.053 | 0.983 | -2.512281924 | 4.61E-16 | 5.96E-18 |
| GOBAR_AA36705 | 10.85 | 2.293 | -2.51690966 | 9.83E-31 | 5.42E-33 |
| GOBAR_AA26026 | 0.966 | 0.066 | -2.521938516 | 0.022629546 | 0.003559256 |
| GOBAR_AA14381 | 8.69 | 1.88 | -2.532437219 | 1.21E-28 | 7.40E-31 |
| GOBAR_AA17400 | 57.686 | 11.29 | -2.556116566 | 8.06E-13 | 1.40E-14 |
| GOBAR_AA28251 | 0.82 | 0.08 | -2.576392327 | 0.011189142 | 0.001487081 |
| GOBAR_AA06604 | 149.13 | 26.92 | -2.594597507 | 1.34E-57 | 3.05E-60 |
| GOBAR_AA14791 | 12.52 | 2.206 | -2.595672076 | 4.37E-08 | 1.32E-09 |
| GOBAR_AA34163 | 2.333 | 0.666 | -2.606373387 | 2.80E-11 | 5.69E-13 |
| GOBAR_AA07239 | 41.95 | 7.293 | -2.636319724 | 7.89E-16 | 1.05E-17 |
| GOBAR_AA15055 | 1.43 | 0 | -2.732167209 | 0.018041117 | 0.002685922 |
| GOBAR_AA04233 | 79.12 | 12.596 | -2.747918959 | 6.15E-31 | 3.34E-33 |
| GOBAR_AA12516 | 12.906 | 2.046 | -2.748296303 | 5.14E-12 | 9.78E-14 |
| GOBAR_AA02034 | 102.573 | 15.293 | -2.828417532 | 9.14E-11 | 1.98E-12 |
| GOBAR_AA12958 | 17.086 | 2.536 | -2.849271425 | 4.97E-09 | 1.33E-10 |
| GOBAR_AA25679 | 6.23 | 0.96 | -2.853852984 | 3.57E-12 | 6.66E-14 |
| GOBAR_AA13677 | 29.926 | 4.686 | -2.874781047 | 8.08E-55 | 1.98E-57 |
| GOBAR_AA38757 | 4.69 | 0.626 | -2.879137027 | 6.00E-07 | 2.19E-08 |
| GOBAR_AA18783 | 18.573 | 1.313 | -2.887302098 | 0.005328865 | 0.000606462 |
| GOBAR_AA10353 | 1.9 | 0.236 | -2.922354777 | 1.38E-06 | 5.45E-08 |
| GOBAR_AA09251 | 0.703 | 0 | -2.941632633 | 0.009397202 | 0.001203593 |
| GOBAR_AA39007 | 58.04 | 8.403 | -2.955829201 | 5.89E-38 | 2.55E-40 |
| GOBAR_AA04563 | 22.696 | 3.053 | -2.991153103 | 3.22E-13 | 5.36E-15 |
| GOBAR_AA29014 | 2.263 | 0.326 | -3.009182677 | 3.76E-15 | 5.28E-17 |
| GOBAR_AA08470 | 1.1 | 0.11 | -3.019589925 | 2.59E-05 | 1.34E-06 |
| GOBAR_AA07777 | 249.636 | 36.46 | -3.047375724 | 3.50E-114 | 2.53E-117 |
| GOBAR_AA07044 | 1.976 | 0 | -3.108644372 | 0.004809441 | 0.000533743 |
| GOBAR_AA11600 | 47.743 | 6.11 | -3.13125157 | 1.94E-20 | 1.81E-22 |
| GOBAR_AA29066 | 98.38 | 14.743 | -3.160335841 | 7.73E-28 | 4.87E-30 |
| GOBAR_AA05496 | 4.186 | 0.243 | -3.183803422 | 0.000678471 | 5.45E-05 |
| GOBAR_AA35470 | 99.596 | 5.28 | -3.228510191 | 0.000734148 | 5.99E-05 |
| GOBAR_AA30819 | 4.643 | 0.543 | -3.250996566 | 1.32E-28 | 8.14E-31 |
| GOBAR_AA02317 | 1.77 | 0.163 | -3.259344057 | 2.90E-05 | 1.51E-06 |
| GOBAR_AA33158 | 8.066 | 0.83 | -3.276714754 | 2.67E-09 | 6.93E-11 |
| GOBAR_AA07175 | 2.863 | 0.326 | -3.29301887 | 3.50E-20 | 3.38E-22 |
| GOBAR_AA07466 | 1.97 | 0.036 | -3.395903303 | 0.001368209 | 0.000123308 |
| GOBAR_AA37328 | 120.46 | 0.113 | -3.41336168 | 0.001927558 | 0.000183407 |
| GOBAR_AA01941 | 10.256 | 0 | -3.474129475 | 0.0015021 | 0.000137892 |
| GOBAR_AA12643 | 7.993 | 0.816 | -3.504711051 | 4.73E-35 | 2.30E-37 |
| GOBAR_AA12520 | 0.636 | 0.016 | -3.576906544 | 0.000184267 | 1.22E-05 |
| GOBAR_AA28301 | 3.383 | 0.036 | -3.619316927 | 0.00048519 | 3.73E-05 |
| GOBAR_AA14697 | 21.506 | 1.23 | -3.751616697 | 2.19E-08 | 6.36E-10 |
| GOBAR_AA33670 | 2.49 | 0.103 | -3.833552302 | 1.81E-06 | 7.34E-08 |
| GOBAR_AA36252 | 0.84 | 0 | -3.834596427 | 0.000164316 | 1.06E-05 |
| GOBAR_AA03659 | 102.493 | 7.806 | -3.852839944 | 1.28E-89 | 1.48E-92 |
| GOBAR_AA14083 | 3.733 | 0.173 | -3.888090414 | 9.46E-08 | 3.01E-09 |
| GOBAR_AA35700 | 27.943 | 1.57 | -3.917732189 | 1.77E-10 | 3.94E-12 |
| GOBAR_AA01086 | 1.46 | 0.02 | -3.923986371 | 4.28E-05 | 2.35E-06 |
| GOBAR_AA38817 | 267.06 | 0.41 | -3.940651139 | 0.000190466 | 1.26E-05 |
| GOBAR_AA10203 | 6.213 | 0.416 | -4.038691792 | 6.21E-24 | 4.68E-26 |
| GOBAR_AA01768 | 37.523 | 2.54 | -4.046865323 | 8.68E-37 | 4.01E-39 |
| GOBAR_AA09859 | 10.336 | 0.64 | -4.072949248 | 2.42E-25 | 1.66E-27 |
| GOBAR_AA10909 | 6.9 | 0.35 | -4.158372824 | 3.14E-15 | 4.37E-17 |
| GOBAR_AA03206 | 19.33 | 1.096 | -4.180778716 | 1.36E-23 | 1.05E-25 |
| GOBAR_AA13312 | 10.723 | 0.323 | -4.257191283 | 2.01E-08 | 5.79E-10 |
| GOBAR_AA06903 | 25.56 | 0.99 | -4.303706196 | 4.97E-11 | 1.05E-12 |
| GOBAR_AA32600 | 239.203 | 10.923 | -4.442660169 | 1.07E-74 | 1.61E-77 |
| GOBAR_AA21005 | 1.673 | 0.053 | -4.449000922 | 4.79E-11 | 1.01E-12 |
| GOBAR_AA33731 | 3.143 | 0.19 | -4.66445159 | 1.82E-24 | 1.33E-26 |
| GOBAR_AA27390 | 11.303 | 0.436 | -4.685813246 | 4.05E-32 | 2.15E-34 |
| GOBAR_AA32211 | 109.953 | 4.336 | -4.755523461 | 4.31E-50 | 1.31E-52 |
| GOBAR_AA17615 | 655.546 | 24.42 | -4.872433545 | 2.03E-75 | 2.94E-78 |
| GOBAR_AA26327 | 20.353 | 0.77 | -4.883391585 | 2.11E-43 | 7.76E-46 |
| GOBAR_AA30876 | 16.66 | 0.563 | -4.951014894 | 3.23E-50 | 9.60E-53 |
| GOBAR_AA36953 | 0.896 | 0 | -4.961428427 | 2.60E-08 | 7.61E-10 |
| GOBAR_AA06434 | 37.286 | 1.163 | -5.004959899 | 1.65E-77 | 2.33E-80 |
| GOBAR_AA00612 | 48.503 | 1.593 | -5.00501939 | 1.42E-50 | 4.08E-53 |
| GOBAR_AA04822 | 17.3 | 0.44 | -5.093175218 | 5.92E-22 | 5.01E-24 |
| GOBAR_AA05165 | 24.783 | 0.703 | -5.160851123 | 2.23E-50 | 6.50E-53 |
| GOBAR_AA00006 | 6.076 | 0.153 | -5.209309766 | 2.08E-20 | 1.95E-22 |
| GOBAR_AA31454 | 24.233 | 0.196 | -5.484249796 | 3.42E-13 | 5.71E-15 |
| GOBAR_AA28360 | 5675.503 | 6.256 | -5.561438842 | 3.15E-09 | 8.23E-11 |
| GOBAR_AA08471 | 20.706 | 0.193 | -5.622944204 | 1.80E-15 | 2.45E-17 |
| GOBAR_AA31329 | 2.07 | 0 | -6.222623074 | 7.43E-15 | 1.06E-16 |
| GOBAR_AA19621 | 13.87 | 0.186 | -6.361603731 | 8.59E-68 | 1.51E-70 |
| GOBAR_AA39823 | 138.096 | 2.373 | -6.726091765 | 2.16E-172 | 2.49E-176 |
| GOBAR_AA28220 | 200.55 | 1.433 | -6.785246077 | 4.17E-41 | 1.61E-43 |
| GOBAR_AA03072 | 180.686 | 0.48 | -6.953101948 | 1.02E-20 | 9.30E-23 |
| GOBAR_AA11009 | 10.423 | 0.013 | -7.440898491 | 3.36E-29 | 1.99E-31 |
