# Supplementary material for: Integrated Methylome and Transcriptome Analysis Widen the Knowledge of Cytoplasmic Male Sterility in Cotton (Gossypium barbadense L.)
Source: Front Plant Sci. 2022 Apr 27;13:770098. doi: 10.3389/fpls.2022.770098 (PMC9093596; doi:10.3389/fpls.2022.770098)
Supplement: Supplementary file 9 [file Table_9.DOCX]

**Table S9** Up-regulated and down-regulated genes in the hyper-DEGs（ZA:07-113A,ZB:07-113B）

| Gene ID | ZB FPKM | ZA FPKM | log2(ZA/ZB) | Qvalue(ZB-vs-ZA) | Pvalue(ZB-vs-ZA) |
| --- | --- | --- | --- | --- | --- |
| GOBAR_AA37964 | 0 | 1.843 | 5.518815837 | 9.75E-11 | 2.12E-12 |
| GOBAR_AA17575 | 0.006 | 0.716 | 3.994869881 | 2.15E-05 | 1.08E-06 |
| GOBAR_AA14608 | 0.106 | 2.7 | 3.69900811 | 9.02E-07 | 3.43E-08 |
| GOBAR_AA35206 | 0 | 0.383 | 3.424040732 | 0.00122571 | 0.000108766 |
| GOBAR_AA09793 | 0.033 | 0.94 | 3.375338312 | 0.00019616 | 1.31E-05 |
| GOBAR_AA03184 | 0.07 | 1.17 | 3.133520154 | 4.41E-05 | 2.43E-06 |
| GOBAR_AA05849 | 0.036 | 0.716 | 3.090720943 | 0.000498044 | 3.83E-05 |
| GOBAR_AA11474 | 0.073 | 1.1 | 3.040900084 | 0.000196 | 1.31E-05 |
| GOBAR_AA35205 | 0 | 0.273 | 2.971158793 | 0.007872635 | 0.000966617 |
| GOBAR_AA01435 | 0.22 | 2.856 | 2.93800453 | 0.000186105 | 1.23E-05 |
| GOBAR_AA36907 | 0.086 | 1.093 | 2.912345808 | 6.65E-05 | 3.84E-06 |
| GOBAR_AA37858 | 0 | 0.27 | 2.866387622 | 0.011754221 | 0.001579837 |
| GOBAR_AA16537 | 0.84 | 8.773 | 2.75618209 | 7.43E-05 | 4.35E-06 |
| GOBAR_AA32399 | 8.916 | 71.006 | 2.677766014 | 7.43E-12 | 1.44E-13 |
| GOBAR_AA40136 | 0 | 0.166 | 2.437416733 | 0.041953174 | 0.007710048 |
| GOBAR_AA33915 | 0.01 | 0.31 | 2.414022708 | 0.040514655 | 0.007374093 |
| GOBAR_AA18610 | 0.123 | 0.836 | 2.330400501 | 0.002277106 | 0.000222587 |
| GOBAR_AA16690 | 0.67 | 4.24 | 2.280318182 | 1.09E-07 | 3.51E-09 |
| GOBAR_AA36954 | 2.09 | 10.793 | 2.20620869 | 1.01E-05 | 4.75E-07 |
| GOBAR_AA18329 | 0.616 | 3.57 | 2.173071347 | 4.30E-05 | 2.36E-06 |
| GOBAR_AA33473 | 0.186 | 1.116 | 2.136059172 | 0.000555365 | 4.34E-05 |
| GOBAR_AA20804 | 0.17 | 1.19 | 2.127665999 | 0.036381965 | 0.006433762 |
| GOBAR_AA31127 | 1.38 | 9.513 | 2.101147659 | 0.017644741 | 0.002608106 |
| GOBAR_AA04804 | 0.73 | 5.016 | 2.096503585 | 0.018276895 | 0.002730527 |
| GOBAR_AA38190 | 0.306 | 2.446 | 2.084873243 | 0.04610672 | 0.00868355 |
| GOBAR_AA15039 | 0.973 | 5.573 | 2.037727788 | 0.002679054 | 0.000268455 |
| GOBAR_AA10808 | 3.066 | 15.25 | 2.016865215 | 0.000139185 | 8.78E-06 |
| GOBAR_AA09417 | 0.643 | 2.786 | 1.92514762 | 0.000498829 | 3.84E-05 |
| GOBAR_AA16458 | 1.476 | 6.153 | 1.797784714 | 7.04E-08 | 2.20E-09 |
| GOBAR_AA16558 | 0.526 | 2.383 | 1.784815162 | 0.005023183 | 0.000562404 |
| GOBAR_AA01728 | 2.57 | 9.986 | 1.657639866 | 1.14E-06 | 4.43E-08 |
| GOBAR_AA16977 | 2.416 | 9.353 | 1.632574809 | 0.001885819 | 0.000178891 |
| GOBAR_AA33954 | 6.833 | 24.633 | 1.605225638 | 8.07E-05 | 4.76E-06 |
| GOBAR_AA22924 | 0.313 | 1.263 | 1.604590737 | 0.019558713 | 0.00297344 |
| GOBAR_AA35657 | 5.153 | 19.086 | 1.604168525 | 2.05E-10 | 4.59E-12 |
| GOBAR_AA02684 | 0.71 | 2.62 | 1.538220709 | 0.03269129 | 0.005637569 |
| GOBAR_AA24237 | 0.616 | 2.3 | 1.504367768 | 0.023576722 | 0.003745603 |
| GOBAR_AA38102 | 1.076 | 3.503 | 1.457089318 | 0.001497152 | 0.000137293 |
| GOBAR_AA11323 | 2.58 | 8.276 | 1.364981697 | 0.010085479 | 0.001313889 |
| GOBAR_AA36162 | 5.256 | 15.986 | 1.362313527 | 7.33E-09 | 2.02E-10 |
| GOBAR_AA34881 | 9.25 | 27.926 | 1.346199841 | 2.44E-06 | 1.02E-07 |
| GOBAR_AA01934 | 2.42 | 6.326 | 1.333813036 | 0.045761785 | 0.00860008 |
| GOBAR_AA30676 | 4.166 | 12.43 | 1.320548674 | 2.74E-07 | 9.41E-09 |
| GOBAR_AA38637 | 0.796 | 2.38 | 1.311756919 | 0.01263484 | 0.00172776 |
| GOBAR_AA40129 | 4.03 | 11.74 | 1.290842731 | 0.000125553 | 7.82E-06 |
| GOBAR_AA29569 | 30.31 | 87.273 | 1.287083856 | 1.12E-05 | 5.35E-07 |
| GOBAR_AA06516 | 39.673 | 116.11 | 1.283830866 | 3.22E-07 | 1.12E-08 |
| GOBAR_AA37962 | 4.243 | 12.373 | 1.276116624 | 1.44E-05 | 7.06E-07 |
| GOBAR_AA29215 | 1.933 | 5.75 | 1.273665406 | 0.017991569 | 0.002675427 |
| GOBAR_AA15597 | 1.486 | 4.46 | 1.270309401 | 0.010261724 | 0.001341888 |
| GOBAR_AA38442 | 0.813 | 2.326 | 1.26566052 | 0.001111127 | 9.73E-05 |
| GOBAR_AA06355 | 1.883 | 5.613 | 1.255366831 | 0.030858533 | 0.005251985 |
| GOBAR_AA33754 | 1.136 | 3.253 | 1.243091605 | 0.01015353 | 0.001324514 |
| GOBAR_AA29865 | 10.346 | 29.456 | 1.241539908 | 0.000852096 | 7.16E-05 |
| GOBAR_AA14260 | 0.65 | 1.946 | 1.24084143 | 0.04836607 | 0.009233408 |
| GOBAR_AA18473 | 3.543 | 9.313 | 1.237718773 | 0.005526244 | 0.000633413 |
| GOBAR_AA31391 | 25.096 | 51.836 | 1.227511855 | 1.26E-05 | 6.09E-07 |
| GOBAR_AA23939 | 41.273 | 113.606 | 1.209756778 | 1.57E-10 | 3.47E-12 |
| GOBAR_AA03061 | 1.896 | 5.273 | 1.192763182 | 0.000600866 | 4.75E-05 |
| GOBAR_AA36277 | 1.653 | 4.8 | 1.191137268 | 0.033131295 | 0.00573833 |
| GOBAR_AA39544 | 1.633 | 4.62 | 1.173910136 | 0.044437303 | 0.008288271 |
| GOBAR_AA20123 | 0.78 | 2.1 | 1.162140326 | 0.023607991 | 0.003753239 |
| GOBAR_AA34598 | 1.053 | 2.863 | 1.161981405 | 0.014734339 | 0.002094873 |
| GOBAR_AA30274 | 3.936 | 10.303 | 1.151496869 | 0.00108178 | 9.42E-05 |
| GOBAR_AA15515 | 2.226 | 5.896 | 1.149896199 | 0.001903955 | 0.000180831 |
| GOBAR_AA16459 | 1.123 | 2.956 | 1.143172207 | 0.018358804 | 0.002744428 |
| GOBAR_AA26422 | 9.086 | 23.553 | 1.130903794 | 0.003115226 | 0.00031954 |
| GOBAR_AA06761 | 1.283 | 3.36 | 1.127695587 | 0.006628468 | 0.000781385 |
| GOBAR_AA13934 | 0.673 | 1.933 | 1.1230564 | 0.036053783 | 0.006360104 |
| GOBAR_AA16498 | 16.73 | 44.393 | 1.100898718 | 0.005067526 | 0.000568979 |
| GOBAR_AA14559 | 1.776 | 4.33 | 1.095669419 | 1.44E-05 | 7.02E-07 |
| GOBAR_AA27644 | 1.563 | 4.396 | 1.09091245 | 0.000525164 | 4.07E-05 |
| GOBAR_AA03907 | 7.116 | 16.726 | 1.086740736 | 0.000481897 | 3.70E-05 |
| GOBAR_AA26037 | 6.52 | 16.56 | 1.083032138 | 0.006162649 | 0.000717928 |
| GOBAR_AA31929 | 6.393 | 16.726 | 1.082879171 | 0.001038409 | 8.97E-05 |
| GOBAR_AA02806 | 6.393 | 15.94 | 1.082321259 | 1.33E-05 | 6.46E-07 |
| GOBAR_AA19743 | 5.066 | 12.656 | 1.070053568 | 3.76E-05 | 2.03E-06 |
| GOBAR_AA07195 | 40.726 | 99.423 | 1.052516296 | 5.75E-08 | 1.77E-09 |
| GOBAR_AA34517 | 1.246 | 3.096 | 1.047735328 | 0.018861617 | 0.002834228 |
| GOBAR_AA02628 | 88.42 | 213.94 | 1.040426783 | 5.78E-08 | 1.78E-09 |
| GOBAR_AA08878 | 24.496 | 59.44 | 1.036298675 | 1.59E-05 | 7.83E-07 |
| GOBAR_AA08022 | 5.403 | 10.856 | 1.030480363 | 0.000127256 | 7.94E-06 |
| GOBAR_AA07335 | 420.376 | 956.006 | 1.024678426 | 0.000769395 | 6.33E-05 |
| GOBAR_AA20393 | 2.776 | 6.636 | 1.012559553 | 0.009041158 | 0.001149112 |
| GOBAR_AA27930 | 10.893 | 25.426 | 1.010358491 | 3.14E-06 | 1.34E-07 |
| GOBAR_AA33669 | 42.073 | 100.543 | 1.005255739 | 0.000330058 | 2.39E-05 |
| GOBAR_AA21999 | 8.703 | 20.443 | 0.990551547 | 0.000215172 | 1.46E-05 |
| GOBAR_AA20757 | 4.093 | 9.703 | 0.972999271 | 0.000702421 | 5.70E-05 |
| GOBAR_AA34378 | 4.053 | 9.18 | 0.96914879 | 0.000231728 | 1.59E-05 |
| GOBAR_AA03855 | 6.27 | 14.19 | 0.966835075 | 0.000873103 | 7.37E-05 |
| GOBAR_AA25791 | 6.256 | 14.206 | 0.950606864 | 1.04E-05 | 4.90E-07 |
| GOBAR_AA08940 | 12.82 | 29.006 | 0.946478273 | 0.003728446 | 0.000395365 |
| GOBAR_AA32479 | 24.876 | 58.44 | 0.942006239 | 0.022667982 | 0.003567266 |
| GOBAR_AA26825 | 5.05 | 11.31 | 0.936236904 | 0.003577458 | 0.000376047 |
| GOBAR_AA26118 | 2.166 | 4.913 | 0.930797551 | 0.048828562 | 0.009360049 |
| GOBAR_AA00789 | 4.506 | 10.276 | 0.928199316 | 0.009600344 | 0.001236741 |
| GOBAR_AA34852 | 6.41 | 14.546 | 0.927903141 | 0.013640709 | 0.001902741 |
| GOBAR_AA38890 | 17.65 | 39.23 | 0.925872338 | 0.000627142 | 4.99E-05 |
| GOBAR_AA01087 | 9.876 | 21.32 | 0.920947029 | 9.92E-06 | 4.67E-07 |
| GOBAR_AA04919 | 10.863 | 23.763 | 0.892330215 | 0.001136611 | 9.99E-05 |
| GOBAR_AA08402 | 16.153 | 34.743 | 0.891393137 | 0.000850586 | 7.14E-05 |
| GOBAR_AA28344 | 5.936 | 13.273 | 0.886493132 | 0.000496501 | 3.82E-05 |
| GOBAR_AA39746 | 9.016 | 17.02 | 0.880255447 | 3.40E-05 | 1.81E-06 |
| GOBAR_AA12266 | 29.823 | 64.46 | 0.88007132 | 0.007316605 | 0.000881949 |
| GOBAR_AA11984 | 16.13 | 34.636 | 0.877675737 | 0.000461217 | 3.51E-05 |
| GOBAR_AA01088 | 11.793 | 25.753 | 0.873596424 | 0.006059332 | 0.000703617 |
| GOBAR_AA07021 | 1.563 | 3.25 | 0.868553147 | 0.008574762 | 0.001073982 |
| GOBAR_AA32900 | 3.356 | 7.17 | 0.854224955 | 0.043032142 | 0.007961549 |
| GOBAR_AA30436 | 27.496 | 58.493 | 0.853231298 | 0.000213685 | 1.44E-05 |
| GOBAR_AA34708 | 7.403 | 14.533 | 0.84725195 | 0.003767142 | 0.000400447 |
| GOBAR_AA40200 | 6.526 | 13.833 | 0.844598592 | 0.005172729 | 0.000584825 |
| GOBAR_AA21104 | 1.08 | 2.273 | 0.844154461 | 0.009006822 | 0.001142926 |
| GOBAR_AA03024 | 14.016 | 30.34 | 0.843957851 | 0.000365662 | 2.69E-05 |
| GOBAR_AA21483 | 9.97 | 21.02 | 0.839050751 | 0.003427113 | 0.000357868 |
| GOBAR_AA12378 | 2.013 | 4.6 | 0.838842184 | 0.011076288 | 0.001468882 |
| GOBAR_AA21872 | 4.043 | 8.803 | 0.824783443 | 0.000260961 | 1.83E-05 |
| GOBAR_AA19835 | 13.06 | 27.246 | 0.824165145 | 0.013318996 | 0.001843353 |
| GOBAR_AA39857 | 56.283 | 118.03 | 0.806005625 | 0.002115908 | 0.000204201 |
| GOBAR_AA27905 | 4.216 | 8.61 | 0.805298959 | 0.000504474 | 3.89E-05 |
| GOBAR_AA16836 | 20.77 | 44.46 | 0.804488645 | 0.000146013 | 9.28E-06 |
| GOBAR_AA27552 | 44.013 | 88.296 | 0.797667279 | 0.00015901 | 1.02E-05 |
| GOBAR_AA17379 | 15.986 | 32.126 | 0.797161377 | 0.001351747 | 0.000121551 |
| GOBAR_AA40034 | 4.683 | 9.856 | 0.791171879 | 0.000154229 | 9.86E-06 |
| GOBAR_AA06042 | 8.79 | 17.493 | 0.78431926 | 0.005329038 | 0.000606808 |
| GOBAR_AA03008 | 3.466 | 7.256 | 0.780422146 | 0.00163811 | 0.000152527 |
| GOBAR_AA17865 | 6.013 | 11.906 | 0.774963628 | 0.000175698 | 1.15E-05 |
| GOBAR_AA33383 | 8.43 | 16.866 | 0.771842667 | 0.036072675 | 0.006366223 |
| GOBAR_AA31619 | 8.583 | 17.303 | 0.768645932 | 0.047237962 | 0.008949819 |
| GOBAR_AA20174 | 130.803 | 254.743 | 0.763201728 | 0.000232259 | 1.59E-05 |
| GOBAR_AA05660 | 4.053 | 8.233 | 0.760655301 | 0.016965023 | 0.002486511 |
| GOBAR_AA27774 | 10.106 | 20.033 | 0.755029685 | 0.009758531 | 0.001260584 |
| GOBAR_AA14719 | 6 | 12.13 | 0.752501897 | 0.039770768 | 0.007213399 |
| GOBAR_AA35079 | 24.806 | 49.503 | 0.749254327 | 0.002220771 | 0.000215925 |
| GOBAR_AA21656 | 9.823 | 19.166 | 0.727984611 | 0.00010827 | 6.62E-06 |
| GOBAR_AA34512 | 26.056 | 50.846 | 0.726707261 | 0.004073683 | 0.000439387 |
| GOBAR_AA05687 | 6.4 | 12.08 | 0.721820659 | 0.032276906 | 0.005547462 |
| GOBAR_AA40176 | 8.076 | 15.593 | 0.720465812 | 0.006402642 | 0.000749031 |
| GOBAR_AA23414 | 12.516 | 24.196 | 0.717700009 | 0.000204001 | 1.37E-05 |
| GOBAR_AA24602 | 42.843 | 82.496 | 0.713016497 | 9.05E-06 | 4.21E-07 |
| GOBAR_AA09696 | 3.613 | 6.936 | 0.705910152 | 0.014162184 | 0.00199389 |
| GOBAR_AA02217 | 3.456 | 6.623 | 0.700004822 | 0.013755032 | 0.001921866 |
| GOBAR_AA38284 | 132.476 | 250.45 | 0.696024857 | 0.022401229 | 0.00351558 |
| GOBAR_AA10732 | 9.523 | 18.07 | 0.694671321 | 0.002716204 | 0.000273093 |
| GOBAR_AA19030 | 146.053 | 275.516 | 0.687931516 | 2.99E-05 | 1.57E-06 |
| GOBAR_AA10155 | 31.773 | 60.32 | 0.686363605 | 0.037267099 | 0.006643036 |
| GOBAR_AA32819 | 4.763 | 9.046 | 0.685436668 | 0.022241955 | 0.003484802 |
| GOBAR_AA26663 | 34.566 | 65.346 | 0.682270263 | 0.000102467 | 6.22E-06 |
| GOBAR_AA24575 | 28.29 | 53.076 | 0.681119926 | 0.002333362 | 0.000228962 |
| GOBAR_AA01058 | 10.233 | 19.143 | 0.67363679 | 0.01418113 | 0.001997787 |
| GOBAR_AA14757 | 16.79 | 31.35 | 0.671501753 | 0.000397751 | 2.97E-05 |
| GOBAR_AA26013 | 26.863 | 50.426 | 0.670619363 | 8.39E-05 | 4.97E-06 |
| GOBAR_AA34291 | 9.093 | 17.04 | 0.668946221 | 9.66E-05 | 5.82E-06 |
| GOBAR_AA03160 | 10.036 | 18.78 | 0.666847585 | 0.028168357 | 0.004684284 |
| GOBAR_AA27553 | 12.07 | 22.246 | 0.656204021 | 0.008423109 | 0.001050121 |
| GOBAR_AA19321 | 20.853 | 39.146 | 0.655151541 | 0.003792614 | 0.000403593 |
| GOBAR_AA37634 | 20.59 | 37.963 | 0.647855531 | 0.002225898 | 0.000216874 |
| GOBAR_AA38946 | 11.853 | 21.54 | 0.645109911 | 0.005594261 | 0.000642341 |
| GOBAR_AA24721 | 7.333 | 13.61 | 0.642512971 | 0.034966893 | 0.006116858 |
| GOBAR_AA12495 | 27.78 | 52.413 | 0.642263247 | 0.000184267 | 1.22E-05 |
| GOBAR_AA08721 | 9.74 | 17.856 | 0.642216437 | 0.007982065 | 0.000984068 |
| GOBAR_AA22774 | 19.186 | 35.26 | 0.636911306 | 0.021061902 | 0.003253864 |
| GOBAR_AA02371 | 13.3 | 24.423 | 0.635132561 | 0.027572646 | 0.004559733 |
| GOBAR_AA14851 | 23.636 | 46.873 | 0.633980992 | 0.013350095 | 0.001849477 |
| GOBAR_AA12307 | 7.796 | 14.196 | 0.62406126 | 0.003970667 | 0.000426179 |
| GOBAR_AA30446 | 2.146 | 3.866 | 0.622283293 | 0.036643926 | 0.006493847 |
| GOBAR_AA07060 | 7.473 | 13.436 | 0.615457293 | 0.004601585 | 0.0005066 |
| GOBAR_AA22724 | 7.33 | 13.136 | 0.610564735 | 0.043362093 | 0.008033868 |
| GOBAR_AA34623 | 13.206 | 24.2 | 0.605619311 | 0.02399796 | 0.003827168 |
| GOBAR_AA19677 | 13.11 | 23.313 | 0.60040699 | 0.002993011 | 0.000304988 |
| GOBAR_AA05227 | 43.99 | 77.49 | 0.586630811 | 0.003301747 | 0.000342297 |
| GOBAR_AA15608 | 2.53 | 5.516 | 0.580505992 | 0.029559452 | 0.004964287 |
| GOBAR_AA24725 | 113.503 | 196.52 | 0.570510464 | 0.017623497 | 0.002603766 |
| GOBAR_AA14378 | 12.69 | 21.69 | 0.535722696 | 0.009730286 | 0.001255811 |
| GOBAR_AA27923 | 15.013 | 25.373 | 0.530941711 | 0.023072699 | 0.003648284 |
| GOBAR_AA03669 | 35.69 | 59.49 | 0.528847511 | 0.014073537 | 0.001976806 |
| GOBAR_AA34080 | 66.583 | 112.756 | 0.528389256 | 0.005380038 | 0.000613858 |
| GOBAR_AA02810 | 25.05 | 41.64 | 0.528136995 | 0.031790393 | 0.005449152 |
| GOBAR_AA10234 | 10.556 | 17.736 | 0.518030688 | 0.030795591 | 0.005237387 |
| GOBAR_AA23224 | 34.38 | 57.583 | 0.514393753 | 0.047648015 | 0.009049531 |
| GOBAR_AA00457 | 35.123 | 58.546 | 0.511893466 | 0.041057185 | 0.007496533 |
| GOBAR_AA06004 | 290.216 | 479.676 | 0.498942938 | 0.000357963 | 2.62E-05 |
| GOBAR_AA36458 | 71.233 | 110.48 | 0.497079263 | 0.009474542 | 0.001216735 |
| GOBAR_AA22234 | 118.076 | 194.96 | 0.495774294 | 0.025580595 | 0.004139417 |
| GOBAR_AA12203 | 22.383 | 36.673 | 0.474935974 | 0.034199797 | 0.005951055 |
| GOBAR_AA34363 | 16.646 | 26.696 | 0.472105875 | 0.035852469 | 0.006319413 |
| GOBAR_AA33041 | 41.56 | 67.743 | 0.47157208 | 0.023829619 | 0.00379688 |
| GOBAR_AA14980 | 14.703 | 24.753 | 0.469455514 | 0.047898309 | 0.009105369 |
| GOBAR_AA12880 | 7.786 | 12.64 | 0.465996034 | 0.032652215 | 0.005628944 |
| GOBAR_AA32000 | 20.006 | 32.21 | 0.461002934 | 0.03563247 | 0.006268285 |
| GOBAR_AA32398 | 65.61 | 105.22 | 0.460642396 | 0.042449651 | 0.007831709 |
| GOBAR_AA26227 | 16.296 | 25.746 | 0.444849036 | 0.026392515 | 0.004307395 |
| GOBAR_AA08111 | 48.74 | 77.59 | 0.442278567 | 0.02708288 | 0.0044584 |
| GOBAR_AA11484 | 7.193 | 11.036 | 0.42440909 | 0.037119488 | 0.006604929 |
| GOBAR_AA32717 | 40.89 | 64.366 | 0.422753978 | 0.026534753 | 0.004337507 |
| GOBAR_AA35141 | 30.623 | 47.966 | 0.416231654 | 0.041063168 | 0.007501184 |
| GOBAR_AA00785 | 11.376 | 19.586 | 0.415349548 | 0.048367389 | 0.009235057 |
| GOBAR_AA32857 | 82.126 | 119.863 | 0.394455326 | 0.045587107 | 0.008559352 |
| GOBAR_AA22441 | 29.18 | 43.44 | 0.349224343 | 0.049654954 | 0.009562656 |
| GOBAR_AA38884 | 17.923 | 16.58 | -0.371007626 | 0.038696378 | 0.006971585 |
| GOBAR_AA08033 | 61.006 | 55.403 | -0.3735477 | 0.044757721 | 0.008362256 |
| GOBAR_AA03780 | 21.013 | 18.776 | -0.393411935 | 0.04262923 | 0.007875922 |
| GOBAR_AA31679 | 18.193 | 16.19 | -0.397675376 | 0.049655514 | 0.009564198 |
| GOBAR_AA08453 | 17.3 | 14.73 | -0.424180054 | 0.030621876 | 0.00519667 |
| GOBAR_AA12023 | 43.286 | 37.683 | -0.424309491 | 0.033884601 | 0.005887399 |
| GOBAR_AA31839 | 45.166 | 38.643 | -0.443692173 | 0.04079083 | 0.007431404 |
| GOBAR_AA27946 | 22.943 | 19.643 | -0.444311565 | 0.034404342 | 0.005991617 |
| GOBAR_AA19097 | 17.626 | 15.176 | -0.44692266 | 0.008354419 | 0.001037937 |
| GOBAR_AA25437 | 58.21 | 50.27 | -0.447136241 | 0.035724041 | 0.006292649 |
| GOBAR_AA38383 | 36.61 | 31.313 | -0.452321104 | 0.019685459 | 0.002995789 |
| GOBAR_AA05607 | 20 | 17.196 | -0.457831221 | 0.005345016 | 0.000609244 |
| GOBAR_AA07935 | 32.743 | 30.783 | -0.459912167 | 0.025915901 | 0.004210894 |
| GOBAR_AA26084 | 15.636 | 14.01 | -0.482193531 | 0.03428073 | 0.005967119 |
| GOBAR_AA19299 | 21.616 | 18.053 | -0.489242141 | 0.01527733 | 0.002190167 |
| GOBAR_AA07184 | 40.053 | 33.223 | -0.491853414 | 0.023236956 | 0.003676942 |
| GOBAR_AA40141 | 50.423 | 43.026 | -0.492828137 | 0.034706739 | 0.006058315 |
| GOBAR_AA05202 | 56.033 | 46.17 | -0.495710526 | 0.01062299 | 0.00139496 |
| GOBAR_AA02776 | 38.46 | 31.7 | -0.498358018 | 0.007828193 | 0.000958766 |
| GOBAR_AA27050 | 11.573 | 9.816 | -0.499153162 | 0.006106315 | 0.000710454 |
| GOBAR_AA05739 | 10.6 | 8.613 | -0.511381297 | 0.014889676 | 0.00212298 |
| GOBAR_AA07825 | 34.926 | 31.696 | -0.511767254 | 0.00458718 | 0.000504639 |
| GOBAR_AA32260 | 28.033 | 22.983 | -0.514585776 | 0.022663573 | 0.003565917 |
| GOBAR_AA14245 | 32.036 | 26.366 | -0.51552388 | 0.049314105 | 0.009482769 |
| GOBAR_AA33110 | 64.606 | 52.876 | -0.520592818 | 0.024946064 | 0.004005753 |
| GOBAR_AA24311 | 9.816 | 7.95 | -0.522629506 | 0.041159467 | 0.007521153 |
| GOBAR_AA40284 | 90.66 | 73.21 | -0.528755461 | 0.006586028 | 0.000775431 |
| GOBAR_AA23795 | 33.263 | 26.373 | -0.528814268 | 0.044898066 | 0.008397556 |
| GOBAR_AA39045 | 7.59 | 6.123 | -0.531530749 | 0.04221143 | 0.007773123 |
| GOBAR_AA07123 | 13.446 | 10.9 | -0.531841675 | 0.008902721 | 0.001124113 |
| GOBAR_AA06077 | 182.96 | 143.743 | -0.552923636 | 0.008958325 | 0.001134775 |
| GOBAR_AA08422 | 146.026 | 131.653 | -0.556407657 | 0.00069556 | 5.63E-05 |
| GOBAR_AA11738 | 23.763 | 18.983 | -0.560245851 | 0.000367133 | 2.71E-05 |
| GOBAR_AA07933 | 40.383 | 31.883 | -0.564467447 | 0.008162147 | 0.001010513 |
| GOBAR_AA21100 | 25.346 | 20.066 | -0.566413366 | 0.009256575 | 0.001182373 |
| GOBAR_AA08958 | 15.44 | 12.143 | -0.57186832 | 0.021312547 | 0.003301632 |
| GOBAR_AA29448 | 25.996 | 20.203 | -0.58002963 | 0.039365225 | 0.007114609 |
| GOBAR_AA32162 | 11.886 | 9.28 | -0.581485511 | 0.007767379 | 0.000948626 |
| GOBAR_AA00450 | 72.78 | 56.383 | -0.584025511 | 0.016395737 | 0.002382234 |
| GOBAR_AA20529 | 20.303 | 16.24 | -0.584781337 | 0.017644741 | 0.002608561 |
| GOBAR_AA07866 | 101.686 | 78.443 | -0.594220053 | 0.007639141 | 0.000929434 |
| GOBAR_AA19884 | 8.393 | 6.463 | -0.598366131 | 0.022369879 | 0.003508076 |
| GOBAR_AA17635 | 15.323 | 11.783 | -0.608504984 | 0.002046731 | 0.000196638 |
| GOBAR_AA08993 | 3.09 | 2.346 | -0.611115271 | 0.037364396 | 0.006669014 |
| GOBAR_AA39010 | 33.66 | 25.35 | -0.614349765 | 0.01738247 | 0.002562256 |
| GOBAR_AA28238 | 6.503 | 5.333 | -0.619347387 | 0.025095869 | 0.00403645 |
| GOBAR_AA28774 | 7.706 | 5.543 | -0.622413421 | 0.046886843 | 0.008871106 |
| GOBAR_AA19525 | 29.26 | 22.003 | -0.632927654 | 0.036942303 | 0.006561663 |
| GOBAR_AA19778 | 9.556 | 7.283 | -0.63475442 | 0.021047554 | 0.003250853 |
| GOBAR_AA36768 | 13.206 | 9.87 | -0.635519312 | 0.008023448 | 0.000990097 |
| GOBAR_AA39375 | 13.343 | 9.87 | -0.636912156 | 0.026637709 | 0.004362801 |
| GOBAR_AA34342 | 68.103 | 52.96 | -0.637046525 | 0.02560524 | 0.004144885 |
| GOBAR_AA29232 | 39.776 | 29.316 | -0.656080966 | 0.001680418 | 0.000157319 |
| GOBAR_AA30228 | 19.333 | 14.27 | -0.657438571 | 0.004758274 | 0.000527176 |
| GOBAR_AA00034 | 13.046 | 9.563 | -0.658125667 | 0.000137935 | 8.69E-06 |
| GOBAR_AA33386 | 23.13 | 17.49 | -0.667442327 | 3.61E-05 | 1.94E-06 |
| GOBAR_AA35944 | 7.9 | 5.79 | -0.674839312 | 0.009149478 | 0.001166579 |
| GOBAR_AA25580 | 7.666 | 5.126 | -0.678804252 | 0.014334168 | 0.002029593 |
| GOBAR_AA34168 | 14.813 | 10.613 | -0.694587656 | 0.002781482 | 0.000280511 |
| GOBAR_AA10859 | 14.233 | 10.483 | -0.698325269 | 0.00248168 | 0.000245738 |
| GOBAR_AA09918 | 19.966 | 14.793 | -0.699045222 | 0.032495128 | 0.005594355 |
| GOBAR_AA35094 | 43.87 | 32.41 | -0.706418511 | 0.015475231 | 0.00222569 |
| GOBAR_AA31115 | 45.05 | 31.936 | -0.70826292 | 0.004882853 | 0.00054373 |
| GOBAR_AA19089 | 158.623 | 112.46 | -0.717864166 | 3.80E-05 | 2.05E-06 |
| GOBAR_AA30868 | 29.383 | 20.456 | -0.719568583 | 0.001026343 | 8.86E-05 |
| GOBAR_AA20826 | 11.146 | 7.816 | -0.726842268 | 0.002216785 | 0.000215346 |
| GOBAR_AA24869 | 2.846 | 2.073 | -0.730466793 | 0.012285978 | 0.001669053 |
| GOBAR_AA12983 | 7.33 | 5.23 | -0.736598221 | 0.012192941 | 0.001652892 |
| GOBAR_AA34792 | 6.843 | 4.676 | -0.742104946 | 0.0073933 | 0.000893598 |
| GOBAR_AA32390 | 844.586 | 581.856 | -0.743969132 | 0.006857251 | 0.000816278 |
| GOBAR_AA03672 | 30.813 | 21.16 | -0.766049878 | 0.00084874 | 7.12E-05 |
| GOBAR_AA27327 | 16.49 | 11.27 | -0.774517421 | 0.001168647 | 0.000103097 |
| GOBAR_AA03797 | 108.936 | 72.57 | -0.776045298 | 0.004734014 | 0.000523327 |
| GOBAR_AA31731 | 10.35 | 6.98 | -0.785770575 | 0.004293867 | 0.000467229 |
| GOBAR_AA11548 | 35.95 | 24.3 | -0.789625912 | 0.000395346 | 2.95E-05 |
| GOBAR_AA20279 | 8.34 | 5.56 | -0.801349708 | 0.012927357 | 0.001778216 |
| GOBAR_AA39282 | 12.846 | 7.523 | -0.804107465 | 0.027154258 | 0.004473781 |
| GOBAR_AA05688 | 4.093 | 2.433 | -0.815196955 | 0.039691151 | 0.007193226 |
| GOBAR_AA31796 | 5.7 | 3.78 | -0.816392552 | 0.003762329 | 0.000399718 |
| GOBAR_AA10179 | 5.42 | 3.666 | -0.822692693 | 0.017163676 | 0.002521594 |
| GOBAR_AA40383 | 5.56 | 3.61 | -0.823565925 | 0.012760337 | 0.001749531 |
| GOBAR_AA11010 | 15.216 | 9.92 | -0.844859226 | 0.002492723 | 0.000247191 |
| GOBAR_AA26433 | 68.726 | 43.693 | -0.846428715 | 6.97E-07 | 2.57E-08 |
| GOBAR_AA23338 | 155.063 | 100.746 | -0.846666508 | 0.000735865 | 6.00E-05 |
| GOBAR_AA16811 | 41.436 | 27.093 | -0.850940052 | 0.000986455 | 8.47E-05 |
| GOBAR_AA03366 | 27.846 | 17.546 | -0.860052151 | 0.006530706 | 0.000766654 |
| GOBAR_AA10643 | 3.11 | 1.986 | -0.860128684 | 0.009791776 | 0.001267141 |
| GOBAR_AA39102 | 10.403 | 6.456 | -0.869138261 | 0.024320317 | 0.003891011 |
| GOBAR_AA29465 | 7.256 | 4.583 | -0.874781844 | 0.006810706 | 0.000808524 |
| GOBAR_AA38083 | 10.276 | 6.52 | -0.8749366 | 0.016355048 | 0.00237585 |
| GOBAR_AA22501 | 6.166 | 3.683 | -0.874961666 | 0.018448799 | 0.002762071 |
| GOBAR_AA02514 | 13.686 | 8.65 | -0.875486602 | 0.000522955 | 4.05E-05 |
| GOBAR_AA16342 | 12.1 | 7.63 | -0.886669054 | 0.000396825 | 2.96E-05 |
| GOBAR_AA39076 | 9.576 | 6.016 | -0.89005946 | 0.023727326 | 0.003779895 |
| GOBAR_AA22497 | 195.786 | 123.05 | -0.89206064 | 6.39E-06 | 2.87E-07 |
| GOBAR_AA20655 | 310.123 | 191.606 | -0.904967462 | 1.16E-06 | 4.49E-08 |
| GOBAR_AA00737 | 15.07 | 9.416 | -0.905207581 | 0.000218772 | 1.49E-05 |
| GOBAR_AA21096 | 8.92 | 5.06 | -0.909248611 | 0.00085309 | 7.17E-05 |
| GOBAR_AA27426 | 7.85 | 4.776 | -0.909788198 | 1.81E-05 | 9.02E-07 |
| GOBAR_AA01433 | 22.086 | 13.556 | -0.912736655 | 3.61E-05 | 1.94E-06 |
| GOBAR_AA13750 | 9.473 | 5.78 | -0.919536103 | 0.002541684 | 0.000252634 |
| GOBAR_AA14420 | 2.386 | 1.44 | -0.928755372 | 0.029979818 | 0.005056534 |
| GOBAR_AA35089 | 275 | 170.3 | -0.937260709 | 0.009005217 | 0.001142293 |
| GOBAR_AA15741 | 68.893 | 41.14 | -0.943068653 | 1.30E-05 | 6.25E-07 |
| GOBAR_AA36706 | 5.38 | 3.14 | -0.949061015 | 0.013918768 | 0.001950372 |
| GOBAR_AA30334 | 47.76 | 29.316 | -0.971669057 | 0.002221645 | 0.000216074 |
| GOBAR_AA06526 | 71.386 | 41.566 | -0.981433534 | 3.97E-05 | 2.15E-06 |
| GOBAR_AA39004 | 42.806 | 25.21 | -0.984436476 | 1.44E-07 | 4.74E-09 |
| GOBAR_AA34478 | 16.323 | 9.256 | -0.986219653 | 0.024417127 | 0.003908828 |
| GOBAR_AA03757 | 3.763 | 2.156 | -0.997874834 | 0.028072178 | 0.004662614 |
| GOBAR_AA00467 | 11.643 | 6.58 | -1.002536216 | 0.035544791 | 0.006251834 |
| GOBAR_AA36268 | 37.216 | 21.56 | -1.019699448 | 6.52E-05 | 3.75E-06 |
| GOBAR_AA35339 | 6.66 | 3.65 | -1.02190166 | 0.000145076 | 9.22E-06 |
| GOBAR_AA40147 | 7.973 | 4.556 | -1.030145196 | 1.79E-05 | 8.95E-07 |
| GOBAR_AA30061 | 5.953 | 3.273 | -1.037028029 | 0.023688559 | 0.003769614 |
| GOBAR_AA16118 | 6.92 | 4.213 | -1.050470555 | 0.000134501 | 8.44E-06 |
| GOBAR_AA36233 | 30.576 | 16.836 | -1.052975907 | 0.000192949 | 1.28E-05 |
| GOBAR_AA33921 | 7.933 | 4.41 | -1.059161629 | 4.53E-05 | 2.51E-06 |
| GOBAR_AA33567 | 13.723 | 7.443 | -1.112171199 | 7.84E-08 | 2.47E-09 |
| GOBAR_AA00282 | 8.58 | 4.47 | -1.118705339 | 0.00507623 | 0.000570249 |
| GOBAR_AA07448 | 26.47 | 13.636 | -1.157217891 | 3.04E-10 | 6.95E-12 |
| GOBAR_AA26291 | 11.916 | 6.02 | -1.162086901 | 2.24E-05 | 1.13E-06 |
| GOBAR_AA35052 | 2.98 | 1.496 | -1.163853718 | 0.026889215 | 0.004414867 |
| GOBAR_AA39841 | 12.033 | 5.666 | -1.170180627 | 0.00160078 | 0.000148558 |
| GOBAR_AA09331 | 78.063 | 41.366 | -1.173925029 | 0.000382265 | 2.84E-05 |
| GOBAR_AA24402 | 19.586 | 9.946 | -1.188037841 | 1.90E-12 | 3.40E-14 |
| GOBAR_AA28714 | 10.753 | 5.793 | -1.195553119 | 0.013798438 | 0.001929127 |
| GOBAR_AA25667 | 7.7 | 4.723 | -1.203208353 | 0.000842938 | 7.06E-05 |
| GOBAR_AA35715 | 42.806 | 20.743 | -1.207034216 | 0.026535151 | 0.004338339 |
| GOBAR_AA13316 | 28.27 | 15.846 | -1.217295753 | 3.59E-11 | 7.48E-13 |
| GOBAR_AA03662 | 3.36 | 1.98 | -1.218030939 | 0.000848404 | 7.11E-05 |
| GOBAR_AA29494 | 34.83 | 17.38 | -1.225480309 | 1.08E-06 | 4.15E-08 |
| GOBAR_AA33208 | 9.11 | 4.496 | -1.226950775 | 5.12E-09 | 1.38E-10 |
| GOBAR_AA38917 | 9.033 | 4.336 | -1.22797756 | 1.67E-06 | 6.68E-08 |
| GOBAR_AA20995 | 37.926 | 18.19 | -1.233239435 | 0.013156513 | 0.001817719 |
| GOBAR_AA22000 | 11.443 | 5.53 | -1.238398172 | 5.32E-05 | 2.98E-06 |
| GOBAR_AA30170 | 89.93 | 42.126 | -1.249787888 | 4.41E-10 | 1.04E-11 |
| GOBAR_AA05834 | 8.446 | 3.966 | -1.281284465 | 0.00119629 | 0.000105845 |
| GOBAR_AA20308 | 10.39 | 4.903 | -1.291254253 | 0.001365022 | 0.000122942 |
| GOBAR_AA24959 | 24.096 | 10.136 | -1.298821647 | 0.000214083 | 1.44E-05 |
| GOBAR_AA23779 | 8.2 | 3.756 | -1.307500384 | 0.00021437 | 1.45E-05 |
| GOBAR_AA14759 | 4.93 | 2.256 | -1.332404442 | 0.000682453 | 5.50E-05 |
| GOBAR_AA16776 | 73.593 | 32.236 | -1.345932365 | 0.000940584 | 8.01E-05 |
| GOBAR_AA22143 | 75.206 | 34.126 | -1.350020915 | 0.000111514 | 6.85E-06 |
| GOBAR_AA35762 | 3.56 | 1.483 | -1.355362854 | 0.03514157 | 0.006155535 |
| GOBAR_AA22216 | 1.853 | 0.786 | -1.358652307 | 0.027277202 | 0.004497969 |
| GOBAR_AA39092 | 4.413 | 1.96 | -1.361274388 | 9.99E-06 | 4.71E-07 |
| GOBAR_AA31898 | 114.24 | 51.573 | -1.362270363 | 5.56E-18 | 6.28E-20 |
| GOBAR_AA06281 | 5.653 | 2.456 | -1.371368019 | 0.009763574 | 0.001262081 |
| GOBAR_AA22247 | 1.446 | 0.616 | -1.379960769 | 0.048922638 | 0.009386295 |
| GOBAR_AA08934 | 1.433 | 0.59 | -1.383924915 | 0.043923387 | 0.008171933 |
| GOBAR_AA13919 | 3.233 | 1.3 | -1.401869258 | 0.026269004 | 0.004280408 |
| GOBAR_AA17952 | 47.926 | 20.693 | -1.404463692 | 1.54E-05 | 7.56E-07 |
| GOBAR_AA31016 | 188.64 | 75.71 | -1.406442896 | 0.018256578 | 0.002726437 |
| GOBAR_AA03547 | 37.016 | 16.14 | -1.409226072 | 9.24E-14 | 1.45E-15 |
| GOBAR_AA12921 | 5.76 | 2.46 | -1.414533037 | 5.91E-05 | 3.36E-06 |
| GOBAR_AA39845 | 4.943 | 2.013 | -1.415757859 | 0.041953174 | 0.007703145 |
| GOBAR_AA29440 | 61.793 | 26.83 | -1.421502695 | 9.85E-17 | 1.21E-18 |
| GOBAR_AA28493 | 5.346 | 2.22 | -1.447754156 | 2.65E-05 | 1.37E-06 |
| GOBAR_AA19185 | 108.116 | 46.673 | -1.451789776 | 3.01E-10 | 6.88E-12 |
| GOBAR_AA15682 | 25.83 | 10.773 | -1.452820291 | 0.00036795 | 2.71E-05 |
| GOBAR_AA01555 | 30.7 | 12.48 | -1.465254946 | 9.18E-13 | 1.61E-14 |
| GOBAR_AA24401 | 14.953 | 6.183 | -1.478584553 | 1.69E-05 | 8.38E-07 |
| GOBAR_AA34732 | 33.81 | 15.066 | -1.547217874 | 1.99E-09 | 5.12E-11 |
| GOBAR_AA36626 | 20.526 | 8.016 | -1.547930839 | 2.17E-10 | 4.89E-12 |
| GOBAR_AA26636 | 65.003 | 25.736 | -1.562144295 | 1.00E-14 | 1.45E-16 |
| GOBAR_AA30639 | 1.106 | 0.366 | -1.5935153 | 0.034462148 | 0.00600467 |
| GOBAR_AA22689 | 5.33 | 2.136 | -1.59890927 | 0.000254318 | 1.77E-05 |
| GOBAR_AA04696 | 1.286 | 0.44 | -1.607034908 | 0.019043529 | 0.002873665 |
| GOBAR_AA24413 | 9.45 | 3.65 | -1.62523452 | 2.79E-13 | 4.61E-15 |
| GOBAR_AA37796 | 165.433 | 58.566 | -1.645319591 | 3.85E-13 | 6.53E-15 |
| GOBAR_AA14393 | 21.353 | 7.643 | -1.683009824 | 7.09E-13 | 1.22E-14 |
| GOBAR_AA33377 | 4.383 | 1.703 | -1.701547552 | 8.25E-09 | 2.28E-10 |
| GOBAR_AA12784 | 260.616 | 87.536 | -1.770224712 | 2.86E-11 | 5.83E-13 |
| GOBAR_AA19970 | 39.163 | 12.636 | -1.836256783 | 7.88E-26 | 5.26E-28 |
| GOBAR_AA24230 | 2.806 | 0.813 | -1.846538632 | 0.001669391 | 0.000155949 |
| GOBAR_AA31881 | 7.253 | 1.873 | -1.895899329 | 0.011049287 | 0.001464025 |
| GOBAR_AA12553 | 0.326 | 0.086 | -1.90587421 | 0.010744302 | 0.001414925 |
| GOBAR_AA06036 | 6.943 | 1.953 | -1.942371847 | 0.000409778 | 3.07E-05 |
| GOBAR_AA03171 | 428.13 | 126.35 | -1.95233553 | 2.96E-25 | 2.05E-27 |
| GOBAR_AA30597 | 9.59 | 2.576 | -1.986265214 | 1.57E-06 | 6.25E-08 |
| GOBAR_AA03589 | 20.466 | 5.576 | -1.99763991 | 1.48E-24 | 1.08E-26 |
| GOBAR_AA29872 | 2.256 | 0.593 | -2.009776774 | 0.000582093 | 4.58E-05 |
| GOBAR_AA31390 | 10.883 | 3.156 | -2.026402878 | 3.82E-06 | 1.65E-07 |
| GOBAR_AA26978 | 24.976 | 6.926 | -2.029721067 | 5.07E-14 | 7.81E-16 |
| GOBAR_AA08880 | 2 | 0.516 | -2.03362808 | 0.000195799 | 1.30E-05 |
| GOBAR_AA21203 | 0.67 | 0.166 | -2.042932439 | 0.007769153 | 0.000949067 |
| GOBAR_AA26005 | 5.903 | 1.49 | -2.134198678 | 0.000240724 | 1.66E-05 |
| GOBAR_AA05372 | 2.083 | 0.343 | -2.155110203 | 0.028421093 | 0.004734717 |
| GOBAR_AA35193 | 2.27 | 0.736 | -2.175277735 | 3.18E-06 | 1.36E-07 |
| GOBAR_AA25446 | 2.313 | 0.536 | -2.210917767 | 8.98E-06 | 4.17E-07 |
| GOBAR_AA07152 | 23.886 | 5.396 | -2.226733642 | 2.53E-08 | 7.39E-10 |
| GOBAR_AA40450 | 1.563 | 0.363 | -2.247956838 | 4.62E-07 | 1.66E-08 |
| GOBAR_AA33408 | 5.803 | 1.346 | -2.286658913 | 2.15E-10 | 4.83E-12 |
| GOBAR_AA08879 | 3.146 | 0.586 | -2.31169981 | 0.006239681 | 0.000728164 |
| GOBAR_AA29350 | 4.696 | 0.9 | -2.316367085 | 0.000612541 | 4.86E-05 |
| GOBAR_AA31136 | 2.036 | 0.433 | -2.355283214 | 9.56E-07 | 3.65E-08 |
| GOBAR_AA04207 | 1.076 | 0.086 | -2.372675286 | 0.037871133 | 0.006786808 |
| GOBAR_AA14053 | 0.356 | 0 | -2.385757225 | 0.0482719 | 0.009211247 |
| GOBAR_AA08892 | 1.356 | 0.163 | -2.405465353 | 0.018197406 | 0.002714446 |
| GOBAR_AA09730 | 9.063 | 1.643 | -2.454132743 | 9.22E-07 | 3.52E-08 |
| GOBAR_AA26633 | 16.116 | 2.056 | -2.467174042 | 0.008850582 | 0.001116197 |
| GOBAR_AA36042 | 26.68 | 6.103 | -2.491839581 | 4.71E-45 | 1.59E-47 |
| GOBAR_AA22524 | 12.253 | 2.353 | -2.492895947 | 3.08E-08 | 9.11E-10 |
| GOBAR_AA01907 | 5.053 | 0.983 | -2.512281924 | 4.61E-16 | 5.96E-18 |
| GOBAR_AA36705 | 10.85 | 2.293 | -2.51690966 | 9.83E-31 | 5.42E-33 |
| GOBAR_AA14381 | 8.69 | 1.88 | -2.532437219 | 1.21E-28 | 7.40E-31 |
| GOBAR_AA17400 | 57.686 | 11.29 | -2.556116566 | 8.06E-13 | 1.40E-14 |
| GOBAR_AA28251 | 0.82 | 0.08 | -2.576392327 | 0.011189142 | 0.001487081 |
| GOBAR_AA14791 | 12.52 | 2.206 | -2.595672076 | 4.37E-08 | 1.32E-09 |
| GOBAR_AA34163 | 2.333 | 0.666 | -2.606373387 | 2.80E-11 | 5.69E-13 |
| GOBAR_AA15055 | 1.43 | 0 | -2.732167209 | 0.018041117 | 0.002685922 |
| GOBAR_AA04233 | 79.12 | 12.596 | -2.747918959 | 6.15E-31 | 3.34E-33 |
| GOBAR_AA12958 | 17.086 | 2.536 | -2.849271425 | 4.97E-09 | 1.33E-10 |
| GOBAR_AA25679 | 6.23 | 0.96 | -2.853852984 | 3.57E-12 | 6.66E-14 |
| GOBAR_AA18783 | 18.573 | 1.313 | -2.887302098 | 0.005328865 | 0.000606462 |
| GOBAR_AA10353 | 1.9 | 0.236 | -2.922354777 | 1.38E-06 | 5.45E-08 |
| GOBAR_AA09251 | 0.703 | 0 | -2.941632633 | 0.009397202 | 0.001203593 |
| GOBAR_AA39007 | 58.04 | 8.403 | -2.955829201 | 5.89E-38 | 2.55E-40 |
| GOBAR_AA04563 | 22.696 | 3.053 | -2.991153103 | 3.22E-13 | 5.36E-15 |
| GOBAR_AA29014 | 2.263 | 0.326 | -3.009182677 | 3.76E-15 | 5.28E-17 |
| GOBAR_AA07777 | 249.636 | 36.46 | -3.047375724 | 3.50E-114 | 2.53E-117 |
| GOBAR_AA07044 | 1.976 | 0 | -3.108644372 | 0.004809441 | 0.000533743 |
| GOBAR_AA11600 | 47.743 | 6.11 | -3.13125157 | 1.94E-20 | 1.81E-22 |
| GOBAR_AA29066 | 98.38 | 14.743 | -3.160335841 | 7.73E-28 | 4.87E-30 |
| GOBAR_AA35470 | 99.596 | 5.28 | -3.228510191 | 0.000734148 | 5.99E-05 |
| GOBAR_AA30819 | 4.643 | 0.543 | -3.250996566 | 1.32E-28 | 8.14E-31 |
| GOBAR_AA02317 | 1.77 | 0.163 | -3.259344057 | 2.90E-05 | 1.51E-06 |
| GOBAR_AA01941 | 10.256 | 0 | -3.474129475 | 0.0015021 | 0.000137892 |
| GOBAR_AA12643 | 7.993 | 0.816 | -3.504711051 | 4.73E-35 | 2.30E-37 |
| GOBAR_AA12520 | 0.636 | 0.016 | -3.576906544 | 0.000184267 | 1.22E-05 |
| GOBAR_AA33670 | 2.49 | 0.103 | -3.833552302 | 1.81E-06 | 7.34E-08 |
| GOBAR_AA36252 | 0.84 | 0 | -3.834596427 | 0.000164316 | 1.06E-05 |
| GOBAR_AA03659 | 102.493 | 7.806 | -3.852839944 | 1.28E-89 | 1.48E-92 |
| GOBAR_AA35700 | 27.943 | 1.57 | -3.917732189 | 1.77E-10 | 3.94E-12 |
| GOBAR_AA01086 | 1.46 | 0.02 | -3.923986371 | 4.28E-05 | 2.35E-06 |
| GOBAR_AA38817 | 267.06 | 0.41 | -3.940651139 | 0.000190466 | 1.26E-05 |
| GOBAR_AA10203 | 6.213 | 0.416 | -4.038691792 | 6.21E-24 | 4.68E-26 |
| GOBAR_AA01768 | 37.523 | 2.54 | -4.046865323 | 8.68E-37 | 4.01E-39 |
| GOBAR_AA09859 | 10.336 | 0.64 | -4.072949248 | 2.42E-25 | 1.66E-27 |
| GOBAR_AA03206 | 19.33 | 1.096 | -4.180778716 | 1.36E-23 | 1.05E-25 |
| GOBAR_AA32600 | 239.203 | 10.923 | -4.442660169 | 1.07E-74 | 1.61E-77 |
| GOBAR_AA21005 | 1.673 | 0.053 | -4.449000922 | 4.79E-11 | 1.01E-12 |
| GOBAR_AA33731 | 3.143 | 0.19 | -4.66445159 | 1.82E-24 | 1.33E-26 |
| GOBAR_AA17615 | 655.546 | 24.42 | -4.872433545 | 2.03E-75 | 2.94E-78 |
| GOBAR_AA00612 | 48.503 | 1.593 | -5.00501939 | 1.42E-50 | 4.08E-53 |
| GOBAR_AA28360 | 5675.503 | 6.256 | -5.561438842 | 3.15E-09 | 8.23E-11 |
| GOBAR_AA31329 | 2.07 | 0 | -6.222623074 | 7.43E-15 | 1.06E-16 |
| GOBAR_AA28220 | 200.55 | 1.433 | -6.785246077 | 4.17E-41 | 1.61E-43 |
| GOBAR_AA11009 | 10.423 | 0.013 | -7.440898491 | 3.36E-29 | 1.99E-31 |
