# Supplementary material for: Integrated Methylome and Transcriptome Analysis Widen the Knowledge of Cytoplasmic Male Sterility in Cotton (Gossypium barbadense L.)
Source: Front Plant Sci. 2022 Apr 27;13:770098. doi: 10.3389/fpls.2022.770098 (PMC9093596; doi:10.3389/fpls.2022.770098)
Supplement: Supplementary file 10 [file Table_10.DOCX]

**Table S10** Up-regulated and down-regulated genes in the hypo-DEGs（ZA:07-113A,ZB:07-113B）

| Gene ID | ZB FPKM | ZA FPKM | log2(ZA/ZB) | Qvalue(ZB-vs-ZA) | Pvalue(ZB-vs-ZA) |
| --- | --- | --- | --- | --- | --- |
| GOBAR_AA08371 | 0 | 4.14 | 4.805450237 | 1.09E-07 | 3.50E-09 |
| GOBAR_AA25984 | 0 | 2.063 | 4.460317876 | 2.40E-06 | 1.00E-07 |
| GOBAR_AA00180 | 0.01 | 1.25 | 4.38381639 | 8.93E-07 | 3.39E-08 |
| GOBAR_AA22117 | 27.553 | 600.196 | 4.175368442 | 2.21E-69 | 3.82E-72 |
| GOBAR_AA09464 | 0 | 2.736 | 3.73375814 | 0.000317967 | 2.29E-05 |
| GOBAR_AA27494 | 0 | 3.573 | 3.679854211 | 0.000305884 | 2.19E-05 |
| GOBAR_AA36533 | 0.02 | 0.603 | 3.373825931 | 0.000263622 | 1.85E-05 |
| GOBAR_AA20233 | 0 | 0.286 | 3.339540242 | 0.001722284 | 0.000161857 |
| GOBAR_AA28336 | 0.263 | 4.613 | 3.287628685 | 8.93E-06 | 4.14E-07 |
| GOBAR_AA25907 | 0.05 | 0.893 | 3.215116666 | 0.000112808 | 6.94E-06 |
| GOBAR_AA20954 | 0.19 | 2.676 | 3.194249745 | 1.99E-06 | 8.11E-08 |
| GOBAR_AA09839 | 1.126 | 12.093 | 3.00208738 | 3.19E-10 | 7.32E-12 |
| GOBAR_AA10017 | 0.083 | 1.076 | 2.98190794 | 9.72E-05 | 5.86E-06 |
| GOBAR_AA40226 | 0.1 | 1.94 | 2.919925274 | 0.002687072 | 0.000269413 |
| GOBAR_AA07576 | 0.21 | 2.256 | 2.820328241 | 0.000122716 | 7.61E-06 |
| GOBAR_AA27160 | 0.606 | 5.883 | 2.72252255 | 1.25E-05 | 6.03E-07 |
| GOBAR_AA27788 | 8.06 | 111.336 | 2.660060659 | 0.007982065 | 0.000983693 |
| GOBAR_AA00012 | 0 | 0.253 | 2.64767932 | 0.023340005 | 0.003700664 |
| GOBAR_AA01007 | 0.726 | 6 | 2.620608179 | 0.000800047 | 6.64E-05 |
| GOBAR_AA27076 | 1.776 | 14.203 | 2.599386671 | 3.91E-05 | 2.12E-06 |
| GOBAR_AA29483 | 0.78 | 5.533 | 2.432850691 | 0.000408426 | 3.06E-05 |
| GOBAR_AA02965 | 0.893 | 6.3 | 2.404181509 | 2.42E-06 | 1.01E-07 |
| GOBAR_AA01568 | 1.096 | 6.656 | 2.285463016 | 3.14E-07 | 1.09E-08 |
| GOBAR_AA08909 | 0.103 | 0.746 | 2.244248955 | 0.003433729 | 0.000358657 |
| GOBAR_AA36954 | 2.09 | 10.793 | 2.20620869 | 1.01E-05 | 4.75E-07 |
| GOBAR_AA06692 | 0.06 | 0.463 | 2.202333991 | 0.016264756 | 0.002359444 |
| GOBAR_AA37329 | 24.76 | 137.346 | 2.144200082 | 8.50E-11 | 1.84E-12 |
| GOBAR_AA40391 | 0.093 | 0.573 | 2.111490706 | 0.003644204 | 0.000384432 |
| GOBAR_AA27999 | 0.536 | 2.893 | 2.08041683 | 0.00050251 | 3.88E-05 |
| GOBAR_AA29776 | 1.206 | 8.286 | 2.0670993 | 8.01E-08 | 2.52E-09 |
| GOBAR_AA39182 | 0.266 | 1.536 | 1.978291533 | 0.018391859 | 0.002751952 |
| GOBAR_AA40302 | 0.136 | 0.826 | 1.961259179 | 0.026330961 | 0.004292025 |
| GOBAR_AA10688 | 18.263 | 86.396 | 1.953963038 | 8.56E-09 | 2.38E-10 |
| GOBAR_AA37399 | 0.09 | 0.516 | 1.948217093 | 0.022823079 | 0.003596288 |
| GOBAR_AA17752 | 0.113 | 0.64 | 1.926903663 | 0.027064001 | 0.004453728 |
| GOBAR_AA16012 | 1.513 | 7.473 | 1.893800119 | 0.010506433 | 0.001377226 |
| GOBAR_AA28454 | 0.13 | 0.766 | 1.879653004 | 0.044972819 | 0.008414135 |
| GOBAR_AA29037 | 7.843 | 36.603 | 1.866873118 | 4.81E-05 | 2.68E-06 |
| GOBAR_AA33977 | 5.43 | 24.33 | 1.793059593 | 0.006166262 | 0.000718706 |
| GOBAR_AA38726 | 1.206 | 5.296 | 1.781444194 | 0.006511092 | 0.000763975 |
| GOBAR_AA24735 | 1.56 | 6.68 | 1.753375375 | 0.000251038 | 1.74E-05 |
| GOBAR_AA35935 | 0.13 | 0.613 | 1.747044196 | 0.049675211 | 0.009569427 |
| GOBAR_AA22364 | 2.026 | 8.716 | 1.71335257 | 0.010597467 | 0.00139069 |
| GOBAR_AA20558 | 4.743 | 18.56 | 1.712868344 | 2.88E-10 | 6.55E-12 |
| GOBAR_AA14676 | 13.153 | 51.003 | 1.703081208 | 2.89E-07 | 9.97E-09 |
| GOBAR_AA33790 | 0.08 | 0.326 | 1.690830593 | 0.033152917 | 0.00574399 |
| GOBAR_AA23951 | 0.533 | 2.206 | 1.68702561 | 0.00255994 | 0.00025504 |
| GOBAR_AA18614 | 0.606 | 2.386 | 1.678408447 | 0.000563625 | 4.42E-05 |
| GOBAR_AA21232 | 2.146 | 8.646 | 1.67687231 | 0.000839863 | 7.02E-05 |
| GOBAR_AA08006 | 1.893 | 7.446 | 1.674148528 | 0.000453384 | 3.44E-05 |
| GOBAR_AA06049 | 39.163 | 147.856 | 1.673275923 | 1.71E-22 | 1.41E-24 |
| GOBAR_AA23346 | 0.466 | 1.86 | 1.646613051 | 0.006686887 | 0.000790459 |
| GOBAR_AA37753 | 0.73 | 2.916 | 1.627465301 | 0.013473404 | 0.001874344 |
| GOBAR_AA32263 | 1.026 | 4.146 | 1.601970584 | 0.046078016 | 0.008676506 |
| GOBAR_AA11537 | 1.49 | 5.456 | 1.594841783 | 0.001532735 | 0.000141191 |
| GOBAR_AA21224 | 2.506 | 8.803 | 1.542072285 | 1.16E-07 | 3.78E-09 |
| GOBAR_AA01307 | 0.436 | 1.576 | 1.511961612 | 0.001306638 | 0.000117042 |
| GOBAR_AA37171 | 12.786 | 43.686 | 1.496556505 | 1.45E-05 | 7.10E-07 |
| GOBAR_AA14640 | 7.37 | 22.883 | 1.372670253 | 2.07E-10 | 4.65E-12 |
| GOBAR_AA35126 | 0.433 | 1.353 | 1.360127134 | 0.009898065 | 0.001283469 |
| GOBAR_AA19948 | 11.7 | 26.07 | 1.350797871 | 0.006020018 | 0.000697313 |
| GOBAR_AA35264 | 3.893 | 12.026 | 1.338397048 | 0.003728446 | 0.000395306 |
| GOBAR_AA01934 | 2.42 | 6.326 | 1.333813036 | 0.045761785 | 0.00860008 |
| GOBAR_AA00449 | 1.3 | 3.893 | 1.295190693 | 0.018234185 | 0.002720986 |
| GOBAR_AA40378 | 0.29 | 0.88 | 1.286416996 | 0.023321905 | 0.003696447 |
| GOBAR_AA39861 | 14.403 | 41.31 | 1.281596396 | 9.79E-05 | 5.91E-06 |
| GOBAR_AA34025 | 2.856 | 8.23 | 1.279572451 | 0.000912815 | 7.75E-05 |
| GOBAR_AA37962 | 4.243 | 12.373 | 1.276116624 | 1.44E-05 | 7.06E-07 |
| GOBAR_AA34516 | 10.266 | 30.253 | 1.258397794 | 0.002140959 | 0.00020699 |
| GOBAR_AA34246 | 2.51 | 7.196 | 1.252337828 | 0.000103022 | 6.27E-06 |
| GOBAR_AA22149 | 16.156 | 46.203 | 1.248345272 | 0.000519825 | 4.02E-05 |
| GOBAR_AA23527 | 7.833 | 22.053 | 1.229450429 | 0.017867516 | 0.002651818 |
| GOBAR_AA30306 | 6.29 | 17.19 | 1.208872877 | 1.21E-10 | 2.65E-12 |
| GOBAR_AA18336 | 6.36 | 17.38 | 1.189942396 | 5.90E-06 | 2.63E-07 |
| GOBAR_AA06047 | 1.976 | 5.426 | 1.189337105 | 0.000561233 | 4.40E-05 |
| GOBAR_AA30941 | 0.79 | 2.16 | 1.155485578 | 0.020226334 | 0.003100059 |
| GOBAR_AA23209 | 17.63 | 46.73 | 1.148532257 | 0.001038409 | 8.97E-05 |
| GOBAR_AA12762 | 2.7 | 7.173 | 1.14311987 | 0.000964676 | 8.26E-05 |
| GOBAR_AA12619 | 3.15 | 8.343 | 1.142140579 | 0.035872925 | 0.006324156 |
| GOBAR_AA13934 | 0.673 | 1.933 | 1.1230564 | 0.036053783 | 0.006360104 |
| GOBAR_AA34419 | 10.076 | 26.186 | 1.120594421 | 8.43E-05 | 5.00E-06 |
| GOBAR_AA06524 | 1.046 | 2.7 | 1.116503737 | 0.004035351 | 0.000434466 |
| GOBAR_AA06740 | 19.833 | 51.64 | 1.111814606 | 0.000117652 | 7.27E-06 |
| GOBAR_AA02709 | 5.833 | 14.666 | 1.096725302 | 0.000406455 | 3.04E-05 |
| GOBAR_AA39720 | 26.386 | 66.653 | 1.095597824 | 0.000615651 | 4.89E-05 |
| GOBAR_AA00229 | 46.81 | 122.376 | 1.090698295 | 0.000166605 | 1.08E-05 |
| GOBAR_AA20313 | 8.51 | 20.943 | 1.086742032 | 0.009378178 | 0.001200886 |
| GOBAR_AA23969 | 4.936 | 12.8 | 1.082137121 | 4.96E-05 | 2.77E-06 |
| GOBAR_AA16698 | 5.16 | 15.216 | 1.070714026 | 0.000126386 | 7.87E-06 |
| GOBAR_AA15108 | 22.43 | 55.2 | 1.064385652 | 0.00443404 | 0.000485427 |
| GOBAR_AA03562 | 5.7 | 14.376 | 1.061718662 | 0.041318672 | 0.007560986 |
| GOBAR_AA28753 | 75.483 | 182.333 | 1.056085339 | 1.43E-05 | 6.95E-07 |
| GOBAR_AA14051 | 30.726 | 73.406 | 1.051686238 | 0.00010357 | 6.31E-06 |
| GOBAR_AA34517 | 1.246 | 3.096 | 1.047735328 | 0.018861617 | 0.002834228 |
| GOBAR_AA02699 | 5.21 | 12.686 | 1.043365648 | 1.68E-05 | 8.30E-07 |
| GOBAR_AA11392 | 3.68 | 10.66 | 1.041546464 | 0.00200407 | 0.000191556 |
| GOBAR_AA16999 | 22.94 | 55.84 | 1.035744391 | 0.02419853 | 0.003867704 |
| GOBAR_AA12986 | 6.91 | 17.296 | 1.033405805 | 0.006899289 | 0.000823275 |
| GOBAR_AA26546 | 2.993 | 7.416 | 1.032526048 | 0.018919107 | 0.002845052 |
| GOBAR_AA25540 | 9.736 | 23.346 | 1.022804013 | 0.037613761 | 0.00672982 |
| GOBAR_AA10704 | 31.35 | 72.026 | 1.022128018 | 0.045610149 | 0.008564996 |
| GOBAR_AA11709 | 12.596 | 34.076 | 1.016116255 | 0.010044362 | 0.001306791 |
| GOBAR_AA08214 | 4.23 | 10.103 | 1.009268082 | 6.54E-05 | 3.77E-06 |
| GOBAR_AA21999 | 8.703 | 20.443 | 0.990551547 | 0.000215172 | 1.46E-05 |
| GOBAR_AA34154 | 9.973 | 23.963 | 0.985602916 | 0.003836803 | 0.000409404 |
| GOBAR_AA13859 | 39.06 | 90.856 | 0.98014425 | 1.52E-05 | 7.47E-07 |
| GOBAR_AA30755 | 3.416 | 8.156 | 0.97890934 | 0.013473404 | 0.001873994 |
| GOBAR_AA29570 | 24.653 | 55.376 | 0.972804552 | 0.036385606 | 0.006436507 |
| GOBAR_AA19518 | 3.153 | 7.253 | 0.968935599 | 0.011221558 | 0.001493982 |
| GOBAR_AA11168 | 15.85 | 36.406 | 0.96820498 | 0.008868039 | 0.001118655 |
| GOBAR_AA24272 | 18.286 | 41.733 | 0.952309871 | 1.02E-06 | 3.91E-08 |
| GOBAR_AA26364 | 25.06 | 45.8 | 0.950269589 | 0.005092897 | 0.000573298 |
| GOBAR_AA08368 | 3.976 | 9.043 | 0.940143653 | 0.000204272 | 1.37E-05 |
| GOBAR_AA02697 | 34.76 | 78.013 | 0.933767656 | 7.33E-06 | 3.33E-07 |
| GOBAR_AA17482 | 3.233 | 7.763 | 0.930120521 | 0.041553234 | 0.007609911 |
| GOBAR_AA09673 | 4.063 | 9.056 | 0.921886523 | 0.004415435 | 0.000483136 |
| GOBAR_AA31609 | 58.506 | 123.313 | 0.912692795 | 6.56E-06 | 2.95E-07 |
| GOBAR_AA13674 | 11.476 | 25.486 | 0.91174142 | 0.000259208 | 1.81E-05 |
| GOBAR_AA22472 | 1.536 | 3.506 | 0.907758218 | 0.017521715 | 0.0025843 |
| GOBAR_AA27157 | 4.42 | 9.7 | 0.906879292 | 0.005543279 | 0.000635526 |
| GOBAR_AA02267 | 7.33 | 16.013 | 0.903436724 | 0.000365662 | 2.69E-05 |
| GOBAR_AA03213 | 17.34 | 39.593 | 0.895088864 | 0.000690179 | 5.58E-05 |
| GOBAR_AA17188 | 5.546 | 12.033 | 0.882617892 | 0.001136611 | 9.99E-05 |
| GOBAR_AA30234 | 12.766 | 27.983 | 0.879731952 | 0.000156552 | 1.00E-05 |
| GOBAR_AA01088 | 11.793 | 25.753 | 0.873596424 | 0.006059332 | 0.000703617 |
| GOBAR_AA29256 | 43.743 | 94.693 | 0.872676834 | 1.98E-06 | 8.08E-08 |
| GOBAR_AA08434 | 9.963 | 21.373 | 0.869331371 | 0.002197753 | 0.000213179 |
| GOBAR_AA33351 | 29.74 | 64.98 | 0.859141553 | 0.001061738 | 9.23E-05 |
| GOBAR_AA00048 | 6.103 | 13.15 | 0.85831318 | 0.006072855 | 0.000705714 |
| GOBAR_AA35519 | 12.59 | 26.746 | 0.857756344 | 0.00021499 | 1.45E-05 |
| GOBAR_AA20036 | 10.606 | 23.04 | 0.853792298 | 0.022276635 | 0.003491523 |
| GOBAR_AA12693 | 2.69 | 5.773 | 0.849451615 | 0.026427954 | 0.004313942 |
| GOBAR_AA01872 | 5.376 | 11.723 | 0.843477911 | 0.003693704 | 0.000390451 |
| GOBAR_AA24855 | 29.933 | 64.33 | 0.833891826 | 0.000592796 | 4.67E-05 |
| GOBAR_AA38653 | 25.83 | 54.35 | 0.831680413 | 0.007205693 | 0.000866049 |
| GOBAR_AA15330 | 6.23 | 12.25 | 0.823342034 | 0.021928513 | 0.003417629 |
| GOBAR_AA27069 | 12.323 | 26.003 | 0.82187629 | 0.010843624 | 0.001432826 |
| GOBAR_AA03237 | 3.07 | 6.66 | 0.81375357 | 0.034184104 | 0.005947337 |
| GOBAR_AA11275 | 32.663 | 67.783 | 0.811487537 | 0.008827009 | 0.001112715 |
| GOBAR_AA02507 | 5.253 | 11.206 | 0.809513417 | 0.001939678 | 0.000184729 |
| GOBAR_AA10595 | 5.16 | 10.623 | 0.802850401 | 0.007374267 | 0.000890817 |
| GOBAR_AA22517 | 14.04 | 27.746 | 0.794144244 | 0.007295948 | 0.000879248 |
| GOBAR_AA26374 | 10.403 | 21.53 | 0.792318799 | 0.003725449 | 0.000394831 |
| GOBAR_AA26755 | 20.59 | 42.11 | 0.789823104 | 0.006433466 | 0.000753194 |
| GOBAR_AA19571 | 4.533 | 9.97 | 0.789721998 | 0.002346599 | 0.000230329 |
| GOBAR_AA21940 | 5.263 | 11.03 | 0.787164127 | 0.010332867 | 0.001352087 |
| GOBAR_AA09931 | 8.333 | 16.933 | 0.774316653 | 0.011424061 | 0.001525605 |
| GOBAR_AA12585 | 109.53 | 218.246 | 0.773443876 | 5.38E-06 | 2.37E-07 |
| GOBAR_AA11823 | 4.986 | 9.946 | 0.760406678 | 0.008917641 | 0.001127231 |
| GOBAR_AA38062 | 7.803 | 15.95 | 0.756222438 | 0.008627375 | 0.001082814 |
| GOBAR_AA04632 | 12.006 | 24.826 | 0.746836706 | 0.002178751 | 0.00021121 |
| GOBAR_AA22042 | 22.52 | 44.606 | 0.744018044 | 0.001199976 | 0.000106258 |
| GOBAR_AA21046 | 8.59 | 17.05 | 0.742361662 | 0.039365225 | 0.007117102 |
| GOBAR_AA17519 | 3.75 | 7.393 | 0.741085407 | 0.03064604 | 0.005207853 |
| GOBAR_AA03230 | 39.886 | 77.936 | 0.740488954 | 0.000899581 | 7.62E-05 |
| GOBAR_AA01076 | 8.183 | 15.93 | 0.739289543 | 0.019289424 | 0.0029247 |
| GOBAR_AA23871 | 5.806 | 11.953 | 0.737491131 | 0.028246015 | 0.004699646 |
| GOBAR_AA11063 | 16.653 | 32.57 | 0.734682349 | 0.00645277 | 0.000756014 |
| GOBAR_AA38914 | 21.966 | 42.83 | 0.734004568 | 0.003031189 | 0.000309782 |
| GOBAR_AA01930 | 7.013 | 14.686 | 0.728517509 | 0.045837735 | 0.008622298 |
| GOBAR_AA26827 | 10.32 | 20.226 | 0.727587104 | 0.047714703 | 0.009066204 |
| GOBAR_AA12376 | 4.066 | 8.05 | 0.72612436 | 0.043866206 | 0.008158945 |
| GOBAR_AA38980 | 9.756 | 18.773 | 0.71452186 | 0.000258341 | 1.81E-05 |
| GOBAR_AA00671 | 23.953 | 45.706 | 0.710708524 | 2.37E-05 | 1.21E-06 |
| GOBAR_AA37724 | 42.976 | 81.25 | 0.69862848 | 3.69E-06 | 1.59E-07 |
| GOBAR_AA28518 | 28.693 | 55.43 | 0.693870829 | 0.025536432 | 0.004130145 |
| GOBAR_AA17333 | 6.376 | 11.963 | 0.675330295 | 0.037120499 | 0.006608326 |
| GOBAR_AA25940 | 9.636 | 17.633 | 0.674501926 | 0.002320276 | 0.000227477 |
| GOBAR_AA24818 | 42.373 | 76.59 | 0.664078589 | 0.001932444 | 0.000183928 |
| GOBAR_AA27180 | 7.043 | 12.93 | 0.657439277 | 0.008233237 | 0.001021217 |
| GOBAR_AA10715 | 15.43 | 28.173 | 0.655896649 | 0.013368704 | 0.0018536 |
| GOBAR_AA06338 | 8.346 | 15.553 | 0.650005247 | 0.03456617 | 0.006027787 |
| GOBAR_AA24812 | 13.036 | 25.256 | 0.643880463 | 0.040569987 | 0.007387654 |
| GOBAR_AA24313 | 12.196 | 23.436 | 0.642243045 | 0.043952004 | 0.008179981 |
| GOBAR_AA00889 | 169.916 | 314.13 | 0.638785211 | 0.001045022 | 9.05E-05 |
| GOBAR_AA12876 | 6.853 | 12.806 | 0.638513544 | 0.036184307 | 0.006391491 |
| GOBAR_AA25671 | 7.883 | 15.156 | 0.627004511 | 0.004568569 | 0.000502003 |
| GOBAR_AA27334 | 17.363 | 31.64 | 0.625172057 | 0.019819104 | 0.003018751 |
| GOBAR_AA35814 | 8.106 | 14.563 | 0.619818166 | 0.03173981 | 0.005434064 |
| GOBAR_AA32299 | 8.42 | 14.456 | 0.617770866 | 0.021214977 | 0.003282227 |
| GOBAR_AA38944 | 20.99 | 37.153 | 0.603116672 | 0.042463915 | 0.00783802 |
| GOBAR_AA09268 | 41.61 | 74.143 | 0.595315076 | 0.00766812 | 0.000933624 |
| GOBAR_AA05678 | 12.316 | 21.303 | 0.585290734 | 0.026608341 | 0.004355685 |
| GOBAR_AA07787 | 18.546 | 32.45 | 0.580607851 | 0.002896932 | 0.000293969 |
| GOBAR_AA37376 | 5.956 | 10.493 | 0.58000637 | 0.012556771 | 0.001714908 |
| GOBAR_AA24035 | 85.416 | 149.026 | 0.57562425 | 0.002547105 | 0.00025332 |
| GOBAR_AA02449 | 6.42 | 12.68 | 0.574141672 | 0.033337943 | 0.005780862 |
| GOBAR_AA14727 | 143.376 | 249.806 | 0.573629025 | 0.000397855 | 2.98E-05 |
| GOBAR_AA20027 | 2.716 | 4.936 | 0.57185579 | 0.019565738 | 0.002975073 |
| GOBAR_AA08606 | 26.036 | 45.756 | 0.568201546 | 0.025861359 | 0.004198297 |
| GOBAR_AA09707 | 4.473 | 8.036 | 0.563128072 | 0.014109857 | 0.00198484 |
| GOBAR_AA17327 | 9.9 | 16.59 | 0.559959532 | 0.030217462 | 0.005107091 |
| GOBAR_AA39164 | 11.026 | 18.37 | 0.540783049 | 0.007771612 | 0.000949816 |
| GOBAR_AA08124 | 15.6 | 25.936 | 0.540387301 | 0.023249491 | 0.003681707 |
| GOBAR_AA16020 | 5.903 | 10.143 | 0.539395661 | 0.047288681 | 0.008962161 |
| GOBAR_AA39412 | 71.486 | 120.01 | 0.532397275 | 0.018820157 | 0.002824192 |
| GOBAR_AA11238 | 7.056 | 11.736 | 0.51084691 | 0.036681472 | 0.006503679 |
| GOBAR_AA17876 | 45.99 | 75.7 | 0.498352171 | 0.039476074 | 0.007143985 |
| GOBAR_AA23195 | 71.906 | 116.956 | 0.493890624 | 0.026970391 | 0.004430691 |
| GOBAR_AA29866 | 28.783 | 47.036 | 0.472696544 | 0.04247388 | 0.007842313 |
| GOBAR_AA29557 | 15.956 | 25.83 | 0.469296748 | 0.040981757 | 0.007480393 |
| GOBAR_AA05611 | 17.57 | 28.31 | 0.467874956 | 0.032203755 | 0.005533959 |
| GOBAR_AA36702 | 29.88 | 47.81 | 0.453378591 | 0.014486613 | 0.002055468 |
| GOBAR_AA34625 | 41.866 | 64.273 | 0.378302548 | 0.028973903 | 0.004841677 |
| GOBAR_AA32183 | 54.556 | 48.906 | -0.379356073 | 0.039304903 | 0.007098248 |
| GOBAR_AA32494 | 22.97 | 20.373 | -0.396255329 | 0.031001323 | 0.005282556 |
| GOBAR_AA10194 | 57.933 | 68.61 | -0.400710854 | 0.02399796 | 0.003826883 |
| GOBAR_AA22536 | 16.013 | 13.75 | -0.44867783 | 0.005775468 | 0.000665983 |
| GOBAR_AA24775 | 34.063 | 28.703 | -0.462523404 | 0.024105994 | 0.003849968 |
| GOBAR_AA16182 | 7.573 | 6.383 | -0.475725317 | 0.011977036 | 0.001617396 |
| GOBAR_AA05879 | 28.03 | 23.276 | -0.496435839 | 0.012863152 | 0.001767155 |
| GOBAR_AA19953 | 38.926 | 32.62 | -0.501270076 | 0.015129371 | 0.002163711 |
| GOBAR_AA01569 | 16.77 | 13.863 | -0.501956813 | 0.017168164 | 0.002523228 |
| GOBAR_AA09729 | 11.446 | 9.286 | -0.522902 | 0.01347316 | 0.001873532 |
| GOBAR_AA05020 | 11.183 | 9.06 | -0.544836202 | 0.025636531 | 0.004152172 |
| GOBAR_AA15974 | 39.966 | 33.143 | -0.552601261 | 0.003729314 | 0.000395564 |
| GOBAR_AA35697 | 13.583 | 10.77 | -0.564303056 | 0.012437199 | 0.001694986 |
| GOBAR_AA18226 | 21.146 | 16.516 | -0.564891848 | 0.045366052 | 0.00849819 |
| GOBAR_AA15209 | 13.683 | 10.76 | -0.571571024 | 0.036135341 | 0.006380754 |
| GOBAR_AA27486 | 55.826 | 43.016 | -0.577489293 | 0.023249386 | 0.003680252 |
| GOBAR_AA09790 | 31.286 | 25.236 | -0.577857727 | 0.009163458 | 0.001168891 |
| GOBAR_AA05749 | 16.563 | 12.496 | -0.605672869 | 0.005791047 | 0.000667947 |
| GOBAR_AA05082 | 35 | 26.666 | -0.611792871 | 0.000710527 | 5.77E-05 |
| GOBAR_AA11269 | 85.346 | 64.796 | -0.616250643 | 0.021415145 | 0.003323093 |
| GOBAR_AA37267 | 13.253 | 9.92 | -0.62411226 | 0.017244695 | 0.002536966 |
| GOBAR_AA31804 | 11.58 | 8.7 | -0.626417022 | 0.003489903 | 0.00036513 |
| GOBAR_AA06265 | 41.423 | 31.476 | -0.629974376 | 0.001561597 | 0.000144481 |
| GOBAR_AA00604 | 36.553 | 27.476 | -0.631478194 | 0.015474139 | 0.002225086 |
| GOBAR_AA37054 | 32.573 | 24.056 | -0.636026804 | 0.042239538 | 0.007785159 |
| GOBAR_AA36948 | 28.216 | 21.006 | -0.636570521 | 0.03749489 | 0.006704219 |
| GOBAR_AA13445 | 49.616 | 37.18 | -0.649575033 | 0.003229815 | 0.0003326 |
| GOBAR_AA10987 | 11.263 | 7.35 | -0.65204744 | 0.007071313 | 0.000846866 |
| GOBAR_AA21189 | 7.456 | 5.106 | -0.652317251 | 0.048629716 | 0.009299408 |
| GOBAR_AA01991 | 30.633 | 22.716 | -0.660815462 | 0.005137592 | 0.000579945 |
| GOBAR_AA39438 | 42.343 | 29.003 | -0.665478097 | 0.013310719 | 0.001841715 |
| GOBAR_AA25555 | 8.76 | 6.396 | -0.673422288 | 0.004369407 | 0.000477468 |
| GOBAR_AA30173 | 71.42 | 52.43 | -0.678471904 | 4.84E-07 | 1.74E-08 |
| GOBAR_AA16963 | 12.196 | 8.883 | -0.682669517 | 0.002128403 | 0.00020553 |
| GOBAR_AA30196 | 53.563 | 36.933 | -0.682808749 | 0.019598142 | 0.002981132 |
| GOBAR_AA20311 | 21.136 | 15.96 | -0.683700424 | 0.007937549 | 0.000976746 |
| GOBAR_AA23754 | 3.603 | 2.576 | -0.700954089 | 0.003257824 | 0.000336324 |
| GOBAR_AA07983 | 13.293 | 9.016 | -0.706083074 | 0.013903366 | 0.001947813 |
| GOBAR_AA07102 | 58.703 | 42.056 | -0.706695502 | 0.000254906 | 1.78E-05 |
| GOBAR_AA09691 | 22.893 | 16.636 | -0.71031798 | 0.000244228 | 1.69E-05 |
| GOBAR_AA27828 | 8.446 | 6.01 | -0.714630432 | 0.001259234 | 0.000112012 |
| GOBAR_AA14379 | 44.34 | 31.503 | -0.71734302 | 4.69E-05 | 2.60E-06 |
| GOBAR_AA37591 | 13.083 | 9.023 | -0.733967084 | 0.007365719 | 0.00088885 |
| GOBAR_AA25641 | 19.086 | 13.43 | -0.738106752 | 3.98E-05 | 2.17E-06 |
| GOBAR_AA19189 | 20.12 | 15.543 | -0.763686439 | 1.64E-05 | 8.10E-07 |
| GOBAR_AA12008 | 590.606 | 405.15 | -0.766646039 | 0.03251201 | 0.005599139 |
| GOBAR_AA27142 | 65.23 | 44.723 | -0.770549527 | 0.000498829 | 3.84E-05 |
| GOBAR_AA23667 | 8.21 | 5.583 | -0.771084593 | 0.003414444 | 0.000356249 |
| GOBAR_AA24691 | 15.66 | 10.976 | -0.77337239 | 0.020086678 | 0.003072271 |
| GOBAR_AA38360 | 7.16 | 4.843 | -0.78585859 | 8.00E-05 | 4.71E-06 |
| GOBAR_AA22784 | 25.446 | 17.033 | -0.788414898 | 0.002019123 | 0.000193344 |
| GOBAR_AA13954 | 8.306 | 5.536 | -0.791980972 | 0.02505891 | 0.004027493 |
| GOBAR_AA17117 | 17.553 | 11.84 | -0.801496842 | 0.000791676 | 6.55E-05 |
| GOBAR_AA02747 | 18.21 | 11.4 | -0.803039445 | 0.000408426 | 3.06E-05 |
| GOBAR_AA34250 | 55.273 | 36.126 | -0.808596095 | 6.56E-07 | 2.40E-08 |
| GOBAR_AA34173 | 34.116 | 21.006 | -0.81045071 | 0.006103217 | 0.000709765 |
| GOBAR_AA39552 | 22.423 | 14.796 | -0.817152429 | 0.000322143 | 2.33E-05 |
| GOBAR_AA38154 | 12.39 | 8.126 | -0.821896764 | 0.000175281 | 1.14E-05 |
| GOBAR_AA23805 | 297.216 | 191.283 | -0.831506623 | 0.02654415 | 0.004340577 |
| GOBAR_AA19813 | 16.253 | 10.493 | -0.835523778 | 0.002423077 | 0.000238885 |
| GOBAR_AA38168 | 18.806 | 12.296 | -0.836221905 | 0.000267586 | 1.88E-05 |
| GOBAR_AA09738 | 26.993 | 17.536 | -0.845652272 | 5.99E-05 | 3.41E-06 |
| GOBAR_AA29749 | 8.183 | 5.253 | -0.851485011 | 0.001289722 | 0.000115341 |
| GOBAR_AA08350 | 12.153 | 7.766 | -0.854028659 | 0.00053399 | 4.15E-05 |
| GOBAR_AA01019 | 11.65 | 7.44 | -0.854822901 | 0.048885745 | 0.009377805 |
| GOBAR_AA13847 | 7.916 | 5.046 | -0.856700637 | 0.003700523 | 0.000391525 |
| GOBAR_AA28338 | 13.24 | 7.956 | -0.866846728 | 0.000417149 | 3.14E-05 |
| GOBAR_AA24000 | 18.7 | 11.646 | -0.869900766 | 0.024408704 | 0.003906775 |
| GOBAR_AA32355 | 14.733 | 9.383 | -0.873328291 | 0.04541663 | 0.008514224 |
| GOBAR_AA00055 | 18.563 | 11.806 | -0.874794418 | 0.000164697 | 1.06E-05 |
| GOBAR_AA22131 | 19.763 | 12.083 | -0.881159373 | 0.000168353 | 1.09E-05 |
| GOBAR_AA23115 | 7.3 | 4.523 | -0.894198695 | 1.54E-06 | 6.10E-08 |
| GOBAR_AA38830 | 18.34 | 11.483 | -0.904050791 | 0.001803722 | 0.000170247 |
| GOBAR_AA05468 | 99.65 | 75.42 | -0.906547447 | 3.09E-05 | 1.63E-06 |
| GOBAR_AA01134 | 99.216 | 58.786 | -0.907304131 | 0.009474542 | 0.001216783 |
| GOBAR_AA02218 | 69.77 | 38.96 | -0.909630978 | 0.004849079 | 0.000539129 |
| GOBAR_AA02663 | 10.213 | 6.313 | -0.917096397 | 0.018674538 | 0.002800722 |
| GOBAR_AA03934 | 12.026 | 7.26 | -0.926964419 | 0.01578146 | 0.002278324 |
| GOBAR_AA11108 | 42.526 | 25.3 | -0.942715812 | 0.001846577 | 0.000174795 |
| GOBAR_AA23099 | 10.39 | 6.48 | -0.943420819 | 0.005111091 | 0.000575494 |
| GOBAR_AA33982 | 4.886 | 2.696 | -0.950297911 | 0.034304578 | 0.005972261 |
| GOBAR_AA35832 | 58.73 | 35.293 | -0.958934852 | 4.73E-07 | 1.70E-08 |
| GOBAR_AA38613 | 40.78 | 24.03 | -0.970046298 | 1.31E-06 | 5.16E-08 |
| GOBAR_AA14312 | 20.566 | 11.853 | -0.971243995 | 1.84E-06 | 7.47E-08 |
| GOBAR_AA28985 | 13.036 | 7.636 | -0.972845516 | 0.014859204 | 0.002117078 |
| GOBAR_AA28168 | 41.673 | 24.506 | -0.988927941 | 0.004784526 | 0.000530432 |
| GOBAR_AA14979 | 55.6 | 32.236 | -0.989593227 | 1.76E-10 | 3.91E-12 |
| GOBAR_AA00352 | 37.373 | 22.956 | -0.998864094 | 4.60E-07 | 1.64E-08 |
| GOBAR_AA18771 | 10.07 | 5.743 | -1.015020637 | 0.003189512 | 0.000327914 |
| GOBAR_AA08410 | 2.19 | 1.25 | -1.017426375 | 0.012232703 | 0.001660049 |
| GOBAR_AA28294 | 3.716 | 2.203 | -1.023550852 | 0.026881613 | 0.004410513 |
| GOBAR_AA11838 | 8.843 | 5.173 | -1.041862207 | 6.94E-05 | 4.04E-06 |
| GOBAR_AA11581 | 19.693 | 11.043 | -1.042647852 | 7.12E-06 | 3.23E-07 |
| GOBAR_AA15131 | 5.316 | 2.853 | -1.051177014 | 0.008558065 | 0.001071396 |
| GOBAR_AA03260 | 127.503 | 70.89 | -1.051504968 | 2.02E-07 | 6.80E-09 |
| GOBAR_AA28430 | 5.566 | 2.993 | -1.055336573 | 0.049719802 | 0.009583995 |
| GOBAR_AA40283 | 31.766 | 17.533 | -1.056609388 | 4.04E-05 | 2.20E-06 |
| GOBAR_AA09283 | 4.03 | 2.2 | -1.063191444 | 0.014170151 | 0.00199565 |
| GOBAR_AA28913 | 6.67 | 3.48 | -1.079714757 | 0.032368329 | 0.00556411 |
| GOBAR_AA30381 | 5.02 | 2.636 | -1.12011561 | 0.022701527 | 0.003573856 |
| GOBAR_AA33387 | 5.77 | 2.913 | -1.148153261 | 0.005374452 | 0.000613065 |
| GOBAR_AA38014 | 271.076 | 147.903 | -1.153225656 | 1.21E-06 | 4.73E-08 |
| GOBAR_AA24944 | 83.75 | 44.083 | -1.180083933 | 0.000767986 | 6.31E-05 |
| GOBAR_AA05694 | 87.283 | 44.163 | -1.182316652 | 1.89E-06 | 7.67E-08 |
| GOBAR_AA38583 | 140.246 | 71.75 | -1.196034958 | 9.62E-06 | 4.50E-07 |
| GOBAR_AA40168 | 33.486 | 16.833 | -1.196096056 | 0.000694332 | 5.62E-05 |
| GOBAR_AA39922 | 6.453 | 3.206 | -1.209364829 | 0.000382945 | 2.85E-05 |
| GOBAR_AA12578 | 70.016 | 36.3 | -1.209525262 | 4.27E-12 | 8.01E-14 |
| GOBAR_AA37678 | 18.063 | 8.933 | -1.212305313 | 0.00023123 | 1.58E-05 |
| GOBAR_AA39918 | 3.763 | 1.846 | -1.230490023 | 0.001575269 | 0.000145792 |
| GOBAR_AA29218 | 93.163 | 44.993 | -1.236076332 | 8.06E-05 | 4.75E-06 |
| GOBAR_AA00953 | 3.163 | 1.47 | -1.282605708 | 0.010104051 | 0.001316892 |
| GOBAR_AA20760 | 3.71 | 1.663 | -1.284561514 | 0.02468735 | 0.003960644 |
| GOBAR_AA17527 | 3.616 | 1.696 | -1.293901924 | 0.041235293 | 0.007544538 |
| GOBAR_AA12977 | 5.493 | 2.39 | -1.297759015 | 0.031313973 | 0.005344625 |
| GOBAR_AA35355 | 96.713 | 44.82 | -1.301496692 | 1.42E-06 | 5.64E-08 |
| GOBAR_AA32144 | 11.626 | 5.34 | -1.33857814 | 0.001810001 | 0.000171019 |
| GOBAR_AA02249 | 8.126 | 3.616 | -1.343614748 | 6.59E-06 | 2.96E-07 |
| GOBAR_AA24010 | 64.043 | 29.716 | -1.369388471 | 2.49E-17 | 2.94E-19 |
| GOBAR_AA26877 | 28.203 | 13.303 | -1.370922106 | 3.33E-09 | 8.74E-11 |
| GOBAR_AA17952 | 47.926 | 20.693 | -1.404463692 | 1.54E-05 | 7.56E-07 |
| GOBAR_AA26509 | 27.273 | 11.736 | -1.420754455 | 2.36E-12 | 4.29E-14 |
| GOBAR_AA08598 | 144.443 | 61.153 | -1.420954584 | 0.009939835 | 0.001289402 |
| GOBAR_AA05331 | 0.823 | 0.336 | -1.426679714 | 0.042043568 | 0.007734928 |
| GOBAR_AA01056 | 21.446 | 9.01 | -1.443486017 | 1.04E-06 | 3.98E-08 |
| GOBAR_AA21395 | 7.213 | 3.016 | -1.444738784 | 0.00058501 | 4.61E-05 |
| GOBAR_AA27175 | 9.773 | 4.133 | -1.457939275 | 1.29E-08 | 3.65E-10 |
| GOBAR_AA08603 | 2.873 | 1.113 | -1.475145456 | 0.025552317 | 0.004134104 |
| GOBAR_AA27164 | 3.956 | 1.55 | -1.520135418 | 0.00524034 | 0.000594289 |
| GOBAR_AA21188 | 10.503 | 3.873 | -1.552514085 | 0.012142096 | 0.00164438 |
| GOBAR_AA12786 | 466.706 | 179.013 | -1.570768611 | 7.22E-06 | 3.28E-07 |
| GOBAR_AA20380 | 13.193 | 4.853 | -1.593111729 | 0.000254716 | 1.77E-05 |
| GOBAR_AA23502 | 2.53 | 0.903 | -1.599704615 | 0.006807551 | 0.000807806 |
| GOBAR_AA20016 | 65.753 | 24.65 | -1.606112255 | 2.44E-11 | 4.93E-13 |
| GOBAR_AA24413 | 9.45 | 3.65 | -1.62523452 | 2.79E-13 | 4.61E-15 |
| GOBAR_AA27427 | 8.266 | 2.93 | -1.630824758 | 2.87E-06 | 1.21E-07 |
| GOBAR_AA09278 | 0.84 | 0.276 | -1.656769601 | 0.015524761 | 0.002235134 |
| GOBAR_AA14037 | 9.75 | 4.293 | -1.675143996 | 6.03E-16 | 7.84E-18 |
| GOBAR_AA06873 | 4.406 | 1.493 | -1.687631039 | 0.002042058 | 0.000196116 |
| GOBAR_AA04421 | 157.296 | 53.43 | -1.764827458 | 5.06E-20 | 4.93E-22 |
| GOBAR_AA08019 | 3.183 | 1.086 | -1.765427221 | 0.000181765 | 1.20E-05 |
| GOBAR_AA35155 | 2.193 | 0.653 | -1.775315218 | 0.01107709 | 0.001469569 |
| GOBAR_AA30841 | 7.12 | 2.31 | -1.784219584 | 8.69E-08 | 2.75E-09 |
| GOBAR_AA21664 | 4.29 | 1.376 | -1.791118969 | 0.005122277 | 0.000577641 |
| GOBAR_AA23674 | 100.376 | 30.743 | -1.800929396 | 0.000130208 | 8.14E-06 |
| GOBAR_AA39373 | 121.793 | 39.483 | -1.811055793 | 9.77E-12 | 1.90E-13 |
| GOBAR_AA08176 | 72.81 | 23.693 | -1.819958286 | 8.92E-13 | 1.56E-14 |
| GOBAR_AA04298 | 189.073 | 61.096 | -1.835270935 | 1.66E-22 | 1.34E-24 |
| GOBAR_AA39940 | 3.736 | 1.103 | -1.924663759 | 4.48E-08 | 1.35E-09 |
| GOBAR_AA11014 | 0.74 | 0.193 | -1.962418114 | 0.002403293 | 0.000236587 |
| GOBAR_AA38025 | 1.62 | 0.4 | -1.965862208 | 0.00662764 | 0.000781096 |
| GOBAR_AA20705 | 4.086 | 1.16 | -1.96728627 | 0.000211087 | 1.42E-05 |
| GOBAR_AA11752 | 109.886 | 32.603 | -1.975616677 | 1.63E-12 | 2.91E-14 |
| GOBAR_AA37155 | 93.183 | 26.653 | -1.98840379 | 3.92E-10 | 9.13E-12 |
| GOBAR_AA05154 | 2.923 | 0.836 | -1.994714224 | 0.000161151 | 1.04E-05 |
| GOBAR_AA08801 | 0.616 | 0.136 | -2.021591429 | 0.021493877 | 0.003339656 |
| GOBAR_AA32186 | 0.973 | 0.213 | -2.054003909 | 0.011823011 | 0.001590791 |
| GOBAR_AA12233 | 14.143 | 3.736 | -2.059700091 | 8.27E-12 | 1.60E-13 |
| GOBAR_AA38713 | 21.656 | 5.39 | -2.149193167 | 5.06E-18 | 5.66E-20 |
| GOBAR_AA33975 | 1.873 | 0.39 | -2.166247759 | 0.003563403 | 0.000374364 |
| GOBAR_AA34785 | 4.17 | 0.943 | -2.172115414 | 0.00030982 | 2.22E-05 |
| GOBAR_AA31103 | 119.336 | 28.536 | -2.207064399 | 1.42E-09 | 3.59E-11 |
| GOBAR_AA25392 | 181.18 | 43.25 | -2.236421619 | 1.13E-10 | 2.47E-12 |
| GOBAR_AA35548 | 0.2 | 0.023 | -2.249270897 | 0.049827407 | 0.009614578 |
| GOBAR_AA24900 | 1.583 | 0.266 | -2.26496174 | 0.010034681 | 0.001304372 |
| GOBAR_AA35770 | 7.283 | 1.586 | -2.271533649 | 0.000229721 | 1.57E-05 |
| GOBAR_AA02489 | 25.37 | 5.86 | -2.287775084 | 3.60E-14 | 5.44E-16 |
| GOBAR_AA09751 | 1.576 | 0.33 | -2.309295489 | 2.97E-05 | 1.56E-06 |
| GOBAR_AA38655 | 0.45 | 0.033 | -2.391689418 | 0.037267099 | 0.006642691 |
| GOBAR_AA23957 | 0.673 | 0.113 | -2.392137348 | 0.0016567 | 0.000154429 |
| GOBAR_AA17353 | 123.99 | 24.766 | -2.487331524 | 2.55E-14 | 3.81E-16 |
| GOBAR_AA36042 | 26.68 | 6.103 | -2.491839581 | 4.71E-45 | 1.59E-47 |
| GOBAR_AA26026 | 0.966 | 0.066 | -2.521938516 | 0.022629546 | 0.003559256 |
| GOBAR_AA06604 | 149.13 | 26.92 | -2.594597507 | 1.34E-57 | 3.05E-60 |
| GOBAR_AA07239 | 41.95 | 7.293 | -2.636319724 | 7.89E-16 | 1.05E-17 |
| GOBAR_AA12516 | 12.906 | 2.046 | -2.748296303 | 5.14E-12 | 9.78E-14 |
| GOBAR_AA02034 | 102.573 | 15.293 | -2.828417532 | 9.14E-11 | 1.98E-12 |
| GOBAR_AA12958 | 17.086 | 2.536 | -2.849271425 | 4.97E-09 | 1.33E-10 |
| GOBAR_AA13677 | 29.926 | 4.686 | -2.874781047 | 8.08E-55 | 1.98E-57 |
| GOBAR_AA38757 | 4.69 | 0.626 | -2.879137027 | 6.00E-07 | 2.19E-08 |
| GOBAR_AA08470 | 1.1 | 0.11 | -3.019589925 | 2.59E-05 | 1.34E-06 |
| GOBAR_AA05496 | 4.186 | 0.243 | -3.183803422 | 0.000678471 | 5.45E-05 |
| GOBAR_AA33158 | 8.066 | 0.83 | -3.276714754 | 2.67E-09 | 6.93E-11 |
| GOBAR_AA07175 | 2.863 | 0.326 | -3.29301887 | 3.50E-20 | 3.38E-22 |
| GOBAR_AA07466 | 1.97 | 0.036 | -3.395903303 | 0.001368209 | 0.000123308 |
| GOBAR_AA37328 | 120.46 | 0.113 | -3.41336168 | 0.001927558 | 0.000183407 |
| GOBAR_AA28301 | 3.383 | 0.036 | -3.619316927 | 0.00048519 | 3.73E-05 |
| GOBAR_AA14697 | 21.506 | 1.23 | -3.751616697 | 2.19E-08 | 6.36E-10 |
| GOBAR_AA14083 | 3.733 | 0.173 | -3.888090414 | 9.46E-08 | 3.01E-09 |
| GOBAR_AA10909 | 6.9 | 0.35 | -4.158372824 | 3.14E-15 | 4.37E-17 |
| GOBAR_AA13312 | 10.723 | 0.323 | -4.257191283 | 2.01E-08 | 5.79E-10 |
| GOBAR_AA06903 | 25.56 | 0.99 | -4.303706196 | 4.97E-11 | 1.05E-12 |
| GOBAR_AA27390 | 11.303 | 0.436 | -4.685813246 | 4.05E-32 | 2.15E-34 |
| GOBAR_AA32211 | 109.953 | 4.336 | -4.755523461 | 4.31E-50 | 1.31E-52 |
| GOBAR_AA26327 | 20.353 | 0.77 | -4.883391585 | 2.11E-43 | 7.76E-46 |
| GOBAR_AA30876 | 16.66 | 0.563 | -4.951014894 | 3.23E-50 | 9.60E-53 |
| GOBAR_AA36953 | 0.896 | 0 | -4.961428427 | 2.60E-08 | 7.61E-10 |
| GOBAR_AA06434 | 37.286 | 1.163 | -5.004959899 | 1.65E-77 | 2.33E-80 |
| GOBAR_AA04822 | 17.3 | 0.44 | -5.093175218 | 5.92E-22 | 5.01E-24 |
| GOBAR_AA05165 | 24.783 | 0.703 | -5.160851123 | 2.23E-50 | 6.50E-53 |
| GOBAR_AA00006 | 6.076 | 0.153 | -5.209309766 | 2.08E-20 | 1.95E-22 |
| GOBAR_AA31454 | 24.233 | 0.196 | -5.484249796 | 3.42E-13 | 5.71E-15 |
| GOBAR_AA08471 | 20.706 | 0.193 | -5.622944204 | 1.80E-15 | 2.45E-17 |
| GOBAR_AA19621 | 13.87 | 0.186 | -6.361603731 | 8.59E-68 | 1.51E-70 |
| GOBAR_AA39823 | 138.096 | 2.373 | -6.726091765 | 2.16E-172 | 2.49E-176 |
| GOBAR_AA03072 | 180.686 | 0.48 | -6.953101948 | 1.02E-20 | 9.30E-23 |
