# Supplementary figures and images for: Integrated Methylome and Transcriptome Analysis Widen the Knowledge of Cytoplasmic Male Sterility in Cotton (Gossypium barbadense L.)
Source: Front Plant Sci. 2022 Apr 27;13:770098. doi: 10.3389/fpls.2022.770098 (PMC9093596; doi:10.3389/fpls.2022.770098)

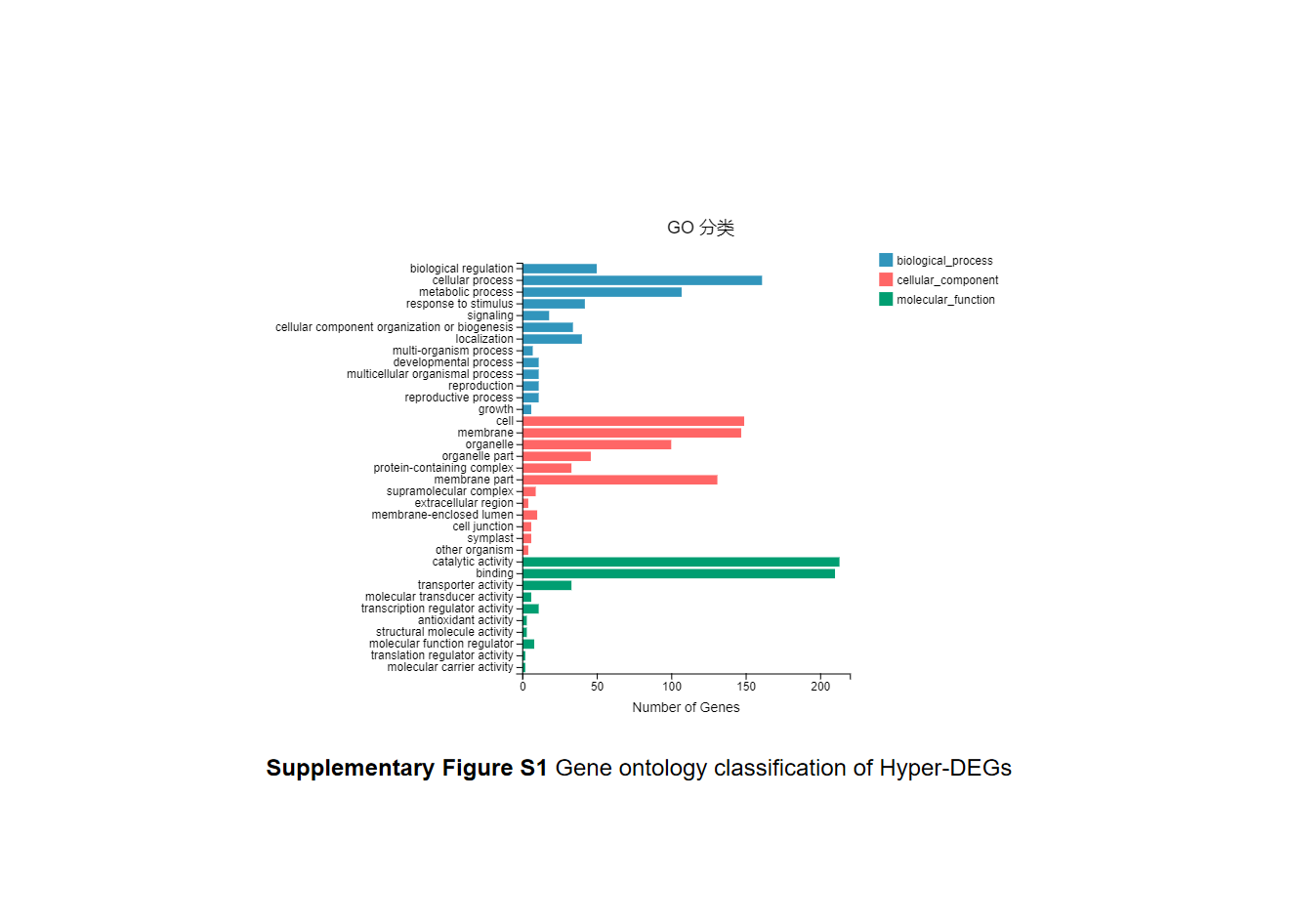

Supplement: Supplementary file 12 [file Image_1.TIF]

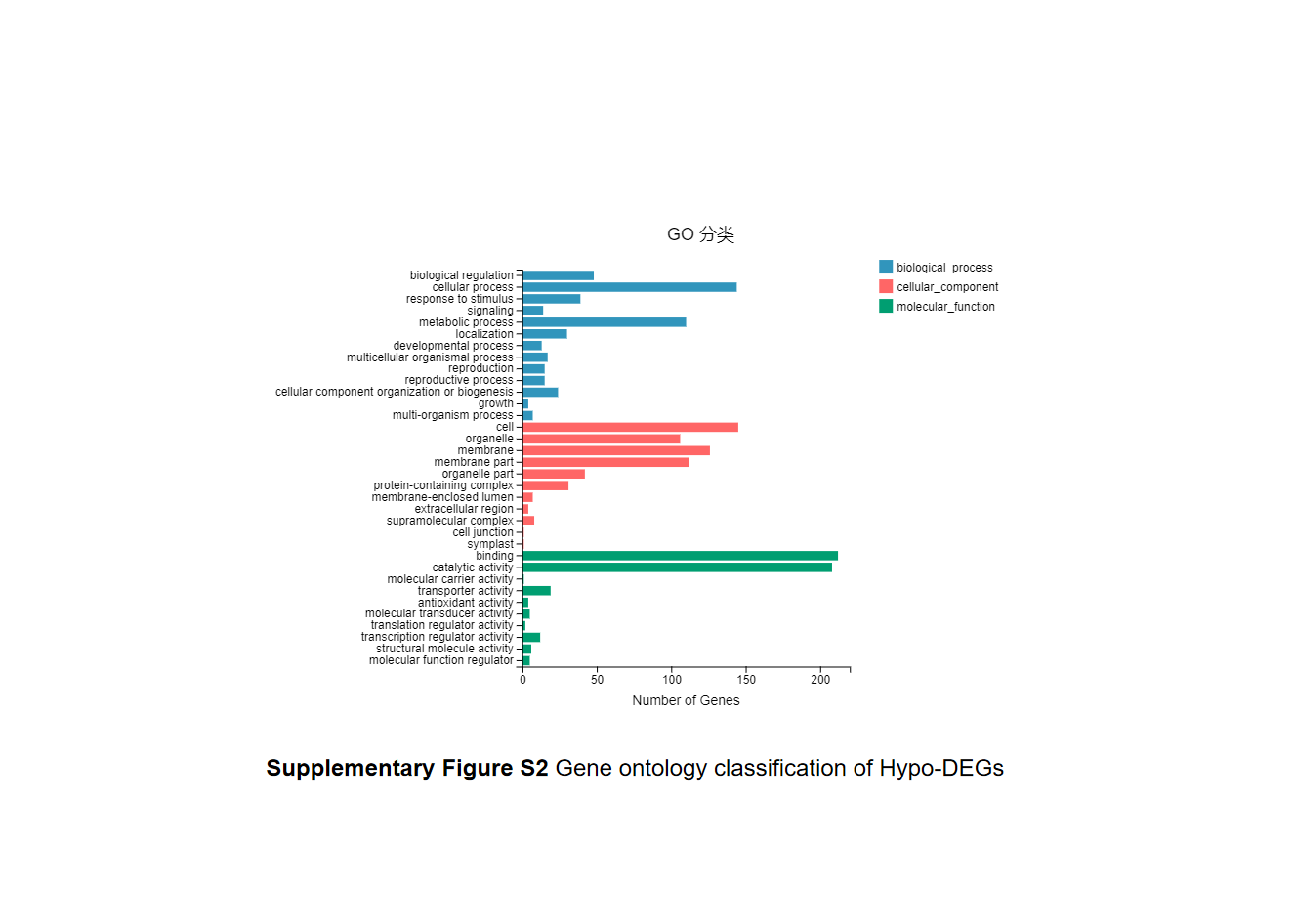

Supplement: Supplementary file 13 [file Image_2.TIF]

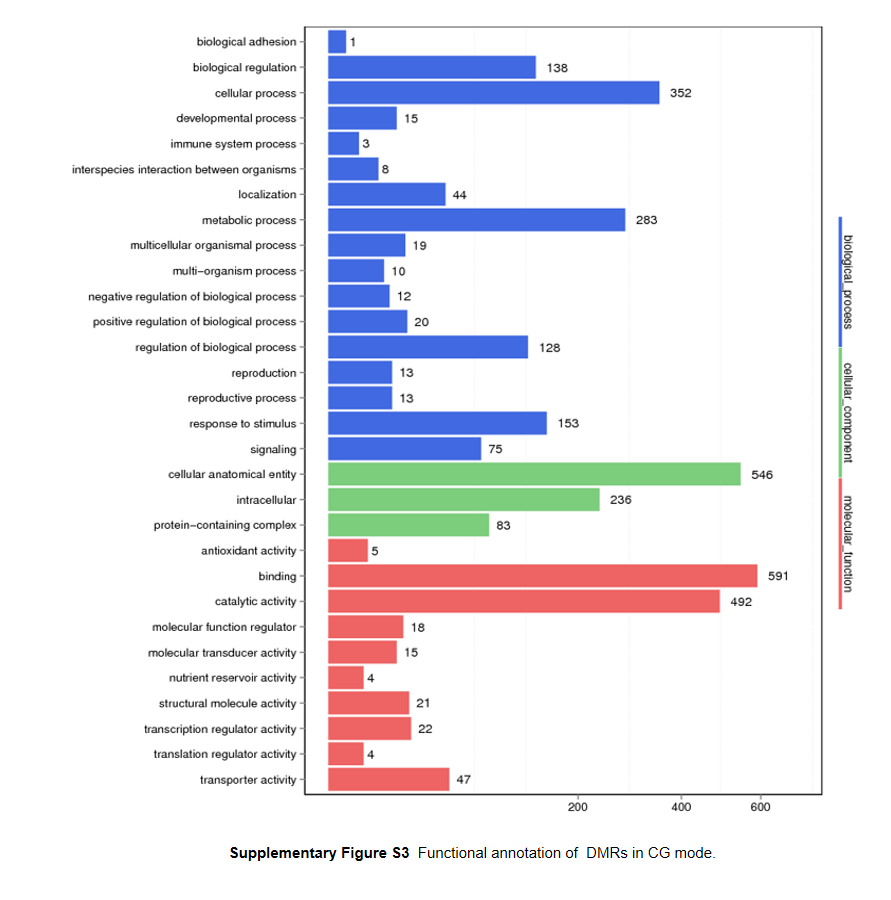

Supplement: Supplementary file 14 [file Image_3.TIF]

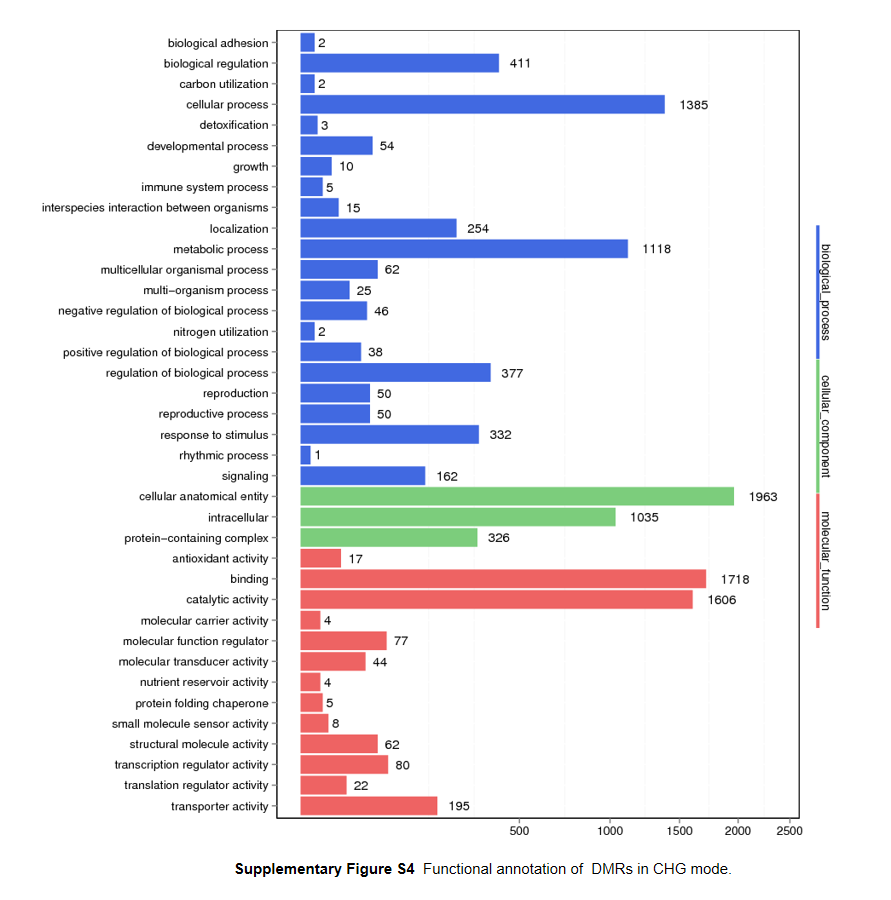

Supplement: Supplementary file 15 [file Image_4.TIF]
